# Asymmetrical lineage introgression and recombination in populations of *Aspergillus flavus*: Implications for biological control

**DOI:** 10.1371/journal.pone.0276556

**Published:** 2022-10-27

**Authors:** Megan S. Molo, James B. White, Vicki Cornish, Richard M. Gell, Oliver Baars, Rakhi Singh, Mary Anna Carbone, Thomas Isakeit, Kiersten A. Wise, Charles P. Woloshuk, Burton H. Bluhm, Bruce W. Horn, Ron W. Heiniger, Ignazio Carbone

**Affiliations:** 1 Department of Entomology and Plant Pathology, Center for Integrated Fungal Research, North Carolina State University, Raleigh, NC, United States of America; 2 Program of Genetics, North Carolina State University, Raleigh, North Carolina, United States of America; 3 Center for Integrated Fungal Research and Department of Plant and Microbial Biology, North Carolina State University, Raleigh, NC, United States of America; 4 Department of Plant Pathology and Microbiology, Texas AgriLife Extension Service, Texas A&M University, College Station, TX, United States of America; 5 Department of Plant Pathology, University of Kentucky, Princeton, KY, United States of America; 6 Department of Plant Pathology and Botany, Purdue University, West Lafayette, IN, United States of America; 7 University of Arkansas Division of Agriculture, Department of Entomology and Plant Pathology, Fayetteville, AR, United States of America; 8 United States Department of Agriculture, Agriculture Research Service, Dawson, GA, United States of America; 9 Department of Crop and Soil Sciences, North Carolina State University, Raleigh, NC, United States of America; J Craig Venter Institute, UNITED STATES

## Abstract

*Aspergillus flavus* is an agriculturally important fungus that causes ear rot of maize and produces aflatoxins, of which B_1_ is the most carcinogenic naturally-produced compound. In the US, the management of aflatoxins includes the deployment of biological control agents that comprise two nonaflatoxigenic *A*. *flavus* strains, either Afla-Guard (member of lineage IB) or AF36 (lineage IC). We used genotyping-by-sequencing to examine the influence of both biocontrol agents on native populations of *A*. *flavus* in cornfields in Texas, North Carolina, Arkansas, and Indiana. This study examined up to 27,529 single-nucleotide polymorphisms (SNPs) in a total of 815 *A*. *flavus* isolates, and 353 genome-wide haplotypes sampled before biocontrol application, three months after biocontrol application, and up to three years after initial application. Here, we report that the two distinct *A*. *flavus* evolutionary lineages IB and IC differ significantly in their frequency distributions across states. We provide evidence of increased unidirectional gene flow from lineage IB into IC, inferred to be due to the applied Afla-Guard biocontrol strain. Genetic exchange and recombination of biocontrol strains with native strains was detected in as little as three months after biocontrol application and up to one and three years later. There was limited inter-lineage migration in the untreated fields. These findings suggest that biocontrol products that include strains from lineage IB offer the greatest potential for sustained reductions in aflatoxin levels over several years. This knowledge has important implications for developing new biocontrol strategies.

## Introduction

Many fungi produce toxic secondary metabolites or mycotoxins with negative health impacts on humans and animals [1,2]. An agriculturally important mycotoxin producer is *Aspergillus flavus*, which causes ear rot of maize and produces aflatoxins. Aflatoxins are polyketide secondary metabolites produced by *A*. *flavus* and several other species in *Aspergillus* section *Flavi*. *A*. *flavus* is a soilborne filamentous fungus and its aflatoxins commonly contaminates many economically important crops, such as corn, peanuts, cotton, tree nuts, and spices including curry and chili [3–8]. In addition to aflatoxins, *A*. *flavus* also has potential to produce cyclopiazonic acid (CPA), an indole-tetramic acid that targets the liver, kidneys, and gastrointestinal tract of animals [9–11]. Because of the adverse effects on human and animal health, the US Food and Drug Administration (FDA) strictly regulates the levels of aflatoxins in grain. Grains must yield levels of aflatoxin below 20 parts per billion (ppb) for human consumption and 100 ppb for animal feed [12]. Crops exceeding these limits are rejected resulting in millions of dollars in losses [13].

Current management of aflatoxins includes the application of nonaflatoxigenic *A*. *flavus* biological control agents, either Afla-Guard (active ingredient = NRRL 21882) or AF36 Prevail (formerly AF36, active ingredient = NRRL 18543), that competitively interfere with native aflatoxigenic *A*. *flavus* strains for space and resources in corn ears [10,14]. Biocontrol strategies have been shown to effectively reduce aflatoxin contamination and compared with untreated controls result in aflatoxin reduction by approximately 70–99% [15–19]. Afla-Guard was registered with the US Environmental Protection Agency (EPA) in 2004 for reduction of aflatoxin contamination in peanuts and corn in the United States [20]. *A*. *flavus* NRRL 21882 is nonaflatoxigenic and missing the entire aflatoxin and CPA gene clusters [10,21]. AF36 was registered with the EPA in 2003 for use on cotton and corn in Arizona and Texas [22]. A nonsense mutation in the *pksA* (*aflC*) gene early in the aflatoxin biosynthetic pathway renders *A*. *flavus* NRRL 18543 nonaflatoxigenic [10,23]. Moreover, NRRL 18543 produces CPA and its application has been shown to significantly increase CPA contamination in grain [24]. While CPA is currently unregulated by the FDA, it has been linked to adverse health effects in humans and animals [9].

Aflatoxin production is a polygenic trait and influenced by multiple environmental factors that also play a role in crop health, such as elevated temperature and water stress [25], insect damage [26], soil nitrogen [27–29], pH [30] and carbon availability [31]. The persistence of mycotoxigenicity in field populations also has a genetic basis, and both aflatoxin and CPA clusters show high heritability from parents to progeny strains [32,33]. Aflatoxin biosynthesis is controlled by enzymes encoded by approximately 30 genes located in the 75-kb subtelomeric region of chromosome 3 [34]. Sequence polymorphisms and deletions in the aflatoxin gene cluster, as well as genome-wide variation, separate *A*. *flavus* strains into two distinct evolutionary lineages, IB and IC [10,32]. Lineage IB is composed of strains with partial and absent aflatoxin gene clusters, and full-cluster strains with many fixed polymorphisms [10], resulting primarily in strains that are nonaflatoxigenic or strains producing very low amounts of aflatoxin [35,36]. Lineage IC includes predominantly full cluster strains that are aflatoxigenic and harbor extensive quantitative variation in aflatoxin production, from very high aflatoxin producers to nonproducers [10].

A population genomics study by Drott and coworkers [37] examined 910,777 SNPs across 94 isolates of *A*. *flavus* and delineated three populations (A, B, and C) that could not be unambiguously assigned to lineages IB and IC based on phylogenetic analysis of *omtA (= aflP)*, *trpC*, and *amdS* [35]. Some isolates in populations A and C showed affinity to IB and IC and all isolates in population B were proposed to form a new group in the IA lineage. Further population genetic and toxin analysis showed that populations A, B, and C differed in aflatoxin production, frequency of recombination, and genetic diversity. Relatively frequent sex was found in population A which had characteristics that were very similar to lineage IC; population B produced significantly less aflatoxin and comprised many isolates with deletions in the aflatoxin cluster which is the hallmark of lineage IB; population C was mostly nonaflatoxigenic and sister to population A with affinities to both lineage IB and IC. By contrast, a large-scale population genetic study by Moore and coworkers [36] found that multilocus sequence typing of five loci (*aflM*, *aflW*, *mfs*, *trpC*, and *amdS*) was sufficient to resolve *A*. *flavus* lineage and species relationships in *Aspergillus* section *Flavi*. The study applied multilocus phylogenetic inference and admixture analysis to 1,304 isolates sampled across six species: *A*. *flavus* (L, S_B_, S_BG_), *A*. *parasiticus*, *A*. *nomius*, *A*. *caelatus*, *A*. *tamarii*, *and A*. *alliaceus*. Seven distinct clusters were inferred that were associated with species and in the case of *A*. *flavus* distinct clusters were associated with morphotypes (L-and S-strains) and aflatoxin chemotypes.

More recently Guo and coworkers [38] used the Structured Coalescent with Ancestral Recombination (SCAR) model to estimate recombination and migration rates of lineages IB and IC between *A*. *flavus* subpopulations in Texas, North Carolina, Arkansas, and Indiana. This is a novel approach that jointly estimates the ancestral recombination graph (ARG) and migration patterns along a chromosome under a structured coalescent model which allows lineages to migrate between different subpopulations. An examination of SNPs on *A*. *flavus* chromosome 3 revealed a total of 190 recombination events in lineage IB compared to 774 for lineage IC, and recombination rates of 2.28 × 10^−09^ and 1.06 × 10^−09^ per site per generation for lineages IB and IC, respectively. While migration rates between subpopulations of lineage IC were lower than for lineage IB, estimates of 0.05–0.2 migrations per generation suggest extensive movement between populations. This study clearly shows that *A*. *flavus* populations are strongly structured by evolutionary lineage and that both recombination and migration rates are sufficiently high to limit population substructuring.

Populations of *A*. *flavus* have a clonal and recombining population structure [10,32,36,37,39,40], and frequently maintain a mix of aflatoxigenic and nonaflatoxigenic strains [21,37,41]. Olarte *et al* (2012) provided the first direct evidence that sexual recombination and chromosome crossover events in parents influence mycotoxin phenotypes of *A*. *flavus* progeny strains [32]. *A*. *flavus* is heterothallic and sexual reproduction occurs between isolates of opposite mating types, *MAT1-1* and *MAT1-2*, that belong to different vegetative compatibility groups (VCGs) [42,43]. Previous research [44] reported that the degree of fertility in *A*. *flavus* was strongly influenced by the parental origin of the sclerotia, whereby one combination of sclerotia-conidia was highly fertile and the reciprocal combination exhibited low fertility. Field experiments where single strain mature sclerotia were sprinkled on the soil surface and allowed to be fertilized from natural soil populations showed that the same sclerotium can be fertilized by multiple conidial parents and acquire novel alleles [44]. Because the fertility of the sclerotium appears to be driving sexual reproduction in field populations, any significant difference in sclerotium fertility between *A*. *flavus* lineages increases the potential for asymmetrical gene flow. Variation in fertility has been demonstrated in the laboratory for inter-lineage [32,43] and interspecific [33] crosses, but it is not known if the differences in fertility at the individual level are representative of overall lineage fertility which has the potential to structure natural populations.

In filamentous fungi, the vegetative compatibility system is a self/non-self recognition system controlled by a series of heterokaryon *(het)* incompatibility loci [45]. Heterokaryon incompatibility is the inability of two strains to undergo fusion of vegetative fungal cells. Twelve putative *het* loci have been identified in *A*. *flavus* and in most cases alleles must be identical at all *het* loci for stable hyphal fusions to occur [46]. Fungal individuals can be grouped into VCGs based on their multilocus genotypes, provided that marker loci used for genotyping are in linkage disequilibrium with *het* loci on the same chromosomal segment [46]. Several studies report the evolution of new VCGs in the same regions from year to year [47,48] and in progeny strains when compared to parental VCGs after a single generation of sex [32]. This is an important observation because each VCG is a clonal lineage comprising isolates that are predominantly the same mating type and have similar toxin profiles [23]. Therefore, increasing VCG diversity in field populations is expected to increase opportunities for sex and recombination in *A*. *flavus*, particularly in recombination hotspot regions such as the aflatoxin gene cluster, yielding potentially different toxin profiles and a range of aflatoxin concentrations [32,49].

Laboratory and field experiments indicate that a single round of sex can significantly increase genetic diversity [32,44]. Can this increase in genetic diversity be applied towards a long-term establishment of a non-aflatoxigenic population in the field? Carryover of an introduced biocontrol agent over seasons is possible. One example is persistence of the introduced strain over four years after initial application, along with reduced aflatoxin contamination in pistachio [50]. Other examples of carryover have been recently reviewed [51]. However current management practices recommend reapplication of biocontrol agents each growing season because of uncertainty in the quantity and effectiveness of the carryover population to reduce aflatoxin in the crops. Additionally, there is a need for more information on their long-term effect on the native population structure. The lack of predictive models for aflatoxin contamination and high cost of application deters growers in moderate to low-risk areas from applying the biocontrol products. Improving on biocontrol to make it more sustainable is attractive for economical and practical reasons; however, this requires better knowledge of the underlying genetic and environmental processes that influence biocontrol. This suggests that a better understanding of *A*. *flavus* population genetics over time and space is key to developing more sustainable and predictable approaches to manage mycotoxin contamination of crops. Here we adopt a population genomics approach to explore the effect of released biocontrol strains on native populations in cornfields, for up to three years after initial application.

## Materials and methods

### Sampling and DNA isolation

In 2013 and 2014, *A*. *flavus* was sampled from four cornfields in four states: Texas (Texas A&M University Farm, Burleson County, TX; 30.5472° -96.4297°), North Carolina (Upper Coastal Plain Research Station, Fountain Farm, Rocky Mount, Edgecombe County, NC; 35.8937° -77.6811°), Arkansas (Newport Research Station, Newport, Jackson County, AR; 35.5714° -91.2602°), and Indiana (Southeast-Purdue Agricultural Center, Jennings County, IN; 39.0334°, -85.5258°). Prior to our study, these corn fields had no history of biological control application. Fields were planted uniformly with corn and then the treatments were established within fields later in the season. There were three treatments: untreated control, Afla-Guard, and AF36. Each treatment was replicated four times using a complete, randomized block design. Each replicate consisted of two, 30-ft rows, which were separated from other experimental replicates by 100 feet. Best management practices for irrigation, weed management and fertilization followed local recommendations for each location. Corn hybrid selection also followed local recommendations. The selected corn hybrids used were different for each location but were commercially appropriate for the location based on climatic adaptation and yield potential. They were BT-traited and some were herbicide-tolerant, if required by the production area. Fields were maintained under a no-till, no crop rotation system. The two nonaflatoxigenic *A*. *flavus* strains were applied as commercial preparations, following the label, as would be done on a commercial farm. Specifically, either sterilized wheat kernels (AF36) or dehulled barley (Afla-Guard) was the carrier/nutrient source for dormant *A*. *flavus* propagules. These materials were scattered over the top of the rows at tasseling (VT growth stage) at an equivalent rate of 20 lb/acre.

For each plot, *A*. *flavus* isolations were made from 20 pooled, 10-cc sub-samples of soil collected from between plants within each of the two rows of the plot, sampling approximately the whole row length. Soil samples were collected from each plot at three different times during the season: before biocontrol treatment (pre-application), three months after biocontrol application (post 3-months), and one year after application (post 1-year). *Aspergillus flavus* was isolated from soil samples by dilution plating onto modified dichloran rose Bengal agar, as described previously [52]. At crop maturity, eight ears (one per plant) were sampled from both rows of a plot, along the row length of each plot, shelled separately, and 100 kernel sub-samples from each ear were surface sterilized in 10% bleach, followed by two rinses in sterile water. The 100 kernel samples were evenly spaced onto moistened sterile paper towels in 20 cm × 20 cm sterile aluminum pans. The pans were placed in Ziploc bags and incubated at ambient room temperature for one week. Colonies of *A*. *flavus* sporulating on kernels were subcultured onto Czapek-Dox agar.

To examine more long-term changes in population genetic structure after initial biocontrol application we focused on four commercial dryland cornfields in Burleson County, Texas. Fields A (Ships clay, 30.5487° -96.4275°) and C (Weswood silt loam, 30.5398° -96.4165°) had never received a biocontrol treatment, while fields B (Weswood silty clay loam, 30.5545° -96.4243°) and D (Weswood silt loam, 30.5392° -96.4159°) were treated with 20 lb/acre of Afla-Guard in May 2011. Since 2011, fields A, C, and D were continually planted with corn, while field B was rotated with wheat in 2013 prior to planting with corn in 2014. Because field assays consistently showed aflatoxin levels well below 100 ppb over several years, we conducted population genomic analyses from soil and kernels to better understand how the underlying lineage composition and population genetic structure contribute to overall lower aflatoxin levels.

DNA was isolated from *A*. *flavus* spores derived from pure cultures using DNeasy UltraClean® Microbial Kit (Qiagen) with the following modifications. When resuspending the fungal cells in PowerBead Solution, RNase A was added to degrade any RNA present. Spores were vortexed using the MOBIO Vortex Adapter and Disrupter Genie, incubated at 65°C, and vortexed again to ensure that spores were fully ruptured. Finally, DNA was eluted in PCR-quality water for genotyping by sequencing.

### Genotyping by sequencing

We used double digest Restriction Site-Associated DNA Sequencing (ddRADseq) to identify genome-wide Single Nucleotide Polymorphisms (SNPs) [53]. These dense SNP markers allow us to unambiguously track the applied *A*. *flavus* NRRL 21882 and NRRL 18543 strains that are the active ingredients in the Afla-Guard and AF36 biocontrol products, respectively. To determine optimal restriction enzyme combinations for ddRADseq we performed *in silico* digestions of *A*. *flavus* NRRL 3357 [54] and *A*. *oryzae* RIB40 [55] reference genomes. *Aspergillus oryzae* is a domesticated close relative of *A*. *flavus* that is used in the food fermentation industry. To determine the best enzyme pairs for genotyping by sequencing, twelve restriction enzymes (ClaI, BamHI, BglII, DraI, EcoRI, EcoRV, HindIII, PstI, SalI, SmaI, XmaI, MspI) were used in all paired combinations to generate fragment pools for six different size ranges (150–250 bp, 250–350 bp, 350–450 bp, 450–550 bp, 550–650 bp, 650–750 bp). We targeted fragment pools that yielded approximately 5,000 fragments. This number would maximize the number of strains that could be multiplexed on an Illumina NextSeq machine (400M paired end reads) and achieve at least 20X read coverage per individual.

Total genomic DNA was isolated and quantified using Quant-iT™ PicoGreen® dsDNA Assay Kit (Invitrogen). Two restriction enzymes, MluCI (= Tsp509I) and MspI, were identified that would allow us to multiplex up to 1,152 strains and sequence 5,000 fragments/individual to a depth of at least 20X. DNA (200 ng) was digested with both enzymes; a universal adapter was ligated to the overhangs produced by MluCI and one of 48 barcoded adapters was ligated to overhangs produced by MspI. A second quality check with Quant-iT™ PicoGreen® dsDNA Assay Kit was performed to ensure DNA was digested. Size selection was done using a Pippin Prep (Sage Science) to keep fragments in the range of 450–550 bp. This is the total size that includes the targeted 350–450 bp fragment pool plus the ligated universal and barcode adapters. A universal primer was annealed to the barcoded adapter and one of 24 indexed primers was annealed to the universal adapter followed by PCR amplification using KAPA HiFi Hotstart Readymix (Kapa BioSystems). This design ensured that only DNA sequences with both ligated adapters will be sequenced. All strains in each sublibrary were quantified using Bioanalyzer (Agilent) and then pooled in equimolar ratios. Sublibraries were quantified and a final pooling was performed before paired-end sequencing. The unique combination of indexed primer and barcoded adapter allowed for demultiplexing first by Illumina index and then by barcode. The DNA for all isolates were pooled and sequenced using 150 bp paired end reads on the Illumina NextSeq® platform (NC State University Genomic Sciences Laboratory).

### Data processing for variant discovery

All ddRADseq data were analyzed using workflows implemented in a locally deployed instance of Galaxy [56] at NC State University and the Mobyle SNAP Workbench [57]. Briefly, the process_radtags script from the Stacks package (http://catchenlab.life.illinois.edu/stacks/) was used to demultiplex 48 barcodes for each Illumina NextSeq sublibrary. Trimmomatic [58] was used to trim low quality bases from the end of reads and crop bases from the ends regardless of quality. Filtered read pairs were aligned to the *A*. *oryzae* RIB40 reference genome using the MEM algorithm in BWA [59]. Sequence alignment (SAM) files generated from BWA-MEM were converted to Binary (BAM) files and sorted by coordinate order using SortSam in Picard tools (https://broadinstitute.github.io/picard/). PCR duplicates were removed using Picard MarkDuplicates and read groups were added for each strain using Picard AddOrReplaceReadGroups. The strains were then genotyped using the HaplotypeCaller variant discovery pipeline in GATK v3.5–2 [60]. GATK variant calling is designed to maximize sensitivity, so there could be many false positives. Subsequent filtering of variants eliminated false positives and negatives and was performed according to GATK Best Practices recommendations [61,62]. Variant Call Format (VCF) files from GATK were subjected to various levels of filtering using VCFtools [61] and variant calls were visualized in JBrowse [63]. Visualization of aligned reads in JBrowse for both reference and sampled strains was performed for unequivocal assignment of strains to full, partial, and missing aflatoxin gene cluster categories. We further confirmed this scoring method using two independent approaches. First, we downloaded and aligned to the *A*. *oryzae* RIB40 reference, as described above, a total of 92 *A*. *flavus* L genomes examined in a previous study [37], which were characterized as full (*n* = 65), partial (*n* = 8) and missing (*n* = 19) clusters. Second, three representative isolates in our study for each of the full, partial, and missing aflatoxin cluster categories were subjected to PCR amplification of cluster genes to confirm JBrowse cluster configurations, as described previously [10].

### Genome-wide haplotype analysis

We inferred genome-wide haplotypes (GWHs) for 815 *A*. *flavus* isolates using MeShClust [64] implemented in the DeCIFR toolkit (https://tools.decifr.hpc.ncsu.edu/meshclust). MeShClust uses a mean shift algorithm to cluster DNA sequence data, which performs better than other clustering methods when the exact sequence similarity threshold is not known [64]. The optimal sequence similarity threshold in MeShClust was determined as the number that correctly clustered a reference panel of *A*. *flavus* VCGs. This was also aided with an additional parameter, the delta number, that determines how many clusters are examined in the final clustering stage. It is expected that isolates within a VCG are very similar to each other and should cluster more tightly than with isolates from different VCGs. Using the VCG panel to guide our clustering, we also explored a more conservative approach of collapsing strains into haplotypes by excluding all sites with unknown base calls using SNAP Map [65]. For the larger data set of 815 isolates, it is expected that this approach would eliminate many SNPs and group isolates from different VCGs together. The distributions of GWHs were analyzed in JMP Pro 14 (SAS Institute Inc., Cary, NC, 1989–2020) using the “Summary” and “Distribution” functions. To observe trends in their distribution, the GWHs were grouped by state (TX, NC, AR, IN), substrate (kernel, soil), treatment (untreated, Afla-Guard, AF36) and sampling period (pre-application, post 3-months, post 1-year, post 3-years). Comparisons of GWHs unique and/or in common among the conditions were analyzed using Venny 2.1 (https://bioinfogp.cnb.csic.es/tools/venny/).

### Evolutionary lineage assignment

Assignment of *A*. *flavus* isolates to evolutionary lineage was based on clustering of reference isolates of known lineage with sampled strains using four complementary methodologies: 1) network inference where nodes are grouped into clusters using a distance-based method, 2) bootstrap support values in maximum likelihood phylogenetic reconstruction where a lineage is defined as a monophyletic group with ≥70% bootstrap support, 3) an admixture model where each isolate has some fraction of the genome derived from *k* populations, and 4) a principal component method to estimate the number of distinct *k* clusters. Because inter-lineage recombination can confound lineage boundaries, to further confirm lineage assignment we expanded our sampling of *A*. *flavus* isolates by including 94 genomes that were examined in a previous study [37]. Importantly, the 94 isolates were sampled from three of the four geographical regions (Texas, North Carolina, and Indiana) examined in our study. This integration is useful for several reasons. First, it is not clear if using a different target species reference genome can bias variant calls and affect downstream analyses. Drott and coworkers used *A*. *flavus* NRRL 3357 which they sequenced using short and long-read technologies whereas we used the published *A*. *oryzae* RIB40 as our reference genome [55]. Both are chromosome-level assemblies and closely related species based on comparative genomics analysis [66,67]. In this case, read mapping and subsequent variant identification are not expected to be different [68] especially in haploid organisms [69] but should be explored whenever a nontarget reference genome is used.

To examine the influence of reference genome on variant calls, we applied our *A*. *oryzae* RIB40 reference-guided variant discovery pipeline described above to the 94 *A*. *flavus* paired-end sequence libraries downloaded from GenBank SRA (SRR12001133- SRR12001227). Four separate analyses were performed. First, the VCF for the 94 isolates was filtered to include only SNPs for direct comparison with published SNP number that was obtained using the *A*. *flavus* NRRL 3357 reference genome. Second, the VCF was filtered to include only the SNPs found in the DNA fragments examined in the present ddRADseq study. Third, the VCF file including only the 92 *A*. *flavus* L isolates (i.e., excluding the two S strains) was merged with the VCF for the current sample of 815 strains using VCFtools [61] to generate a combined VCF file whereby SNPs were called across all 907 isolates. Fourth, to examine whether the five loci (*aflM*, *aflW*, *mfs*, *trpC*, and *amdS*) that were used in a previous large-scale population genetic study [36] could identify the IB and IC lineages [35], we extracted the SNPs for these loci from the VCF file for the 94 isolates. For all four filtering scenarios, phylogenetic networks were inferred and compared to each other to identify shared clusters of interconnected nodes in the splits tree graph, as described below.

### Network and phylogenetic inference

The possibility of released biocontrol agents recombining with native strains was first examined using phylogenetic networks. The recombination history of the total sample of 815 isolates was reconstructed using the Neighbor-net algorithm implemented in SplitsTree4 v.4.14.8 [70]. The splits graph was examined for the presence of distinct evolutionary lineages (i.e., splits) and reticulate evolution (i.e., netted regions) which are indicative of hybridization between the lineages. Maximum likelihood (ML) phylogenetic analysis was performed using the program Randomized Axelerated Maximum Likelihood or RAxML version 8 [71] via the Cyberinfrastructure for Phylogenetic Research (CIPRES) Representational State Transfer Application Program Interface (REST API) [72] implemented in the DeCIFR toolkit (https://tools.decifr.hpc.ncsu.edu/denovo). The best-scoring ML majority rule consensus tree was based on 1,000 rapid bootstrap searches in RAxML using a GTRGAMMA model of rate heterogeneity with empirical base frequencies. Trees were visualized using the upload tree option of the Tree-Based Alignment Selector (T-BAS, version 2.3) toolkit (https://tbas.hpc.ncsu.edu/) [73,74].

### Population structure

The degree of genetic admixture and the optimal number of *k* clusters was determined using ParallelStructure [75], an R-based implementation of STRUCTURE version 2.3.4 [76,77] accessible via the CIPRES REST API [72]. Structure-formatted files were generated from genome-wide SNPs using SNAP Map [65]. The admixture model implemented in STRUCTURE was used to assign individuals to *k* clusters. Estimates of allele frequencies and membership probabilities of individuals in subpopulations were based on a Markov Chain Monte Carlo (MCMC) strategy of 100,000 sampling iterations after a burn-in period of 50,000 iterations; three independent simulations for possible *k* values ranging from 1 to 10 were performed for each subpopulation. To determine the optimal *k*, probability distributions were examined using *LnP(D)* and delta *K* methods [78] implemented in Structure Harvester v0.6.93 [79]. The estimated cluster membership coefficient matrices were examined in CLUMPP v1.1.2 [80] to determine the optimal number of *k* clusters across multiple runs. The individual cluster membership results from STRUCTURE were visualized using histograms in outer rings surrounding the total combined chromosomal ML phylogeny, as implemented in the DeCIFR toolkit (https://tools.decifr.hpc.ncsu.edu/structure).

Population structure was also examined using principal component analysis (PCA) implemented in EIGENSOFT SmartPCA v18140 [81]. A recent study has shown that PCA can yield highly biased results and should not be used as a first hypothesis generator in population genetic analyses [82]. Here, PCA was used to corroborate *k* cluster estimation that was based primarily on phylogenetic reconstruction methods assuming bifurcation or reticulation, and admixture models to characterize ancestral genetic structure. PCA uses a variance–covariance matrix to reduce the dimensionality of the original variables into a smaller number of new variables called principal components [83]. The plotted principal components (eigenvectors) provide different axes of variation that explain a fraction of the variance in the original data. The first eigenvector or PC1 considers the most variation possible with subsequent eigenvectors, PC2, PC3, etc., having less variation. Principal components were normalized to sum to 1 to reveal which eigenvectors explained more than half of the genetic variation, and the number of significant axes of variation was determined using the Tracy–Widom statistic [84]. The number of distinct *k* clusters was determined using the Gap Statistic [85], which is an unbiased estimate of the number of distinct clusters based on the top PCs with the largest eigenvalues that accounted for at least 50% of the explained variance. Significant PC’s and *k* clusters were displayed in two and three-dimensional graphs using the scatterplot3d package in R [86].

Tests of association of *k* cluster with lineage, sampling location, treatment, and substrate were performed using Fisher’s exact test, implemented in R [87]. Lineage-specific population parameter estimates of mean mutation rate (*θ*), recombination rate (gamma), and pairwise nucleotide diversity (*π*) in each state were calculated by averaging across all chromosomes in untreated and treated samples using the program SITES version 1.1 [88]. The ratio of mutation rate to recombination rate (u/c) was calculated for untreated and treated samples across each state and sampling period. Any significant deviation of the mutation frequency distribution from neutrality could point to population growth or selection and was tested using Tajima’s *D* [89], Fu and Li’s *D* and Fu and Li’s D* [90]. Neutrality tests were performed separately for each lineage. The pairwise fixation index (*F*_ST_) implemented in SITES was used to evaluate genetic differentiation among treatments and lineages.

### Population parameter estimation

We estimated population migration rate parameters, population splitting times and effective population sizes for *A*. *flavus* lineages IB and IC in the untreated and treated samples for populations in TX, NC, AR, and IN, and the TX commercial fields. Estimates of effective population sizes (*N*_*e*_), population migration rates (*N*_*e*_*m*; haploid migration rate) and population splitting times in years were based on the Isolation-with-Migration (IM) model assuming constant population sizes and migration rates, as implemented in the IMa3 and IMfig programs [91]. Prior to running IM simulations, variation across each of eight *A*. *flavus* chromosomes was subjected to four-gamete filtering to retain the largest nonrecombining partitions. While focusing on an IM model and recombination-free datasets lowers the statistical power for detecting migration rates that are greater than zero, the alternative approach of ignoring recombination and assuming a finite sites model results in disjunct posterior probability distributions and should be avoided [92]. The IMgc program [93] was used to obtain recombination-free chromosomal partitions using a weight ratio of 10 to 1 for individual strains and sites, respectively. This ensured that the largest number of strains and variable sites across each chromosome were retained for population parameter estimation.

Given the possibility that lineages IB and IC may have undergone genetic exchange with other *A*. *flavus* lineages (e.g., lineage IA) and closely related species in *Aspergillus* section *Flavi*, estimates of demographic parameters were based on an IM model of two populations (lineages IB and IC) and a potential third unsampled ghost population that served as an outgroup population. A mutation rate of 4.2 × 10^−11^ per base per generation in *A*. *flavus* [94] and a generation time of 0.17 years [95] were used to obtain demographically scaled estimates of population parameters. Prior values for effective population sizes, migration rates, and divergence times were based on the geometric mean of Watterson’s estimate of *θ* across all eight chromosomes, calculated separately for lineages IB and IC in each state and sampling period. The upper bound for the prior on the population size parameter was set to five times the largest value of these geometric means across lineages IB and IC; the upper bound for the prior migration rates was set to five times the inverse of the geometric mean of *θ;* and the upper bound for the prior on splitting times was set to two times the geometric mean of *θ*, according to the IMa3 documentation [96]. These are considered ballpark estimates and were used as a guide for selecting a set of priors that would work across all populations.

Several preliminary short runs of a few chains were performed to select the best heating schemes that maintained high swap rates (between 0.7 and 1) between adjacent pairs of chains. Good mixing in longer runs was based on high swapping rates between successive chains, non-zero values of autocorrelations between parameter estimates across sampling iterations, and high effective sample sizes (ESS > 10,000). To ensure sufficient mixing, IMa3 runs were done with 256 heated chains with a geometric heating model (0.99 and 0.65 for the first and second heating parameter, respectively), a burn-in of 1,000,000 steps prior to sampling, and 50,000 sampled genealogies per chromosome. The final MCMC mode runs were repeated at least twice with a different random number seed to ensure convergence of parameter distributions. Migration arrows drawn using the IMfig program denoted population migration rates that were statistically significant [97]. All runs were performed through the REST API service at CIPRES [72] using program calls from the IMgc (https://tools.decifr.hpc.ncsu.edu/imgc) and IMa3 (https://tools.decifr.hpc.ncsu.edu/ima3) tools implemented in the DeCIFR toolkit.

### Mating type distribution

Mating types were scored using diagnostic ddRADseq fragments located within the *MAT1-1* idiomorph spanning positions 1,581,022–1,581,132 on chromosome 6 of the *A*. *oryzae* reference genome. A 1:1 distribution of *MAT1-1* and *MAT1-2* is indicative of populations undergoing sexual reproduction and this was tested using a two-tailed binomial test implemented in MS Excel. The test was also performed on clone-corrected haplotypes to correct for skewness in mating type distributions due to clonal amplification. Clone-correction was performed by counting the total number of unique GWHs in each *MAT1-1* and *MAT1-2* category; individuals or GWHs containing both mating types were counted twice as a *MAT1-1* and a *MAT1-2*. A representative sample of 47 isolates from each *MAT* idiomorph were selected for PCR validation using *MAT1-1* and *MAT1-2* specific primers, as reported previously [42]. In the presence of strong population structure, mating type distributions were examined separately for each distinct genetic cluster.

### Phylogenetic incongruence and recombination

Since populations of *A*. *flavus* are reported to have both a clonal and recombining population structure [39], topological concordance across chromosome and mitochondrial phylogenies was used to further examine the contributions of clonality and recombination in the evolution of lineages, GWHs, and individuals. This phylogenetic method implemented in the DeCIFR toolkit (https://tools.decifr.hpc.ncsu.edu/trees2hypha) uses the Hypha package module of Mesquite v3.51 [98,99] to display the clonal and recombinant history of each ancestral node and all of its descendant strains. Specifically, Hypha was used to compare the internodal support values harvested from each chromosomal phylogeny on the total evidence tree inferred from a concatenated genome-wide SNP matrix. Nodal grid support values were based on a bootstrap threshold support value of 70% and were output as node annotations on the total evidence display tree. Support values were visualized for each chromosome phylogeny using grids on branches of the display tree with colors showing node bipartitions that were supported at a bootstrap support value ≥70% (black node) or <70% (white node); if the specific node bipartition was not found in the display tree this was reported as missing or inapplicable (grey node). Grids that were filled in with mostly black squares indicated that the descendants of that node were predominantly clonal. High conflict (red node) was used to indicate a node bipartition in the chromosome tree that conflicted with the displayed tree at a bootstrap support value ≥70% most likely due to recent recombination (i.e., independent assortment and crossovers) among strains in descendant branches at terminal nodes or ancestral recombination at internal nodes. Low conflict (cyan node) was used for nodes that were not recovered by the bootstrap analysis because there was either insufficient variation or too much confounding variation (i.e., homoplasy) due to recombination among strains in descendant branches. We would expect node bipartitions that comprise strains that belong to the same GWH to be congruent across all chromosomes. If GWHs associate closely with VCGs they can be used as a proxy for VCGs. In the case of reference strains that are known *a priori* to belong to the same VCG and are of the same mating type, high conflict at one or more chromosomes provides evidence of recombination in the immediate common ancestor.

### Chromosomal linkage disequilibrium

Chromosome-wide linkage disequilibrium (LD) between SNP markers distributed across each chromosome was performed using Haploview 4.2 [100]. Both *r*^*2*^ and *D’* pairwise LD measures were calculated between adjacent SNP markers in all populations for each sampling period: pre-application, 3-months, 1-year and 3-years post-treatment. Intra-chromosomal LD blocks were estimated using the Solid Spine (SS) method of Haploview using the default parameters and a missingCutoff of 0.8. The SS algorithm identifies an LD block if the first and last markers in a block are in strong LD with all intermediate markers, but those intermediate markers can be in weak LD or no LD with each other. Haploview outlines the edge of the spine of strong LD in a triangular matrix of pairwise LD statistics. The coloring scheme is based on the values of *D’* and LOD (logarithm of the likelihood-odds ratio) where bright red represents strong LD (LOD ≥ 2, *D’* = 1), shades of pink/red represent intermediate LD (LOD ≥ 2, *D’* < 1), blue represents weak LD (LOD < 2, *D’* = 1) and white represents no LD (LOD < 2, *D’* < 1). The strength of LD was determined by averaging *r*^*2*^ for each lineage, chromosome, and sampling period. We expect the release of biocontrol strains to initially increase the clonal component of population in the short-term and result in higher mean *r*^*2*^ values when compared to mean *r*^*2*^ values in pre-application fields. In the more long-term, the released biocontrol strains are expected to increase the opportunities for both intra- and inter-lineage recombination and result in lower mean *r*^*2*^ values than in pre-application fields.

### Aflatoxin quantitation and analysis

For each state (TX, NC, AR, and IN) and sampling period (pre-application, post 3-months, post 1-year, and post 3-years) at least one strain from each lineage (IB and IC), treatment (untreated, Afla-Guard, and AF36), substrate (soil and kernel), and aflatoxin cluster configurations (missing, partial, and full) was selected when available for aflatoxin B_1_ quantitation. Aflatoxin was quantified using a method which allows detection of aflatoxin B_1_ production from fungal mycelia in liquid culture [101]. Reference strains were included for comparison with previous quantification methods [49,52,102]. Briefly, isolates were grown on Potato Dextrose Agar (PDA) plates at 30°C for a total of 7 days: 5 days in the dark and 2 days in light. Glass vials with 7 mL of YES media [103] were inoculated with a loop-full of conidia for each isolate, with three replicates grown per isolate. Liquid cultures were incubated in the light at 30°C for 7 days. For each culture, a 1 mL aliquot of media was transferred to a new vial, 1 mL of chloroform was added, and samples were vortexed and then allowed to separate at rest. A total of 500 μL of the chloroform layer was transferred to a clean vial and evaporated under nitrogen stream. Dried aflatoxin samples were resuspended in 1 mL of methanol for analysis and purified by passing through 1 mL polypropylene SPE tubes containing a 200 μL tube full of alumina basic.

Aflatoxins were quantified by HPLC on two different instruments: (1) HPLC with fluorescence detection at the Biomanufacturing Training and Education Center’s Bioprocess and Analytical Services at North Carolina State University, and (2) HPLC with mass spectrometry detection at our laboratory at North Carolina State University. The detection by HPLC-fluorescence (excitation 365 nm, emission 455 nm) followed a previously published protocol [104]. Serial dilutions were used to determine the limit of aflatoxin B_1_ detection at 0.0020 μg/mL and quantification at 0.0039 μg/mL [101].

HPLC with mass spectrometry detection was done on a quadrupole LC-MS (Ultimate 3000 UPLC/ISQ EC, Thermo Fisher Scientific) in Single Ion Monitoring (SIM) mode. The instrument was also equipped with UV-vis Diode Array and Charged Aerosol Detectors. The chromatographic conditions were based on a recent mass spectrometry-based method for analysis of mycotoxins in cannabis matrices [105]. Separations were performed on an ACE Excel 1.7 μm C18-PFP column (100 x 3.0 mm) in a thermostated column compartment at 45°C. The mobile phase consisted of a gradient of solutions A and B (Solution A: water + 1 mM ammonium formate + 0.1% formic acid; Solution B: methanol + 1 mM ammonium formate + 0.1% formic acid). During the 9-minute gradient the proportion of A and B solutions was programmed as follows: 0–1 min constant at 95% A / 5% B; 1–2 min linear gradient to 55% A / 45% B; 2–8 min linear gradient to 10% A / 90% B; 8–9 min: constant at 10% A / 90% B. The column was re-equilibrated for 2.5 minutes at 95% A / 5% B before the next injection (injection volume = 50 μL) and the flow rate was constant at 0.8 mL/min. The mass spectrometer detector was set to positive mode Single Ion Monitoring (SIM) targeting Aflatoxin B_1_ (*m/z* = 313.1), B_2_ (*m/z* = 315.1), G_1_ (*m/z* = 329.1), and G_2_ (*m/z* = 331.1) and positive mode scans were acquired during the same run. The peaks for aflatoxin B_1_ (retention time = 5.87 min) and B_2_ (retention time = 5.61 min) were baseline-separated with a low background intensity. The detection limit determined by repeated injection of standards and samples was ~0.3 ng/mL (~1 nmol/L) in the injected sample or ~1 ng/mL (~3 nmol/L) in the original undiluted sample extracts. Quantification with aflatoxin B_1_ and B_2_ standards (Sigma-Aldrich A6636-5MG) was based on a six-point calibration curve in the range of 0.001–0.5 μg/mL. Standards were analyzed at the beginning and end of every analysis batch, and two blank injections were included between samples of different cultures. The extracted samples in methanol were diluted with A and B solutions to 70% aqueous / 30% organic to achieve symmetric chromatographic peak shapes. The dilution was adjusted so that injected samples contained < 0.5 μg/mL (< 1.6 μmol/L) of aflatoxin B_1_ to remain within the linear range and minimize carry over. The cultures produced primarily aflatoxin B_1_ and concentrations before dilution were estimated using a fluorescence plate reader (BioTek Synergy HTX, excitation: 360 nm, emission 460 nm).

To determine if there was quantitative variation in B_1_ aflatoxin concentrations between lineages IB and IC, aflatoxin frequency distribution plots and cumulative distribution functions were graphically portrayed for each lineage. Mean toxin concentrations were determined and significant differences in toxin distributions between lineages IB and IC were tested by performing Kolmogorov-Smirnov tests, as implemented in Matlab (MathWorks Inc., Natick, MA, USA).

## Results

### Sampling and variant discovery

A total sample of 815 isolates was examined across all states, treatments, and sampling periods (Tables 1 and S1). Also included was a panel of 26 reference strains for which mating type [42], toxin profile [102], VCGs [52], multilocus haplotypes and evolutionary lineage [10] were known and used to cross-validate genotyping by sequencing results (S2 Table). A total of 672,302 unfiltered variants were discovered and 6,806 biallelic variants were retained after filtering (Table 2). The filtering was performed using a minimum and maximum value of the minor allele frequency set to 0.001 and 0.5, respectively, to ensure that rare sequence variants were kept for genotyping individual biocontrol strains with high resolution. Moreover, because phylogenetic and network inference, phylogenetic incongruence, and LD analysis rely on informative SNPs that group together two or more individuals, filtering included a genotype call rate of 90% across all individuals.

**Table 1:** Population Samples of *A*. *flavus*^1^ for different sampling periods.

State	Pre-application	Post 3-months	Post 1-year	Post 3-years^2^
**TX**	40	20	153	161
**NC**	39	20	141	NA
**AR**	39	20	55	NA
**IN**	30	9	62	NA
**Total**	148	69	411	161

^1^There were an additional 26 reference strains that were examined using ddRADseq for a total sample size of 815 (see S1 Table).

^2^The samples collected for Post 3-years were all from TX (NA = not applicable).

**Table 2:** Number of variable sites for phylogenetic and population genomic analyses.

Sampling period	Sample size^1^	Unfiltered	Filtered^2^	SNPs^3^
**Pre-application**	172	451,283	27,529	610
**Post 3-months**	93	451,283	27,529	592
**Post 1-year**	436	447,324	21,260	713
**Post 3-years**	187	271,813	14,677	1,282
**TOTAL**	815	672,302	6,806	5,750^4^

^1^Number including reference strains.

^2^Number of sites in character matrix for phylogenomic and network inference.

^3^Number of SNPs (excluding ambiguous sites) for STRUCTURE and PCA analysis.

^4^Number of SNPs (ambiguous sites included) in the total sample for clone-correction and population parameter estimation.

Visualization of a total of 815 *A*. *flavus* reads aligned to the *A*. *oryzae* reference genome clearly distinguished strains as having full (*n* = 522), partial (*n* = 19), or missing (*n* = 274) aflatoxin gene clusters (S1 Table). All partial cluster strains were similar to deletion pattern C [21] missing 12 genes in the aflatoxin cluster but with the distal telomeric region of the right arm of chromosome 3 intact, as observed in reference isolate IC311 (S1A–S1C Fig). This approach worked provided that ddRADseq yielded sufficient coverage across the genome so that deletions were obvious to score in JBrowse. To ensure this, we only examined strains that had >20X read coverage. This level of coverage also allowed us to be more stringent in SNP identification by enforcing a minimum genotype call rate of 90%. We validated our approach by examining 92 *A*. *flavus* isolates from a previous study that were also characterized for deletions in the aflatoxin cluster [37]. Two out of nine reported partial cluster isolates from the study by Drott and coworkers were similar to deletion pattern G and the remaining six isolates were similar to deletion pattern C (S1D Fig); all missing cluster strains were also correctly identified using JBrowse. It should be noted that the alignments visualized in JBrowse provided more resolution of sequence breakpoints for the partial cluster strains (D70 and D79) and missing cluster strain (D23) which were not reported in the paper [37]. Results from PCR amplifications of cluster genes of representative isolates from each cluster category in the present study corroborated the scoring of cluster deletion patterns using JBrowse.

### Genome-wide haplotype inference

Overall, a total of 353 unique genome-wide haplotypes (GWH H0-H352) were inferred among the 815 individual strains (S1 Table). This was based on clustering 6,806 variants using a sequence similarity threshold of 0.987 and a delta value of 5. The two biocontrol strains IC201 (= Afla-Guard; VCG 24) and IC1179 (= AF36; VCG YV36) belonged to haplotypes H1 and H115, respectively. Also included in H1 was IC253 which shares the same VCG 24 as IC201 (S2 Table). Other VCGs with multiple representatives were also clustered into unique haplotypes such as IC221 and IC222 (H40; VCG 4). The 0.987 clustering threshold separated reference strains that belong to different VCGs into distinct haplotypes: IC277 (H29; VCG 32), IC278 (H338; VCG 33), IC301 (H333; VCG 56), IC307 (H341; VCG 62), IC308 (H336; VCG 63), and IC313 (H271; VCG 76). Two VCGs with multiple strains: VCG 1 (IC217, IC218) and VCG 5 (IC225, IC226) were clustered into haplotype H21. During the clustering processes MeShClust generated a semi-ordered list of clusters whereby adjacent clusters (i.e., GWH numbers) have more similar sequences. This was observed with H69 (IC220; VCG 2) and H70 (IC219; VCG 2), and with H329 (IC230; VCG 6) and H330 (IC229; VCG 6).

Because subsequent haplotype analyses required the exclusion of GA strains as VCG reference standards, there were 628 strains to analyze from the four remaining sampling locations and three sampling time points. The 3-year post-treatment analysis in TX included 161 strains and was conducted separately. Those 628 strains represented 276 unique genome-wide haplotypes (S3 Table). The haplotype frequency distribution is shown in S3 Table, where haplotypes H1 (28%) and H115 (3%), highlighted, in red bars denote the IC201 (Afla-Guard) and IC1179 (AF36) biocontrol haplotypes, respectively. The remaining 255 genome-wide haplotypes each had a single strain and a frequency of 0.16%, which accounted for 41% of the total sample.

When summarized by state (AR, IN, NC, TX), the only two haplotypes that were shared among all four states were the two biocontrol haplotypes H1 and H115 (S4 Table). The majority of GWHs were unique to individual states: TX (*n* = 70); NC (*n* = 95); AR (*n* = 65); and IN (*n* = 36)). The remaining eight haplotypes were shared between two or three different states. Overall, only five haplotypes (H1, H140, H22, H229, H240) were shared across the three different sampling periods: pre-application, post 3-months, and post 1-year, and twelve haplotypes were in common between the pre-application and post 1-year (S5 Table). There was a 2.5-fold increase in the number of unique haplotypes across all states in post 1-year (*n* = 178) compared to the pre-application (*n* = 73); a similar fold increase was observed in NC and TX and to a lesser extent in AR and IN (S5 Table).

### Evolutionary lineage assignment

To identify the lineage composition of our sampled populations and to further explore evolutionary lineage boundaries, we leveraged the availability of 94 *A*. *flavus* genomes (92 *A*. *flavus* L and 2 *A*. *flavus* S strains) from a recent population genomics study [37]. We first examined whether the choice of reference genome impacted SNP discovery and downstream analysis (S2 Fig). A total of 817,774 SNPs were identified across the 94 *A*. *flavus* genomes using the *A*. *oryzae* RIB40 reference genome which was close to the 910,777 SNPs reported using the *A*. *flavus* NRRL 3357 reference genome [37]. If we examined only the ddRADseq fragments, then 5,870 SNPs were identified across the 94 *A*. *flavus* genomes. There was no significant difference in the grouping of isolates in the inferred neighbor-net networks for the total and reduced SNP datasets (S2 Fig). Merging the VCF file for the 92 *A*. *flavus* L isolates with the VCF file for the 815 isolates in the present study yielded 6,833 SNPs across 907 individuals. The inferred Neighbor-net network for the combined 907 *A*. *flavus* L isolates showed that the 92 isolates from the previous study nested within the boundaries of lineages IB and IC (S3A Fig). Importantly, isolates in populations A and C are within the range of variation observed in lineage IC and isolates belonging to population B are tightly clustered with representatives of lineage IB, which is also corroborated by PCA and cluster analysis using the gap statistic (S3B Fig). There is also some evidence of inter-lineage hybridization driving genetic differentiation and population subdivision in population C (see circles in S2A and S3A Figs).

We further corroborated lineage structure by filtering SNPs for the five loci (*aflM*, *aflW*, *mfs*, *trpC*, and *amdS*) across the 94 *A*. *flavus* isolates, which included the two S strains. The inferred Neighbor-net network for the 33 SNP data matrix (S4A Fig) showed two distinct groups that corresponded to lineages IB and IC in the larger network and a hybrid zone that included strains with completely missing clusters and representative isolates from lineage IC (S4B Fig). A closer examination of the sequence matrix for hybrid strains showed signatures of recombination between lineages IB and IC for the putative hybrids in the network (see D82 recombinant in S5 Fig). This differentiation was also supported in the larger network where the putative hybrids identified from multilocus sequence analysis across five loci were positioned in the hybrid zone between the two lineages (see circles in S2A and S3A Figs). Finally, the two putative S strains (D21, D55) were grouped within lineage IB. In previous work, *A*. *flavus* S_B_ isolates in lineage IA were shown to share a most recent common ancestor with *A*. *flavus* lineage IB [35,36].

### Network and phylogenetic inference

**Fig 1:**
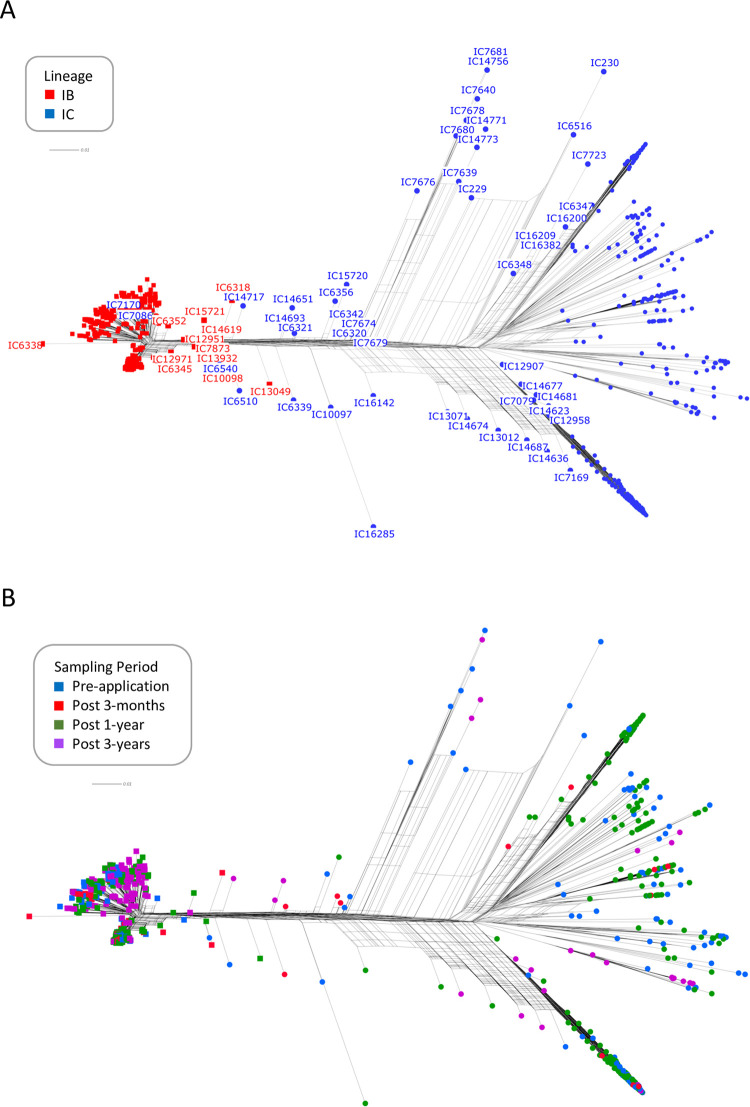
Patterns of reticulate evolution for 815 *A*. *flavus* isolates. **A.** Network splits tree graph highlighting in color membership of each isolate in lineage IB (red) or IC (blue) as determined from phylogenetic, admixture and cluster analysis. The terminal most splits include isolates that are representative of the clonal component of each lineage and isolates arising predominantly from intra-lineage recombination events. Putative hybrids resulting from recombination between lineages IB and IC are labeled and occupy mostly interior positions in the network. Putative hybrid strains are highly divergent and contain varying proportions of both lineages in their genetic background as shown in Fig 2. **B.** Overlay of sampling period on the network in **A** showing that inter-lineage recombination is a common feature of each sampling period. The network shows greater genetic heterogeneity within lineage IC compared to IB and that post 3-year samples were predominantly sampled from lineage IB. The branches in the network are drawn to scale and the scale bar represents 0.01 substitutions per site.

**Fig 2:**
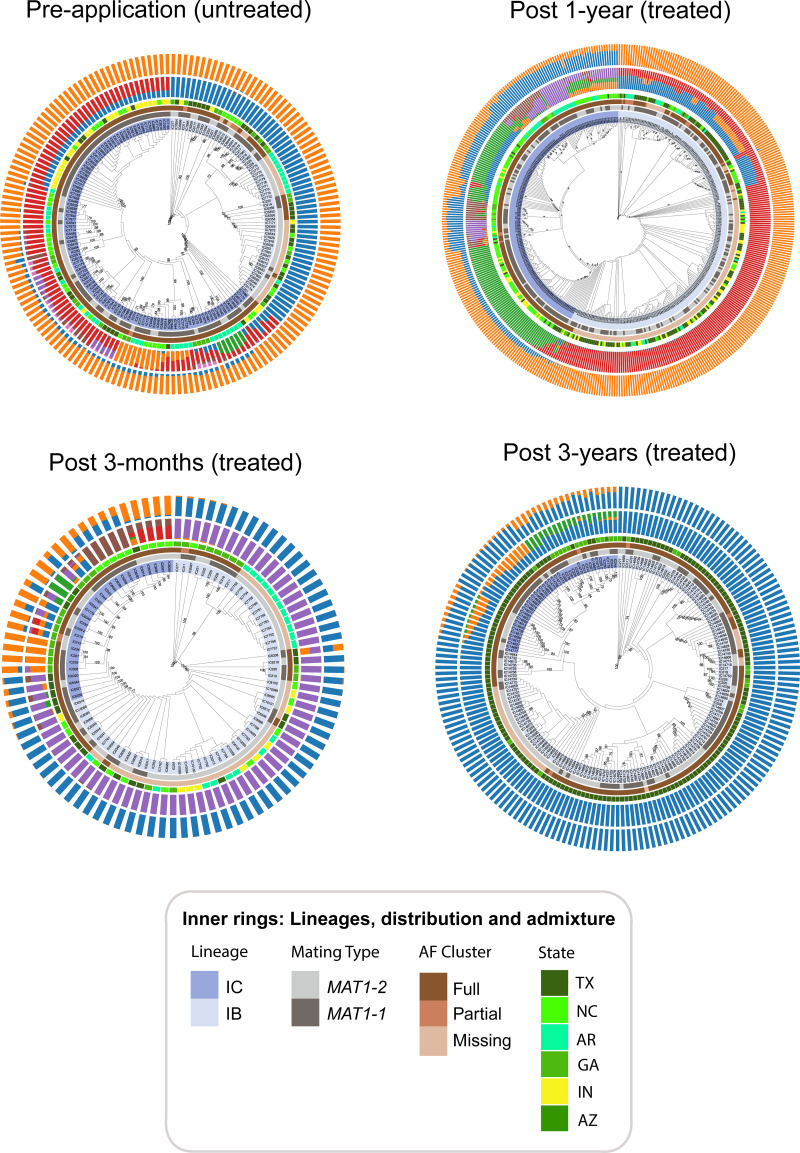
Phylogenetic and population structure analysis of *A*. *flavus* for each sampling period. The four innermost rings show *A*. *flavus* evolutionary lineage assignment (IB, IC), mating types (*MAT1-1*, *MAT1-2*), aflatoxin cluster configuration (full, partial, missing) and sampling locations for field experiments (TX, NC, AR, IN) including reference strains (GA, AZ). The phylogenies displayed in the center of the rings are the best maximum likelihood trees with branch support values displayed as percentages (zoom in to view). The results from admixture analysis using STRUCTURE are shown in the two outermost rings; the inner rings are the best clusters inferred from STRUCTURE LnP(D) and the outer rings are distinct clusters inferred using Evanno’s method.

Patterns of reticulate evolution for 815 *A*. *flavus* isolates are displayed in Fig 1. The splits tree graph separated two distinct groups of shared nodes with putative hybrids occupying interior positions in the graph. The sampled isolates and reference strains fell into two major clades that corresponded to lineages IB and IC and were highly supported (>95%) using bootstrap ML analysis and admixture modeling (Fig 2), and PCA with cluster inference using the gap statistic (S6 Fig). Fig 2 shows the best-scoring ML phylogenies inferred for the pre-application, post 3-months, post 1-year, and post 3-year sampling periods. Several consistent patterns were observed across all phylogenies. First, two distinct evolutionary lineages, IB and IC, based on sequence variation across eight *A*. *flavus* chromosomes were found in all sampled cornfields and treatments. Second, aflatoxin cluster structure (missing, partial, or full) corresponded closely with lineage membership (Fig 2; S6 Table). Across all three sampling periods (pre-treatment, post 3-months, post 1-year) strains in lineage IC had full clusters with the only exception observed for two partial cluster strains (IC13995, IC11274) that were sampled 1-year after biocontrol application. By comparison, lineage IB strains harbored full, partial, or missing aflatoxin clusters across the three sampling periods, but their frequency varied across states with TX having a greater proportion (65%) of full cluster strains in lineage IB compared to other states (S6 Table). Similarly, lineage IB predominated in TX post 3-year commercial fields and 80% of the isolates in the untreated and treated TX commercial fields had full aflatoxin gene clusters (Fig 2; S7 Table).

### Population structure

There were consistent and significant differences in the proportions of lineages IB and IC across sampling periods and between untreated and treated fields. For example, the post 1-year untreated and treated populations of *A*. *flavus* in TX were significantly (*P* < 0.05) skewed to lineage IB, whereas populations in NC and AR were significantly skewed (*P* < 0.05) to lineage IC (Table 3). Similar trends were observed in NC and AR cornfields prior to biocontrol application where *A*. *flavus* field populations were significantly (*P* < 0.01) skewed to lineage IC. The observed lineage skew to IB in the post 1-year TX cornfields was also observed in the post 3-year commercial TX fields. There was a significant association (*P* < 0.05) of lineage with treatment (Afla-Guard, AF36) in the post 3-months fields in TX, NC, AR, and IN, but no significant association (*P =* 0.13) of lineage with treatment (Afla-Guard, AF36, untreated) in the post 1-year across the four states. The network showed strikingly greater sequence variation within lineage IC compared to IB across each sampling period (Fig 1B). This was also supported by the per base pair estimates of the population mean mutation rate and nucleotide diversity showing a 5-fold higher value in IC compared to IB in the pre-treatment fields in AR, NC and TX (S8 Table). A similar trend of higher diversity in the IC lineage compared to IB was observed for treated fields post 3-months and for treated and untreated fields, sampled one year and three years after biocontrol application (S8 Table). The ratio of mutation rate to recombination rate (u/c) was highest for post 3-months treated lineage IB in AR (44.31) and lineage IC in NC (26.64) indicating strong clonality and LD initially after biocontrol application. In post 1-year treated fields the u/c ratio was higher in TX (1.32) than NC (1.18) for lineage IB but higher in NC (1.70) than in TX (1.13) for lineage IC. This reversal is consistent with the asymmetries observed in lineage frequencies in NC and TX (Table 3) and diversity estimates (S8 Table).

**Table 3:** Distribution of *A*. *flavus* lineages across states, treatments, and years ^1^.

	Pre-application		Post 3-months		Post 1-year				Post 3-years			
	Untreated		Treated^2^		Untreated		Treated^2^		Untreated		Treated^3^	
State	IB	IC	IB	IC	IB	IC	IB	IC	IB	IC	IB	IC
**TX**	26(8)	14(10)	13(5)	7(6)	45(*14)	4(4)	93(**35)	11(10)	61(28)	23(22)	72(**24)	5(4)
**NC**	11(7)	28(**23)	11(4)	9(3)	16(7)	30(*21)	17(5)	78(**46)				
**AR**	11(4)	28(**26)	20(2)	0(0)	6(3)	6(6)	17(9)	26(**26)				
**IN**	3(3)	27(11)	9(2)	0(0)	2(2)	8(7)	40(11)	12(7)				

^1^Clone corrected number and significance for each lineage is shown in parentheses based on a two-tailed binomial test; 0.01 < *P < 0.05 and **P < 0.01. Significance for the uncorrected samples is not shown.

^2^Plots were treated with either Afla-Guard or AF36 biocontrol products.

^3^Plots were only treated with Afla-Guard biocontrol.

STRUCTURE admixture analysis of the pre-treatment populations determined a best value of *k* = 2 clusters using the Evanno method and *k* = 7 clusters based on STRUCTURE LnP(D) (S9 Table). The two outermost rings in Fig 2 show the ancestral composition of each isolate for the four different sampling periods using the Evanno (inner ring) and LnP(D) (outer ring) cluster inference method. In the pre-treatment populations, lineage IB has a single dominant ancestry (i.e., blue cluster) whereas isolates in IC are a mix of several populations with one predominant genetic ancestry (i.e., red cluster). Most of the isolates in lineage IC have a mixed ancestry, with the greatest heterogeneity observed for isolates with bootstrap support values >70% in terminal clades. Clonal lineages show identical genetic backgrounds, for example, the largest clonal lineage in IC includes isolates that have the same proportion of two different ancestries (i.e., blue and red clusters). The shared genetic ancestry of the blue cluster is evidence of admixture between lineages IB and IC in the pre-treatment fields. In contrast to lineage IB which shows a single genetic background, the highly heterogenous genetic background for many strains in lineage IC indicates extensive genetic admixture and a large, structured population.

Evanno and Structure LnP(D) yielded best cluster estimates of *k* = 2 and *k* = 6, respectively, for populations sampled post 3-months (S10 Table) and post 1-year (S11 Table). Lineage admixture was also observed in *A*. *flavus* isolates sampled post 3-months. For example, based on the Evanno clustering method, IC6338 (a clear outlier in lineage IB in Fig 1) sampled from the AF36-treated plot in TX, contained 49% of the dominant genetic background in IB (i.e., blue cluster) and 51% of the dominant genetic background of IC (i.e., orange cluster) (Fig 2; S10 Table). Lineage ancestry was more difficult to discern in the post 1-year fields for *k* = 6 (inner ring; Fig 2; S11 Table). For example, IC11367 and IC15888 are from TX and NC, respectively, and contained approximately 34% of the dominant genetic background in IB (i.e., red cluster) and 65% of a different IC ancestry (i.e., blue cluster). The outer ring representing *k* = 2 in post 1-year indicates that isolates in lineage IC have a mixed ancestry with varying contributions from lineage IB (Fig 2). The TX commercial cornfields that were sampled 3 years after Afla-Guard was applied were significantly skewed (*P* < 0.01) to lineage IB (Table 3) and were predominantly of a single ancestry (Fig 2; S12 Table). There was mixing of lineages IB and IC, evident because strains in lineage IC contained orange and blue clusters in their ancestries. The third population (green cluster) indicated mixing with reference isolates from GA. Although genetic admixture between lineages IB and IC was detected across all treatments, *F*_ST_ values were greater than 0.5 (S8 Table) indicating significant differentiation between lineages. The negative values of Tajima’s *D* and Fu & Li’s D* test statistics indicate population growth across treatments and lineages but there was no significant deviation from neutrality (S8 Table).

### Population parameter estimation

**Fig 3:**
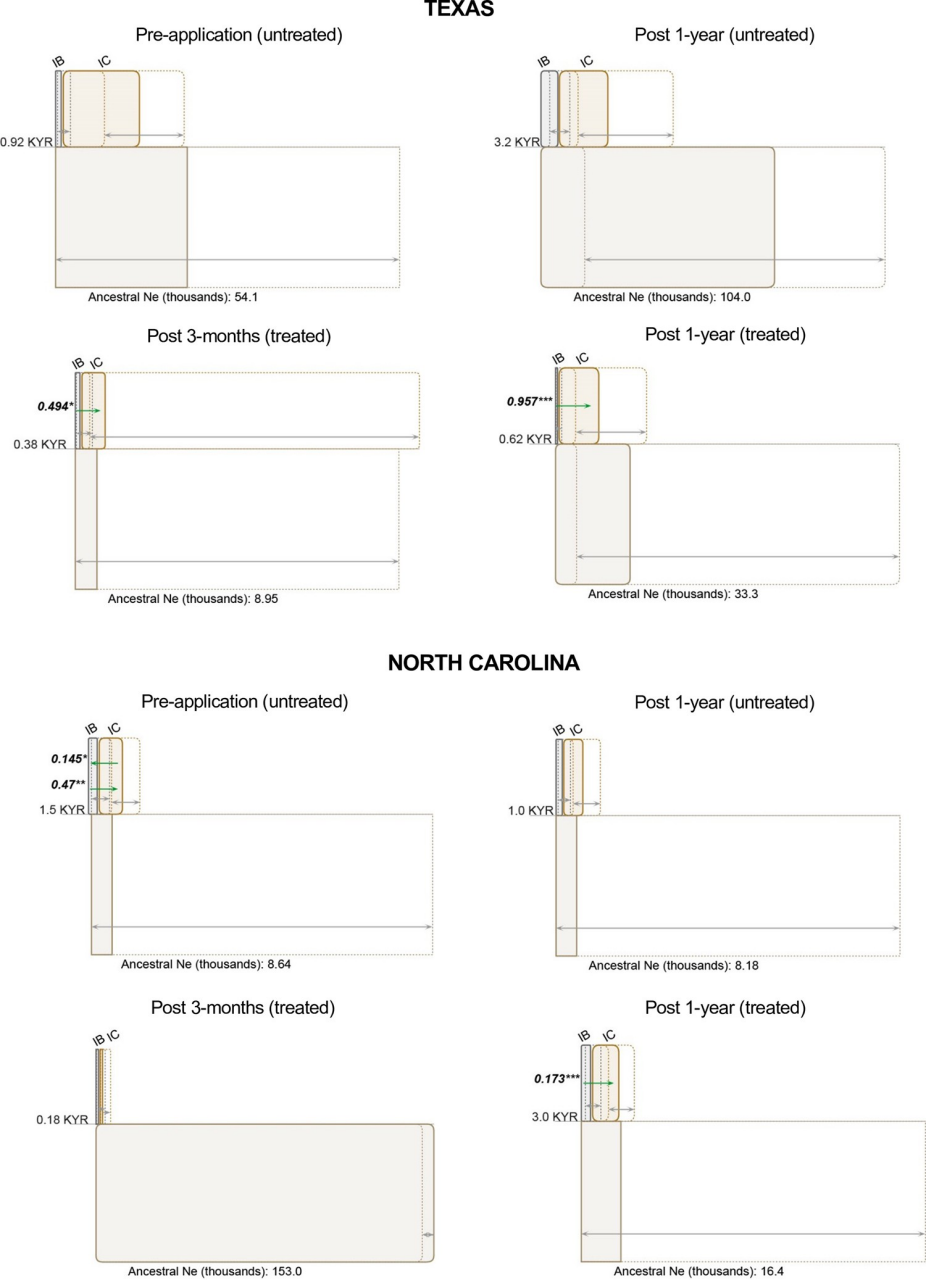
A schematic representation of isolation with migration for *A*. *flavus* populations in TX and NC generated by IMa3 and the IMfig program. The phylogeny for untreated and treated populations is depicted as a hierarchical series of boxes, with ancestor boxes connecting descendant populations of lineages IB and IC, and the width of boxes proportional to the estimated *N*_*e*_. The 95% confidence intervals for each *N*_*e*_ value are shown as dashed lines to the right of the left side of the corresponding population box. Gray arrows to the 95% *N*_*e*_ intervals extend on either side of the right side of each population box. Splitting times, positioned at even intervals, are depicted as solid horizontal lines, with text values on the left in units of thousand years ago (KYA). Migration arrows (in green) indicate the estimated population migration rate (*N*_*e*_*m*) values from one population into another from when the populations diverged from a common ancestor. Arrows are shown only for migration rates that are statistically significant (* *p* < 0.05, ** *p* < 0.01, *** *p* < 0.001). Estimates assumed a generation time of 0.17 years and a mutation rate of 4.2 × 10^−11^ per base per generation.

Patterns of migration differed between untreated and treated populations. In the TX field populations, population migration rates did not differ significantly from zero in the pre-application and post 1-year untreated samples but were significant and unidirectional in the post 3-months and post 1-year treated samples without (Fig 3) or with (S7 Fig) an unsampled ghost population. When bidirectional gene flow was detected, for example, in the NC pre-application (Fig 3) and the AR post 1-year samples (S8 Fig), it was asymmetrical with more significant migration from IB into IC. Even the smaller sample sizes in IN yielded a significant signal of asymmetrical migration into IC (S8 Fig). Across all states and sampled lineages, the ghost populations were much older than the common ancestor of IB and IC populations and there was evidence of significant asymmetrical gene flow from the ghost population into the IC lineage (S7 and S8 Figs).

Population splitting times for lineages IB and IC in the controlled field experiments ranged from 0.38 to 3.2 thousand years ago (KYA) in TX and from 0.18 to 3.0 KYA in NC (Fig 3). Splitting time and effective population size of the common ancestor of the IB and IC lineages in AR were very similar to those in NC (S8 Fig). Effective population sizes of the IC lineage in TX decreased from 31,000 in the pre-application field plots to 11,500 in the post 1-year treated plots (Fig 3). By contrast, effective population sizes in NC for the IC lineage increased from 8,640 before biocontrols were applied to 11,500 in the 1-year post application field plots. Across all states and sampling periods the ghost populations were older and had a larger effective population size relative to lineages IB and IC. The common ancestor of the ghost population and the sampled lineages in TX and NC ranged from 4.0 to 56.0 KYA, which was much older than the range of splitting times (0.23–2.2 KYA) of the common ancestor of the sampled IB and IC lineages (S7 Fig).

Estimates of the time to the most recent common ancestor of lineages IB and IC were much larger in the post 3-years treated TX commercial plots (52.0 KYA) compared to the untreated plots (0.52 KYA) (S9 Fig). However, the estimated splitting time of the common ancestor of the ghost population in the post 3-years treated plots (57.0 KYR) was very similar to the splitting time of lineages IB and IC without the ghost (52.0 KYA). In the model without a ghost, the effective population size of lineage IC in the post 3-year untreated plots was 22,300 which was higher than the 5,300 estimated for the post 3-year treated plots (S9 Fig).

### Mating type distribution

The overall distribution of mating types for the 628 strains across all four states is summarized in S13 Table. Out of a total of 276 unique haplotypes, 168 and 95 were exclusively *MAT1-1* and *MAT1-2*, respectively. There were 13 haplotypes with a mix of *MAT1-1* and *MAT1-2* strains; most notable in this set were haplotypes H1 and H115 which included the strains used in the Afla-Guard and AF36 biocontrol formulations, respectively. There were 72 *MAT1-1* and 105 *MAT1-2* strains in haplotype H1; 8 *MAT1-1* and 11 *MAT1-2* strains in haplotype H115. The other eleven haplotypes were significantly skewed to one mating type which suggests that they comprise at most one or two clonal lineages. Mating type distributions were further examined separately for the untreated and treated plots across each state and sampling period (Table 4). Except for two marginally significant values, overall mating type ratios were approximately 1:1 and not significantly different for both the uncorrected and clone-corrected samples.

**Table 4:** Distribution of *A*. *flavus* mating types across states, treatments, and years ^1^.

	Pre-application		Post 3-months		Post 1-year				Post 3-years			
	Untreated		Treated^2^		Untreated		Treated^2^		Untreated		Treated^3^	
State	*MAT1-1*	*MAT1-2*	*MAT1-1*	*MAT1-2*	*MAT1-1*	*MAT1-2*	*MAT1-1*	*MAT1-2*	*MAT1-1*	*MAT1-2*	*MAT1-1*	*MAT1-2*
**TX**	21(12)	19(9)	13(7)	7(4)	19(11)	30(10)	59(29)	45(20)	31(26)	53(26)	44(20)	33(16)
**NC**	19(15)	20(15)	8(5)	12(3)	29(15)	17(14)	64(37*)	31(20)				
**AR**	20(18)	19(12)	0(0)	20(2)	6(6)	6(4)	26(24)	17(14)				
**IN**	28(12*)	2(2)	1(1)	8(1)	8(7)	2(2)	39(13)	13(8)				

^1^Clone corrected number for each mating type is shown in parentheses based on a two-tailed binomial test; 0.01 < *P < 0.05. Significance for the uncorrected samples is not shown.

^2^Plots were treated with either Afla-Guard or AF36 biocontrol products.

^3^Plots were only treated with Afla-Guard biocontrol.

### Phylogenetic incongruence and recombination

Examination of phylogenetic incongruence across chromosomal phylogenies showed evidence of extensive conflict in deep branches and topological concordance in terminal branches and clades (S10 Fig). As expected, node bipartitions where the descendants were isolates that were very similar genome-wide most likely belonged to the same VCG and showed ≥70 bootstrap support or low conflict across most chromosomes. For example, 22 strains putatively belonging to VCG 24 (i.e., that of the Afla-Guard biocontrol strain) were sampled across TX, IN, NC, and AR in the pre-application field plots (S10A Fig). These strains were monophyletic with IC201 (= Afla-Guard; *MAT1-2*). Two additional strains (TX IC6357; AR IC7641) shared this clade that were *MAT1-1* and descended from a recombinant ancestor with low conflict across all chromosomes (S11 Fig). By contrast, the AF36 strain (= IC1179) which belongs to VCG YV36 shared a recent common ancestor with only one strain in AR (IC7716) and one in TX (IC6169) in the pre-application fields. The largest clade with very similar strains in lineage IC was distributed across TX, IN, and NC and comprised 34 strains that were all *MAT1-1*, most likely members of a large and widely dispersed VCG.

Although strains belonging to the same VCG were grouped together in phylogenies reflecting their common ancestry and close similarity, they also showed evidence of recombination in their evolutionary histories. For example, recombination was detected in the immediate common ancestor of VCG 17 (IC243, IC244; high conflict in chromosomes 7 and 8) and VCG 6 (IC229, IC230; high conflict in chromosomes 2 and 3) (S10A and S11 Figs; S1 Table). Other VCGs were more clonal in their immediate common ancestor such as VCG 5 (IC225, IC226) but there was evidence of recombination one node back which included strain IC7963 (S10A and S11 Figs). There was high conflict (red nodes) across all chromosomes in the deepest branches of the pre-application (S10A and S11 Figs) phylogenies indicating a history of extensive recombination giving rise to the sampled strains; however, there was also the hallmark of recent recombination. For example, IN strain IC7086 in lineage IC clearly inherited chromosome 2 via recombination with a strain in lineage IB (S11 Fig) and inter-lineage recombination was observed with TX strain IC6338, a full-cluster strain in lineage IB (S12 Fig). Nodal support values showed that IC6338 grouped with strains in lineage IC with strong bootstrap support (>70%) in chromosomes 3 and 5 but grouped with strains in lineage IB on other chromosomes (Figs 1 and S12).

Major lineage expansion within lineage IB was observed within a large clade including IC201 strain (= Afla-Guard) in post 1-year treated fields that showed low conflict across all chromosomes (S10C and S13 Figs). Although strains within this clade were very similar in sequence genome-wide, there was a random distribution of both mating types (Table 4), and sampled strains were missing the entire aflatoxin gene cluster. These results are consistent with sexual recombination of closely related indigenous strains with the introduced Afla-Guard biocontrol strain in the post 1-year plots which was not observed in the pre-application fields (S11 Fig). A similar clade that was highly clonal and predominantly *MAT1-1* was observed in lineage IC which did not include IC1179 (= AF36); the latter was in a clade with isolates showing evidence of recombination (red nodes) and a mix of both mating types (S13 Fig). Similar patterns of clonality and recombination were observed in the post 3-years Afla-Guard treated fields in TX (S10D Fig). While strains in terminal nodes showed evidence of shuffling across two or more chromosomes, lineage structure was largely maintained across chromosomal phylogenies except for chromosome 5 where lineage IC was nested within IB. The loss of lineage resolution in chromosome 5 suggests that gene flow was sufficiently high to obscure lineage boundaries (S14 Fig). This was also observed in the post 1-year treated fields (S13 Fig) but not in the post 3-months (S12 Fig) or the pre-application fields (S11 Fig).

### Chromosomal linkage disequilibrium

The release of biocontrol agents into field populations increased opportunities for sexual recombination with indigenous strains and lineages and thereby altered the magnitude of LD in populations (S15 Fig; S14 Table). For example, mean *r*^*2*^ values for chromosome 1 in lineages IB and IC were highest for the post 3-months samples (IB, *r*^*2*^ = 0.300; IC, *r*^*2*^ = 0.212) than in the pre-application samples (IB, *r*^*2*^ = 0.103; IC, *r*^*2*^ = 0.169), and lowest in the post 1-year samples (IB, *r*^*2*^ = 0.053; IC, *r*^*2*^ = 0.068). This translates to the strongest LD in the post 3-month period where clonality was a dominant signature in populations and the weakest LD post 1-year, which is consistent with sexual reproduction breaking down chromosomal LD structure. Similar trends were observed over all chromosomes and sampling periods (S14 Table). As more time elapsed from the initial application of biocontrol, populations returned to equilibrium levels of random mating, as exemplified in the post 3-years TX commercial fields. For example, the LD structure of chromosome 1 in the post 3-year TX field plots was very similar to the pre-application fields (IB, *r*^*2*^ = 0.120; IC, *r*^*2*^ = 0.109). This trend was observed across all chromosomes (S14 Table).

### Aflatoxin production

Quantification of aflatoxin concentrations for representative pre-application isolates from IB and IC lineages showed that overall mean toxin concentrations were lower for lineage IB strains (0.40 μg/mL; SD 0.79) compared to lineage IC (37 μg/mL; SD 19) (S15 Table); Kolmogorov-Smirnov tests based on cumulative distribution functions showed that this difference was significant (*P* < 0.05) (S16 Table). There was a significant decrease in lineage IB mean aflatoxin concentrations (*P* < 0.05) from the pre-application (0.40 μg/mL; SD 0.79) to the post 3-month period (0.06 μg/mL; SD 0.05); similarly, mean toxin concentrations in lineage IC decreased from the pre-application (37 μg/mL; SD 19) to post 3-months (18 μg/mL; SD 18) sampling periods, but this difference was not significant (*P* = 0.56). One year after biocontrol application, mean toxin concentrations between lineages IB (1 μg/mL; SD 1) and IC (57 μg/mL; SD 57) were not significantly different from each other (*P* = 0.08). By contrast, mean toxin concentrations between lineages IB (0.17 μg/mL; SD 0.18) and IC (9 μg/mL; SD 13) in the post 3-year samples (S15 Table) were significantly (*P* < 0.01) different, with 80% of the isolates in lineage IC having a low concentration of B_1_ aflatoxins (<20 μg/mL) (S16 Table). There was agreement in mean aflatoxin concentrations that were analyzed using HPLC-fluorescence and LC-MS detection methods.

## Discussion

We conducted replicated field experiments across four states and performed genotyping by sequencing on corn kernel and soil isolates sampled from native populations before a one-time application of Afla-Guard and AF36 biocontrol agents, and after three months and one year later. We also examined commercial corn fields in TX which demonstrated consistently low aflatoxin concentrations three years after Afla-Guard was applied. This study has three major findings: 1) *A*. *flavus* field populations, before and after the application of biocontrols, are structured by evolutionary lineages IB and IC, with some fields skewed toward one lineage over the other; 2) *A*. *flavus* gene flow is asymmetric and predominantly from lineage IB into IC, with rates from lineage IB into IC being higher (as expected) in fields treated with the Afla-Guard biocontrol agent. The level of gene flow is directly proportional to recombination, genetic diversity, effective population size and mean aflatoxin B1 concentrations in lineage IC; and 3) Use of Afla-Guard can lead to a sustained reduction of aflatoxin contamination. More than 90% of the isolates, sampled from cornfields in TX that had been treated once and three years prior, were from lineage IB and similar to the Afla-Guard strain. Here, we provide evidence of gene flow and sexual recombination as the two most important forces driving diversification in *A*. *flavus* field populations. We show that the fate of introduced biocontrol strains is largely determined by the magnitude and direction of these two forces.

To place this study within the larger context of variation reported in other studies, we performed a combined analysis of the population genomic data from Drott and coworkers [37] with the larger sampling in the present study. We showed that lineages IB and IC, originally defined by Geiser and coworkers [35], are robust evolutionary lineages and can be differentiated using large scale genomic data (S3 Fig) or using only a five-locus (*aflM*, *aflW*, *mfs*, *trpC*, and *amdS*) SNP marker set (S4 and S5 Figs), as reported in previous large scale population genetic studies of *A*. *flavus* [36,39]. There was no evidence of population substructuring within lineages IB and IC based on 1) analyses of genome-scale data in the present study, 2) combined re-analyses of the data in this study and the one published by Drott and coworkers [37], and 3) analyses using the SCAR method which simultaneously models both recombination and migration that occurred in the history of the sample [38].

### Clonality and recombination

Both clonality and recombination structure *A*. *flavus* populations [10,32,36,37,40]. Clonality predominated in the Afla-Guard clade in the pre-application fields across all 4 states, with 16 out of 23 strains having the same *MAT1-2* mating type, missing the entire aflatoxin cluster in a homogeneous genetic background (Fig 2), and sharing a very recent common ancestor as indicated by the very short branches separating strains in the clade (S10A Fig). Similarly, clonality was observed in lineage IC with the largest clade comprising 34 strains that were all *MAT1-1* with a full aflatoxin gene cluster; however, the AF36 biocontrol strain was in a different clade with evidence of recent clonality in the most terminal nodes (i.e., phylogenetic concordance across all chromosomes) and longer interior branches indicative of a history of recombination. Three months after biocontrol application, Afla-Guard was isolated from every plot across all four states, even dominating in untreated and AF36-treated plots; AF36 was not isolated from the Afla-Guard-treated plots (S10B Fig). The spread of biocontrol agents between treatment plots is not an uncommon occurrence [106] and can be attributed primarily to their increased dispersibility, as well as aggressiveness and persistence in soil. Biocontrol stains are reported to persist in soil after one year [107,108], most likely in the form of conidia, which are far more prevalent than sclerotia [109].

Our examination of phylogenetic incongruence provided evidence of sexual recombination in as little as three months after biocontrol applications. For example, there were four TX kernel isolates (IC6346, IC6340, IC6341, IC6345) sampled from the AF36 treated plots that were all *MAT1-1*, had full aflatoxin clusters, and shared a most recent common ancestor with Afla-Guard (Figs 1, S10B and S12). As expected, there was evidence of high phylogenetic conflict (red node) in the common ancestor of these strains in chromosome 3 that harbors the aflatoxin cluster (S12 Fig). This indicates that three months after biocontrols were applied, the Afla-Guard strain from either the applied biocontrol product or a clonal derivative recombined with native lineage IC strains in those plots. Fertilization most likely occurred in the soil where the spores of the Afla-Guard strain (= NRRL 21882) fertilized existing sclerotia of a native IC strain, formed fertile ascocarps, and released ascospores from disintegrated sclerotia, which in turn colonized corn kernels. In previous work, individual sclerotia of NRRL 21882 and AF36 incubated on soil showed extremely low fertility [44], which suggests that conidia of these strains may be more effective as fertilizing agents under field conditions.

Phylogenetic incongruence also identified recombination in a major expansion of lineage IB, which included Afla-Guard one year after biocontrol application (S10C and S13 Figs). Across each state there was evidence of random mating (Table 4) of the Afla-Guard strain with native lineage IB strains that were very genetically similar, and as a result showed very little phylogenetic conflict (S10 Fig). Similarly, there was evidence of recombination of AF36 with native strains (S13 Fig). Previous work showed that AF36 is a putative recombinant with a toxin-producing strain (NRRL 29507) in lineage IC [110] and unlike Afla-Guard, AF36 is infrequently recovered from treated plots [108].

Only five haplotypes (H1, H140, H22, H229, H240) were shared across the pre-application, post 3-months, and post 1-year sampling periods, and there was a high proportion of private haplotypes (96%) across all states (S5 Table). This suggested a high level of recent genetic exchange giving rise to new genotypes and minimal dispersal of haplotypes into plots from other regions. Moreover, there was also a significant clonal component in each lineage, which was sufficient to maintain lineage structure over the long term, even in the presence of genetic exchange and recombination. Changes in LD across different sampling periods can be attributed to the increased recombination activity of the released biocontrol strains with native populations. Biocontrol applications resulted in similar changes in the magnitude of LD across chromosomes for both lineages IB and IC (S14 Table; S15 Fig). LD was highest 3-months after biocontrols were applied showing that clonality predominates in the short term, and LD was at its lowest level one-year later as biocontrols recombined with native strains. These LD patterns were also supported in estimates of the ratio of mutation rate to recombination rate (u/c) on a regional level, with the highest u/c levels observed post 3-months after biocontrol application and decreasing in post 1-year with increasing introgression and inter-lineage recombination (S8 Table).

### VCGs and genetic diversity

The consistent grouping of *A*. *flavus* reference isolates with VCG suggests that genome-wide haplotypes are a good proxy for VCG. Putative recombinants between lineages IB and IC would have genetic backgrounds of both lineages, show evidence of phylogenetic incongruence across chromosomes, and have long branches in Neighbor-net networks which is indicative of divergent sequences. For example, IC6338 from the post 3-months TX sample contained an almost even split of both lineages IB and IC in its genetic background (Figs 1 and 2; S10 Table) and demonstrated high conflict across chromosomes 4, 6, 8 (red nodes) in the immediate common ancestor (S10B and S12 Figs). By contrast, IC6344 and IC6345 from TX shared a recent common ancestor, a single genetic background and low phylogenetic conflict (cyan nodes) across all chromosomes (S10B and S12 Figs). The low conflict is most likely the result of recombination with closely related members in the same lineage, which does not change the lineage background but reduces phylogenetic resolution. In the case of clonality, we would expect to see strong topological congruence (black nodes) across most chromosomes in the immediate common ancestor, as observed with our reference isolates that are members of the same VCG, such as, VCG 1 (IC217, IC218), VCG 4 (IC221, IC222), and VCG 5 (IC225, IC226) (S10 Fig). Populations have a high degree of clonality, which can be especially evident if sampling only VCGs with multiple representatives [111–115] but the evidence is clear in the present study and previous research [10,32,33,37,39,40] that natural *A*. *flavus* populations comprise both frequently sampled VCGs and many singleton VCGs that contribute to genetic diversity. A sampling scheme that includes the full range of VCG diversity will show that recombination is driving genetic and mycotoxin diversity in *A*. *flavus*. This is exemplified by the high frequency of singleton multilocus genome-wide haplotypes (i.e., very good proxy of VCGs) that show quantitative variation in aflatoxin production, as previously reported [32,102].

### Magnitude and direction of inter-lineage introgression

One year after biocontrol application, treated plots in TX, NC and AR showed a significant change in the relative frequency of each lineage (i.e., lineage skew) after clone correction based on a two-tailed binomial test, with lineage IB being the dominant lineage in TX and lineage IC predominating in NC and AR (Table 3). A similar significant skew to lineage IC was observed in untreated plots prior to biocontrol applications in NC and AR. Because field plots did not have a previous history of biocontrol applications, this suggests that an *A*. *flavus* lineage skew can occur naturally in field populations. One year later both the untreated and treated plots in TX and NC showed a similar lineage skew, where lineage IB was more predominant in TX and a reversal in NC where lineage IC was more frequently sampled. In the post 1-year untreated plots in TX, we cannot rule out that the weakly significant (0.01 < *P* < 0.05) difference in lineage frequencies is the result of cross-contamination from dispersal of biocontrol strains among plots in a field. This does not compromise the results because plots treated with biocontrols showed more significant (*P* < 0.01) asymmetry in lineage frequencies (Table 3) and gene flow estimates were significantly higher and predominantly asymmetric in the treated plots compared to the untreated (Fig 3).

Results from IMa3 runs showed that gene flow, when present, is predominantly from lineage IB to IC (Fig 3). This is supported by the limited structure observed in lineage IB and the highly structured populations in lineage IC that harbor varying proportions of IB genetic clusters (Figs 1 and 2; S9–S12 Tables). This was also reflected in nucleotide diversity estimates, which were consistently higher in lineage IC than in IB (S8 Table). The signature of admixture between lineages IB and IC was also evident in Neighbor-net networks across the four sampling periods with many isolates occupying intermediate positions between lineages IB and IC (Fig 1). These observations are consistent with results from a re-analysis of the data from Drott and coworkers [37] with evidence of recombinant strains from both multilocus analysis based on SNP variation at five target loci (S4 and S5 Figs) and genome-scale resolution (S2 and S3 Figs).

The IMa3 program was used to quantify the magnitude and direction of introgression between lineages IB and IC. Since introgression was asymmetrical from IB into IC, larger migration estimates translated to greater genetic admixture of isolates in the receiving population. Migration rate parameters did not differ significantly from zero in the pre-application and post 1-year (untreated) fields in TX. Significant and high unidirectional gene flow in the direction of IC was detected in the post 3-months period (0.494; *P* < 0.05) with even higher rates of introgression (0.957; *P* < 0.001) one year after biocontrol application (Fig 3). Migration rate values greater than 0.5 are considered high in simulation studies [116]. The effective population size of the IC lineage in post 1-year (17,700) was double, compared to post 3-months (9,000) field plots. While lineage IC effective population sizes were very similar in the pre-application and post 1- year (untreated) plots, lineage IB effective population size more than tripled from the pre-application (2,300) to the post 1-year (untreated) samples (7,400). The increase in effective population size can be the result of higher sexual fertility of isolates in lineage IB in TX or dispersal of Afla-Guard spores from adjacent subplots that were treated, or both. Divergence time estimates between lineages IB and IC in TX ranged from 920 YR in the pre-treatment plots to 63,000 YR in the post 1-year samples (Fig 3), consistent with divergence times reported for three common VCGs in TX [111].

Similar patterns of gene flow were observed in the NC field plots. For example, there was an increase in lineage IC effective population size in the post 1-year treated plots (11,500) compared to the post 3-months plots (1,300), and there was also evidence of significant and moderate unidirectional gene flow (0.173; *P* < 0.001) from lineage IB into IC (Fig 3). Although samples sizes were small, significant asymmetrical migration from IB into IC was also observed in the pre-application fields in AR (0.172; *P* < 0.01) and even higher migration estimates were observed in IN (0.552; *P* < 0.001) (S3 Fig). Gene flow from IB into IC was further supported by the phylogenetic incongruence observed for IC7086, a strain sampled prior to biocontrol application in IN that grouped with lineage IB strains in chromosome 2 and with lineage IC strains at all other chromosomes (Figs 1 and S11). The most striking shift was observed in the TX commercial fields 3-years after treatment with Afla-Guard, where the effective population size of lineage IC in the untreated fields (22,330) decreased to only 5,300 individuals in the treated, almost eliminating lineage IC from the Afla-Guard treated plots (Table 3; S9, S10D and S14 Figs). By comparison, lineage IB effective population size increased more than 3-fold from the untreated (827) to the Afla-Guard treated (2,800) plots, and there was evidence of significant and asymmetrical gene flow from lineage IB into IC in the untreated (1.26; *P* < 0.01) and treated (0.24; *P* < 0.001) plots (S9 Fig). The post 3-years lineage skew observed for untreated and treated plots in TX was very similar to the lineage frequencies observed in the post 1-year field plots (Table 3), which suggests that an Afla-Guard-driven population shift in TX is sustainable for at least three years.

### Lineage-specific differences in fertility

Mechanistically, any difference in fertility between lineages IB and IC can result in disproportionate mating and a signature of asymmetrical introgression. For example, laboratory crosses demonstrated that when the IC278 strain from lineage IC served as the sclerotial (maternal) parent and the Afla-Guard strain from the IB lineage as the conidial (paternal) parent, about 97% of the sclerotia were fertile compared to only 1% when the Afla-Guard strain was used as the sclerotial parent. A plausible scenario in the field is that biocontrol inoculum (conidia) fertilized sclerotia that had accumulated in soil before treatments, and in that case the biocontrol strain would function as a paternal parent. It is not known if progeny from inter-specific crosses preferentially mate with the maternal or paternal parent, which would further shift the population composition to lineage IC. This process could potentially maintain the lineage structure observed in NC and AR. Since fertile matings are possible within each lineage [32] the population shift can go in the direction of either lineage depending on their relative fertilities and population sizes. The degree of fertility could be dependent on strain-specific variability in gene expression of fungal mating type pheromones and receptors which are up-regulated in high fertility crosses [117].

The large effective ghost population size in both the post 1-year untreated and treated plots (S7 Fig), suggests the presence of an unsampled sister population that shares a common ancestor with the IB lineage. A possible candidate for the sister population would be the IA lineage, which is known to be closely related to the IB lineage and has been sampled in TX [35,118]. Lineage IA contains a mix of small (S) and large (L) sclerotial-producing strains whereas IB and IC lineages are predominantly of the L type.

The observed significant differences in lineage frequencies across states may be due to either latitudinal differences in clonal population densities of lineages or fertility differences among lineages such that lineage IB is more abundant and fertile in southern subtropical regions and IC in the northern more temperate regions. This suggests that *A*. *flavus* biocontrol strains that belong to lineage IB would be more effective as biocontrol agents in Texas since there are more opportunities for mating with compatible strains in lineage IB. Moreover, the larger population size of IB would result in more introgression of IB into IC and greatly reduce aflatoxin contamination of crops, as observed in the TX fields three years after biocontrol application. By contrast, in NC and AR, lineage IC predominates and is more genetically diverse than IB after biocontrol application; in these regions, asymmetrical gene flow from IB into IC will increase population sizes of the IC lineage.

### Biocontrol implications

From a biocontrol perspective, the enhanced introgression of sexually compatible lineage IB strains into native populations offers the potential for sustained reductions in aflatoxin levels over subsequent generations. This suggests that the evolutionary lineage trait may be stronger than any strain-specific differences in reducing aflatoxin levels across different latitudes and environmental conditions. Even single strain formulations from lineage IB can persist if environmental conditions are favorable to their growth, and native populations are fertile enough with the introduced lineage IB strain to drive and maintain sexual reproduction. For example, the Afla-Guard strain has been shown to be effective in mitigating aflatoxin contamination in Texas [119,120] and North Carolina [121,122]. A potential pitfall is that biocontrol-treated plots could have a higher incidence of ears with moldy grain. Typically, ears in the field may have some Aspergillus ear rot on kernels at the tip of the ear. These moldy kernels are smaller and lighter than the others and are usually blown out of the back of the combine. In a small plot experiment, treatment with Afla-Guard did not impact yield of the combined plot [123]. Moreover, farmers in Texas who have used nonaflatoxigenic strains for aflatoxin control have not reported a yield drag or decreased grain quality. However, with a nonaflatoxigenic strain treatment, there is a higher incidence of symptomless infection of healthy-looking kernels [106,124]. This usually does not cause a problem in harvested corn entering commerce, but it could cause problems in grain quality if treated corn is stored in leaky bins or is otherwise rehydrated.

Aflatoxin production in lineage IB was significantly lower than in lineage IC in the pre-application fields. This lineage difference in aflatoxin producing potential was also reported for native populations of *A*. *flavus* in the US [37] and Argentina [39]. Lab experiments have shown that aflatoxin production is highly heritable and any inter-lineage mating would increase the potential for aflatoxin production in both lineages [32]. The higher rates of gene flow and recombination in the post 1-year samples may explain the similar aflatoxin B_1_ distributions for lineages IB and IC. Results from Kolmogorov-Smirnov tests across different sampling periods (S16 Table) showed that aflatoxin distributions in the post 3-year TX fields were significantly different between lineages, and cumulative probability distributions showed that 80% of the isolates in lineage IC had a low aflatoxin concentration (<20 μg/mL). This was less than the corresponding aflatoxin concentration for 80% of the lineage IC isolates in the pre-treatment (<40 μg/mL), post 3-month (<40 μg/mL), and post 1-year (<140 μg/mL).

Although both Afla-Guard and AF36 biocontrol products are effective in reducing aflatoxin levels in the short term (post 3-months), their efficacy in the long-term (post 1-year) may depend on which lineage they belong to. Because lineage IB isolates are predominantly nonaflatoxigenic, populations with a greater proportion of lineage IB strains relative to lineage IC are predicted to have lower aflatoxin levels. In TX fields where aflatoxin levels were consistently low (10–33 ppb) over several years, there was a larger proportion of lineage IB isolates compared to IC. Moreover, lineage IC in the TX commercial corn fields was predominantly sampled in the untreated plots (S10D Fig). While the TX commercial cornfields contained more isolates that were genetically like lineage IB, they were functionally a mix of low aflatoxin-producing and nonaflatoxigenic strains. Any balancing selection acting to maintain aflatoxin producers and non-producers in the population [10,125] can continue but the targets of selection are now predominantly low aflatoxin producers within lineage IB. This suggests that it might be possible to shift *A*. *flavus* populations to a state that is functionally and qualitatively similar to the native population but quantitatively have a much-reduced aflatoxin footprint than the native population. This has significant implications for reducing aflatoxin contamination in regions where lineage IC predominates such as in NC and AR.

### Conclusion and perspectives

The high VCG diversity in *A*. *flavus* soil populations translates to a range of potential mating partners thereby increasing opportunities for successful encounters with highly fertile sclerotia. Thus, selecting a mix of biocontrol strains from lineage IB that are of different mating types and VCGs (i.e., clonal lineages) may be a useful strategy for increasing the number of successful matings in field populations [126]. Preliminary field results of biocontrol formulations containing a mix of sexually compatible strains in NC [127] and TX (Isakeit, unpublished data) show that they outperform single strain formations in reducing aflatoxin concentrations and increasing corn yields. This strategy of including strains from lineage IB that are sexually compatible may explain the increased efficacy observed in biocontrol products that include a mix of nonaflatoxigenic stains. For example, the Aflasafe® biocontrol product comprises four nonaflatoxigenic *A*. *flavus* strains (La3279, Og0222, Ka16127, La3304) that have either a partial deletion in the aflatoxin cluster (Ka16127 and La3304) or are completely missing the cluster (La3279 and Og0222) [128–130]. These four strains belong to different VCGs and are phylogenetically distinct from AF36 and NRRL 3357 yet genetically very similar to *A*. *oryzae* [129,131], which suggests that they are likely members of lineage IB. Although mating type was not a criterion in selecting these strains they are fortuitously representative of both mating types (*MAT1-1*, La3279 and Og0222; *MAT1-2*, Ka16127 and La3304) [129]. When compared to single strain formulations, we expect strains that are of different mating types to greatly increase effective population sizes and result in an even larger disproportionate mating access to sexually fertile strains. Future studies will examine this possibility.

## Supporting information

S1 Fig Scoring of aflatoxin cluster configurations using JBrowse and the *A*. *oryzae* RIB40 reference genome. **A.** Partial/full/missing clusters for reference (ref) strains and representative isolates from the present study. **B.** A zoom in on the Fig in **A** showing the cluster boundaries for partial cluster strains. **C.** Top panel shows location of dehydrogenase/ketoreductase gene flanking the left side of the cluster breakpoint (vertical red line) for definitive assignment of partial-C and partial-E cluster strains in the present study; lower panel shows location of cytochrome P450 gene on the right side of the cluster breakpoint (red line) in partial-C deletion strains. **D.** Top panel shows location of dehydrogenase/ketoreductase gene flanking the left side of the cluster breakpoint (vertical red line) for definitive assignment of partial-C and partial-G cluster strains in Drott *et al* 2020 [37] (indicated with a “D” prefix); middle panel shows location of cytochrome P450 gene on the right side of the cluster breakpoint (red line) in partial-C deletion strains; lower panel shows configuration of missing clusters in Drott *et al* 2020 [37] and the additional sequence beyond the cluster breakpoint (vertical red line) in D23.
(PDF)

S2 Fig Inferred Neighbor-net networks for 94 *A*. *flavus* isolates from Drott *et al* 2020 [37]. **A.** Network based on 817,774 SNPs using *A*. *oryzae* RIB40 reference genome showing the population structure reported in Drott *et al* 2020 [37]. The circles denote putative hybrid strains that were inferred from the multilocus analysis of *aflM*, *aflW*, *mfs*, *trpC*, and *amdS* in S4 Fig. **B.** Network based on 5,870 SNPs from in silico ddRADseq using *A*. *oryzae* RIB40 reference genome. The branches in the network are drawn to scale and the scale bar represents 0.01 substitutions per site.
(PDF)

S3 Fig Inferred Neighbor-net network and PCA analysis for the combined analysis of strains in the present study and Drott *et al* 2020 [37] using the *A*. *oryzae* RIB40 reference genome. **A.** Network based on 6,833 SNPs across 907 *A*. *flavus* isolates. Strain names are provided only for the isolates from Drott *et al* 2020 [37] (indicated with a “D” prefix). Population A from Drott *et al* 2020 [37] is clearly subdivided and population C is nested in lineage IC with other isolates from the present study; population B falls exclusively in lineage IB. The circles denote putative hybrid strains that were inferred from the multilocus analysis of *aflM*, *aflW*, *mfs*, *trpC*, and *amdS* in S4 Fig. The branches in the network are drawn to scale and the scale bar represents 0.01 substitutions per site. **B.** PCA and cluster analysis assigns all isolates into one of two distinct evolutionary lineages: IB and IC.
(PDF)

S4 Fig Lineage structure based on multilocus analysis of the data from Drott *et al* 2020 [37]. **A.** Lineage structure based on multilocus analysis of *aflM*, *aflW*, *mfs*, *trpC*, and *amdS*. At the extremes of the network are strains in lineages IB (left side) and IC (right side) and in the middle are putative inter-lineage hybrids. The position of these putative hybrid strains is shown in the larger genome-scale networks in S2 and S3 Figs. **B.** Overlay of cluster configurations on the multilocus network showing distribution of missing, partial, and full aflatoxin gene clusters. The branches in the network are drawn to scale and the scale bar represents 0.01 substitutions per site.
(PDF)

S5 Fig Concatenated multilocus sequence matrix for putative hybrid strains showing signature of recombination between lineages IB and IC. The eleven sequences highlighted in green share a unique haplotype in the *aflM* region and are putative hybrids inferred in the multilocus network in S4 Fig and assigned to population A in Drott *et al* 2020 [37]; the 19 strains highlighted in red for the *mfs*, *trpC* and *amdS* share a distinct multilocus haplotype and are missing the entire aflatoxin gene cluster (except for D82) and assigned to population B in Drott *et al* 2020 [37]. Strain D82 is a full cluster strain that is a putative inter-lineage recombinant between populations A (= lineage IC) and B (= lineage IB).
(PDF)

S6 Fig Population structure using principal component analysis (PCA). **A.** PCA scatter plots for 815 *A*. *flavus* isolates showing clusters, lineages and sampling period for the networks shown in Fig 1. **B.** For each treatment time point two PCA scatter plots are shown for genome-wide variation in *A*. *flavus* across TX, NC, AR and IN (reference strains are from GA and AZ). The PCA cubes show the distribution of individuals based on their membership in one of two clusters inferred from the Gap statistic and overlaid with lineage (PCA cube on left) or state (PCA cube on right). The color scheme and shapes are unique for each PCA cube.
(PDF)

S7 Fig A schematic representation of isolation with migration for *A*. *flavus* populations in TX and NC including an unsampled ghost population. The phylogeny is depicted as a hierarchical series of boxes, with ancestor boxes connecting descendant populations of lineages IB and IC, and the width of boxes proportional to the estimated *N*_*e*_. The 95% confidence intervals for each *N*_*e*_ value are shown as dashed lines to the right of the left side of the corresponding population box. Gray arrows to the 95% *N*_*e*_ intervals extend on either side of the right side of each population box. Splitting times, positioned at even intervals, are depicted as solid horizontal lines, with text values on the left in units of thousand years ago (KYA). Migration arrows (in green) indicate the estimated population migration rate (*N*_*e*_*m*) values from one population into another from when the populations diverged from a common ancestor. Arrows are shown only for migration rates that are statistically significant (* *p* < 0.05, ** *p* < 0.01, *** *p* < 0.001). Estimates assumed a generation time of 0.17 years and a mutation rate of 4.2 × 10^−11^ per base per generation.
(PDF)

S8 Fig A schematic representation of isolation with migration for *A*. *flavus* populations in untreated and treated field populations in AR and IN. **A.** Without a ghost population. **B.** With a ghost population. The phylogeny is depicted as a hierarchical series of boxes, with ancestor boxes connecting descendant populations of lineages IB and IC, and the width of boxes proportional to the estimated *N*_*e*_. The 95% confidence intervals for each *N*_*e*_ value are shown as dashed lines to the right of the left side of the corresponding population box. Gray arrows to the 95% *N*_*e*_ intervals extend on either side of the right side of each population box. Splitting times, positioned at even intervals, are depicted as solid horizontal lines, with text values on the left in units of thousand years ago (KYA). Migration arrows (in green) indicate the estimated population migration rate (*N*_*e*_*m*) values from one population into another from when the populations diverged from a common ancestor. Arrows are shown only for migration rates that are statistically significant (* *p* < 0.05, ** *p* < 0.01, *** *p* < 0.001). Estimates assumed a generation time of 0.17 years and a mutation rate of 4.2 × 10^−11^ per base per generation.
(PDF)

S9 Fig A schematic representation of isolation with migration for *A*. *flavus* populations in untreated and treated TX commercial fields. **A.** Without a ghost population. **B.** With a ghost population. The phylogeny is depicted as a hierarchical series of boxes, with ancestor boxes connecting descendant populations of lineages IB and IC, and the width of boxes proportional to the estimated *N*_*e*_. The 95% confidence intervals for each *N*_*e*_ value are shown as dashed lines to the right of the left side of the corresponding population box. Gray arrows to the 95% *N*_*e*_ intervals extend on either side of the right side of each population box. Splitting times, positioned at even intervals, are depicted as solid horizontal lines, with text values on the left in units of thousand years ago (KYA). Migration arrows (in green) indicate the estimated population migration rate (*N*_*e*_*m*) values from one population into another from when the populations diverged from a common ancestor. Arrows are shown only for migration rates that are statistically significant (* *p* < 0.05, ** *p* < 0.01, *** *p* < 0.001). Estimates assumed a generation time of 0.17 years and a mutation rate of 4.2 × 10^−11^ per base per generation.
(PDF)

S10 Fig Phylogenetic incongruence using the Hypha module in Mesquite and displayed using T-BAS. Phylogenetic incongruence between each chromosome and mitochondrial genome phylogeny relative to the total evidence display tree for the four different sampling time points (A-D) is shown using grids on node partitions. Branch lengths on the total evidence tree are drawn to scale and the scale bar is shown at the top. In each grid, bootstrap support values are displayed with each box from left to right representing one of eight chromosomes; the box on the bottom right is for the mitochondrial genome. Colors in grids represent node bipartitions that were supported at a bootstrap support value ≥70% (black color), <70% (white color), and missing or inapplicable (grey color). Phylogenetic incongruency was represented as high conflict (red color) and low conflict (cyan color). Additional attributes (lineage, state, mating type, AF cluster configuration, and substrate/treatment) are shown in columns adjacent to the strain names.
(PDF)

S11 Fig Phylogenetic incongruence of each chromosome and mitochondrial genome phylogeny relative to the total evidence tree for isolates sampled before biocontrol application. In the total evidence display tree colors in grids represent node bipartitions that were supported at a bootstrap support value ≥70% (black color), <70% (white color), and missing or inapplicable (grey color). Phylogenetic incongruency was represented as high conflict (red color) and low conflict (cyan color). Strain names are highlighted to show their lineage membership in the total evidence tree; the mitochondrial genome has insufficient variation and poor resolution of lineage structure. The red arrows track the position of strain IC7086 sampled from IN which belongs to lineage IC; the black arrows track the position of IC6357 sampled from TX which is in lineage IB. Strain IC7086 is grouping with lineage IB strains in chromosome 2 but is placed in lineage IC on all other chromosomes with strong bootstrap support (≥90%). Strain IC6357 groups only with lineage IB strains on different chromosomes but with weak bootstrap support (<70%).
(PDF)

S12 Fig Phylogenetic incongruence of each chromosome and mitochondrial genome phylogeny relative to the total evidence tree for isolates sampled 3-months after biocontrol application. In the total evidence display tree colors in grids represent node bipartitions that were supported at a bootstrap support value ≥70% (black color), <70% (white color), and missing or inapplicable (grey color). Phylogenetic incongruency was represented as high conflict (red color) and low conflict (cyan color). Strain names are highlighted to show their lineage membership in the total evidence tree; the mitochondrial genome has insufficient variation and poor resolution of lineage structure. The red arrows track the position of strain IC6338 sampled from TX which belongs to lineage IB in the total evidence tree. Strain IC6338 groups in lineage IB in chromosomes 1, 2, 4, 6, 7 and 8, but there is strong bootstrap support (>70%) for IC6338 grouping with strains in lineage IC in chromosomes 3 and 5.
(PDF)

S13 Fig Phylogenetic incongruence of each chromosome and mitochondrial genome phylogeny relative to the total evidence tree for isolates sampled 1-year after biocontrol application. In the total evidence display tree colors in grids represent node bipartitions that were supported at a bootstrap support value ≥70% (black color), <70% (white color), and missing or inapplicable (grey color). Phylogenetic incongruency was represented as high conflict (red color) and low conflict (cyan color). Strain names are highlighted to show their lineage membership in the total evidence tree; the mitochondrial genome has insufficient variation and poor resolution of lineage structure. The red arrows track the position of strain IC15721 sampled from IN which belongs to lineage IB in the total evidence tree but sharing recent common ancestry with strains in both lineages across most chromosomes making lineage assignment difficult. The black arrows track the position of the AF36 biocontrol strain, IC1179, which belongs to lineage IC and has low phylogenetic conflict with other stains in that lineage suggesting a history of recombination.
(PDF)

S14 Fig Phylogenetic incongruence of each chromosome and mitochondrial genome phylogeny relative to the total evidence tree for isolates from commercial TX cornfields sampled 3-years after application of the Afla-Guard biocontrol strain. In the total evidence display tree colors in grids represent node bipartitions that were supported at a bootstrap support value ≥70% (black color), <70% (white color), and missing or inapplicable (grey color). Phylogenetic incongruency was represented as high conflict (red color) and low conflict (cyan color). Strain names are highlighted to show their lineage membership in the total evidence tree; the mitochondrial genome has insufficient variation and poor resolution of lineage structure. The red arrows track the position of strain IC14733 which belongs to lineage IB and shares very recent common ancestry with IC14744 and IC14769 with strong bootstrap support in chromosomes 1, 3, 5, 6 and 8; however, IC14733, IC14744 and IC14769 are also grouping with IC14720 with strong bootstrap support in chromosomes 2, 4, and 7.
(PDF)

S15 Fig Chromosomal LD plots for *A*. *flavus* lineages IB and IC. LD plots are displayed using Haploview for each chromosome across four different sampling time points for lineages IB and IC. In each LD plot, black lines outline the edge of the spine of strong LD. In the coloring scheme, red represents strong LD (LOD ≥ 2, D’ = 1), shades of pink/red represent intermediate LD (LOD ≥ 2, D’ < 1), blue represents weak LD (LOD < 2, D’ = 1) and white represents no LD (LOD < 2, D’ < 1).
(PDF)

S1 Table State, substrate, treatment, lineage, sampling period, mating type, AF cluster and GWH for the 815 *A*. *flavus* strains examined in this study. (XLSX)

S2 Table Source and genetic characterization of *A*. *flavus* reference isolates. (XLSX)

S3 Table Frequency distribution of genome-wide haplotypes for 628 *A*. *flavus* strains and haplotypes with two or more strains. (XLSX)

S4 Table Distribution of *A*. *flavus* genome-wide haplotypes (GWH) across all four states and treatments. (XLSX)

S5 Table Distribution of *A*. *flavus* haplotypes across the three different sampling periods. (XLSX)

S6 Table *A*. *flavus* aflatoxin cluster structure in kernel and soil samples across different states and sampling periods. (XLSX)

S7 Table TX commercial fields. Proportion of *A*. *flavus* lineages IB and IC in kernel samples in TX commercial fields before biocontrol application and 3 years later. The frequency of *A*. *flavus* aflatoxin cluster types in TX commercial fields before biocontrol application and 3 years later, and the fraction of *A*. *flavus* strains with the Afla-Guard haplotype in TX commercial fields before biocontrol application and 3 years later.
(XLSX)

S8 Table Genome-wide population parameters estimates in *A*. *flavus* across sampling period, lineage, state, and treatment. (XLSX)

S9 Table Genetic ancestry of *A*. *flavus* populations in pre-treatment field plots from STRUCTURE. (XLSX)

S10 Table Genetic ancestry of *A*. *flavus* populations in post 3-months field plots from STRUCTURE. (XLSX)

S11 Table Genetic ancestry of *A*. *flavus* populations in post 1-year field plots from STRUCTURE. (XLSX)

S12 Table Genetic ancestry of *A*. *flavus* populations in post 3-year TX commercial field plots from STRUCTURE. (XLSX)

S13 Table Distribution of mating types for 628 *A*. *flavus* strains. Summary of mating type counts across haplotypes. List of haplotypes (*n* = 13) with a mix of *MAT1-1* and *MAT1-2* strains, haplotypes (*n* = 168) that are exclusively *MAT1-1*, and haplotypes (*n* = 95) that are exclusively *MAT1-2*.
(XLSX)

S14 Table *A*. *flavus* chromosomal LD across different lineages and sampling periods. (XLSX)

S15 Table Aflatoxin B1 concentrations. Mean concentrations based on three replicates for 99 isolates of *A*. *flavus* across different sampling periods, including reference strains. Estimates of mean aflatoxin concentration and standard deviation across lineage and sampling period.
(XLSX)

S16 Table Probabilities of Kolmogorov-Smirnov tests across different sampling periods. (XLSX)
