# Supplementary material for: Asymmetrical lineage introgression and recombination in populations of Aspergillus flavus: Implications for biological control
Source: PLoS One. 2022 Oct 27;17(10):e0276556. doi: 10.1371/journal.pone.0276556 (PMC9620740; doi:10.1371/journal.pone.0276556)
Supplement: S1 Fig — A. Partial/full/missing clusters for reference (ref) strains and representative isolates from the present study. B. A zoom in on the Fig in A showing the cluster boundaries for partial cluster strains. C. Top panel shows location of dehydrogenase/ketoreductase gene flanking the left side of the cluster breakpoint (vertical red line) for definitive assignment of partial-C and partial-E cluster strains in the present study; lower panel shows location of cytochrome P450 gene on the right side of the cluster breakpoint (red line) in partial-C deletion strains. D. Top panel shows location of dehydrogenase/ketoreductase gene flanking the left side of the cluster breakpoint (vertical red line) for definitive assignment of partial-C and partial-G cluster strains in Drott et al 2020 [37] (indicated with a “D” prefix); middle panel shows location of cytochrome P450 gene on the right side of the cluster breakpoint (red line) in partial-C deletion strains; lower panel shows configuration of missing clusters in Drott et al 2020 [37] and the additional sequence beyond the cluster breakpoint (vertical red line) in D23. (PDF) [file pone.0276556.s001.pdf]

A. Scoring aflatoxin cluster configuration as partial/full/missing in Molo *et al* using JBrowse

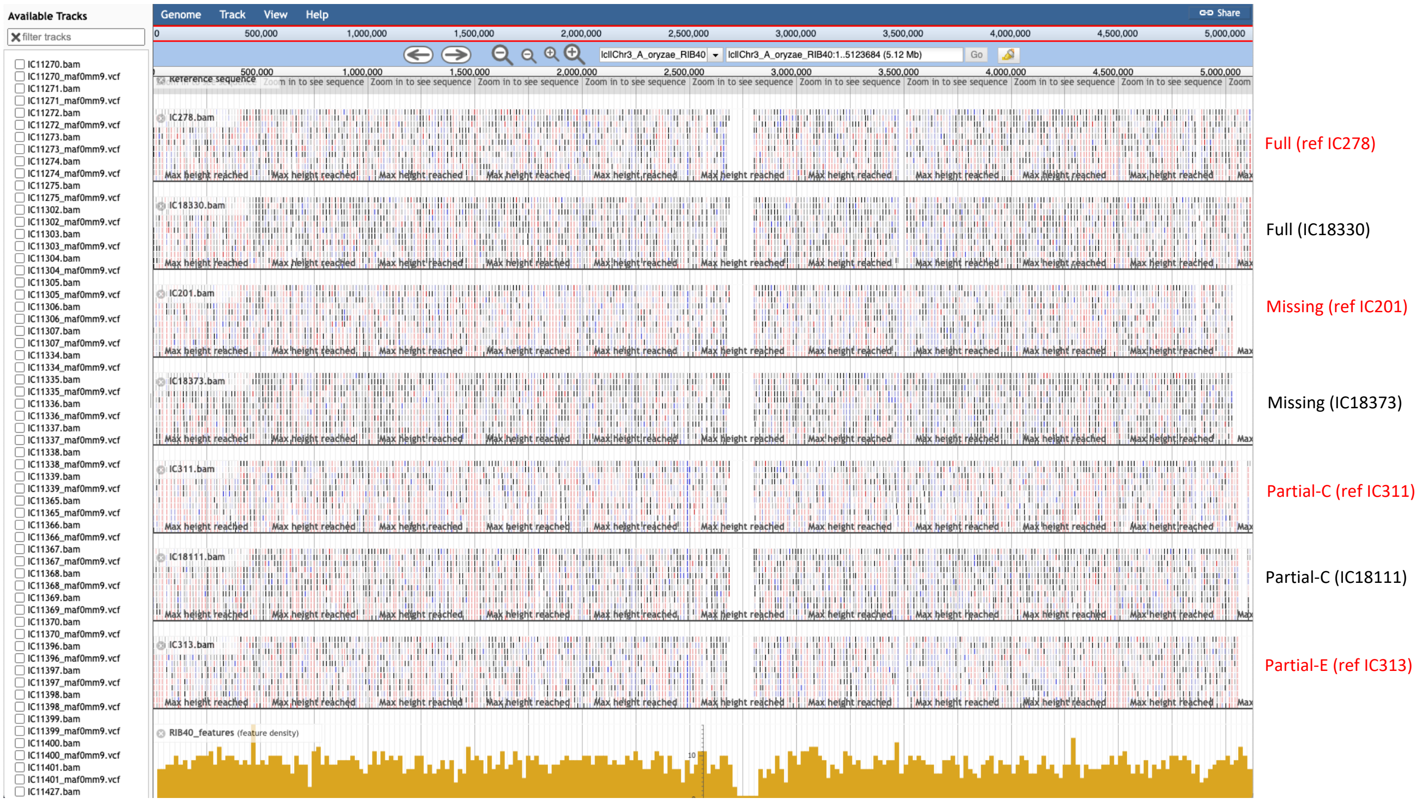

B. Zoom in of partial/full/missing cluster configurations in Molo *et al*

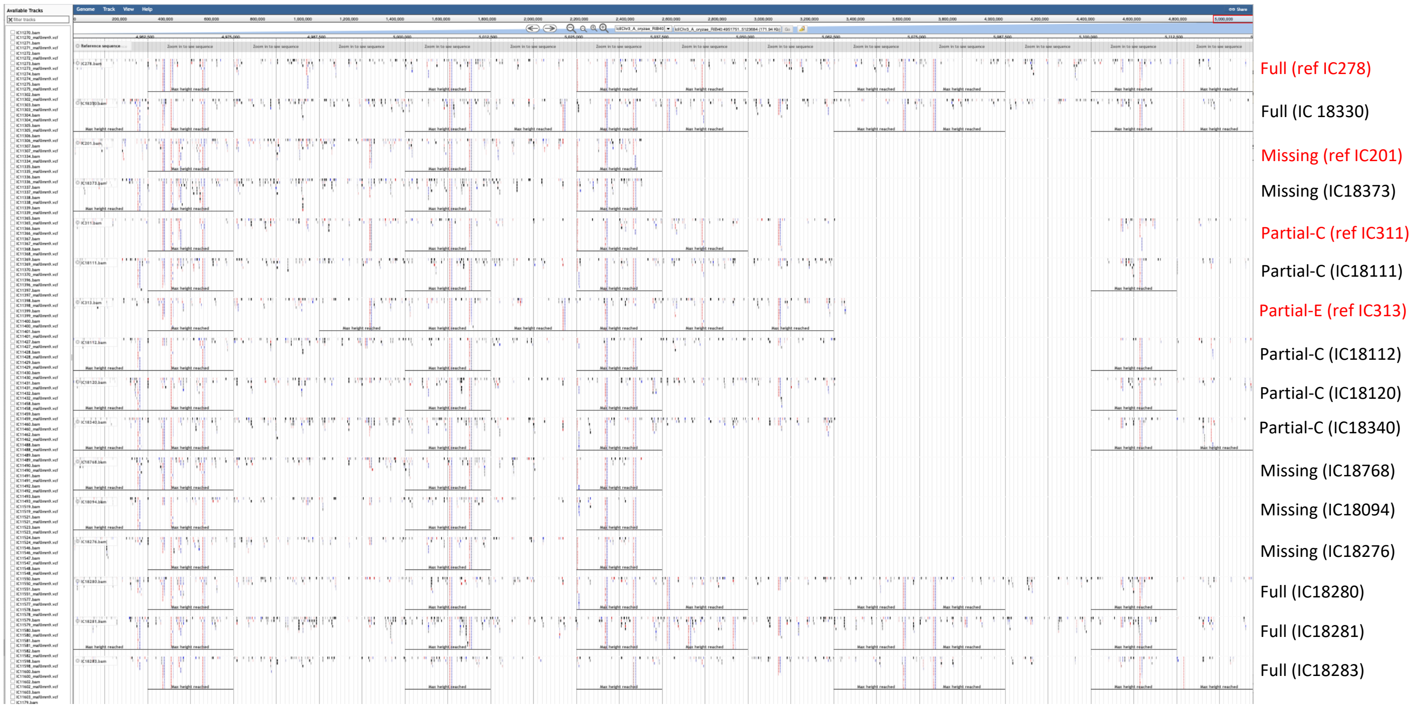

C. Identification of cluster breakpoints in Molo *et al*

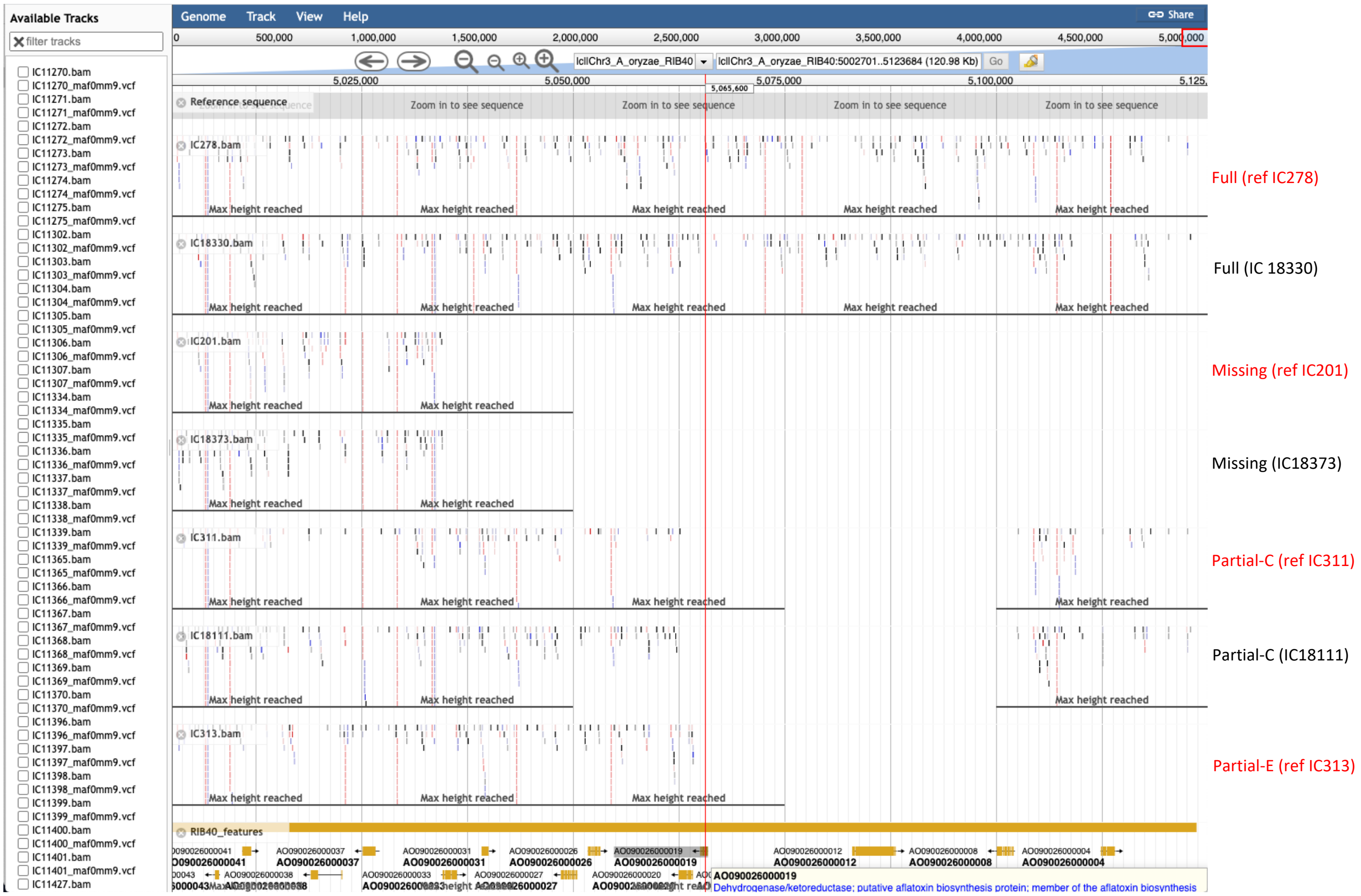

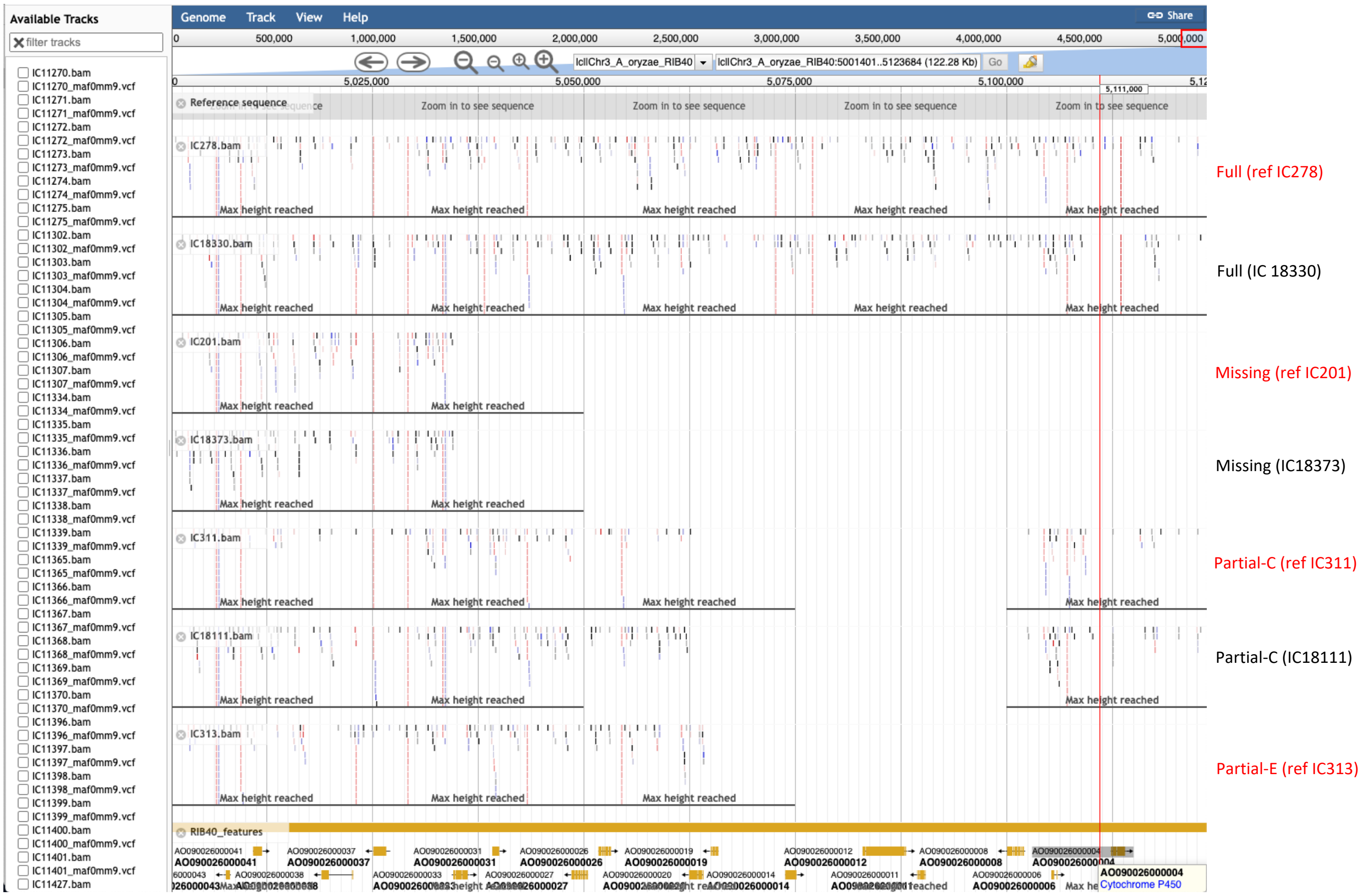

D. Identification of cluster breakpoints in Drott *et al* 2020

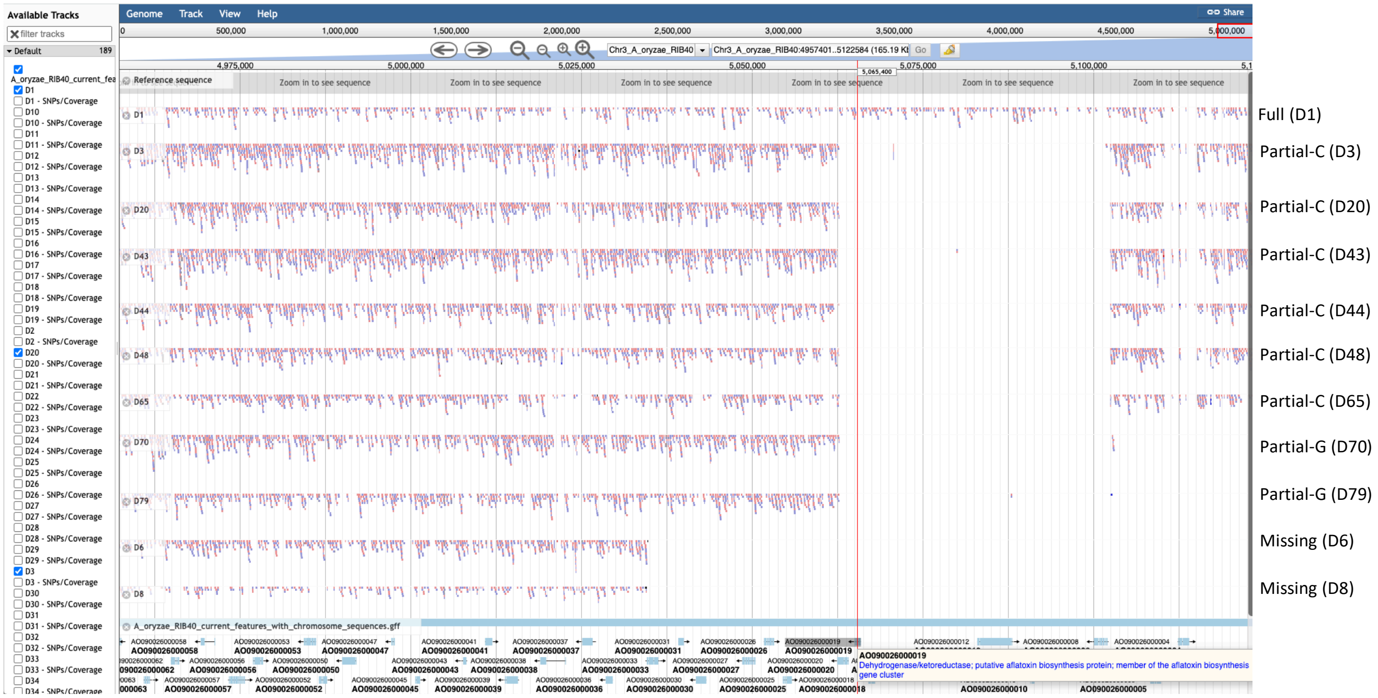

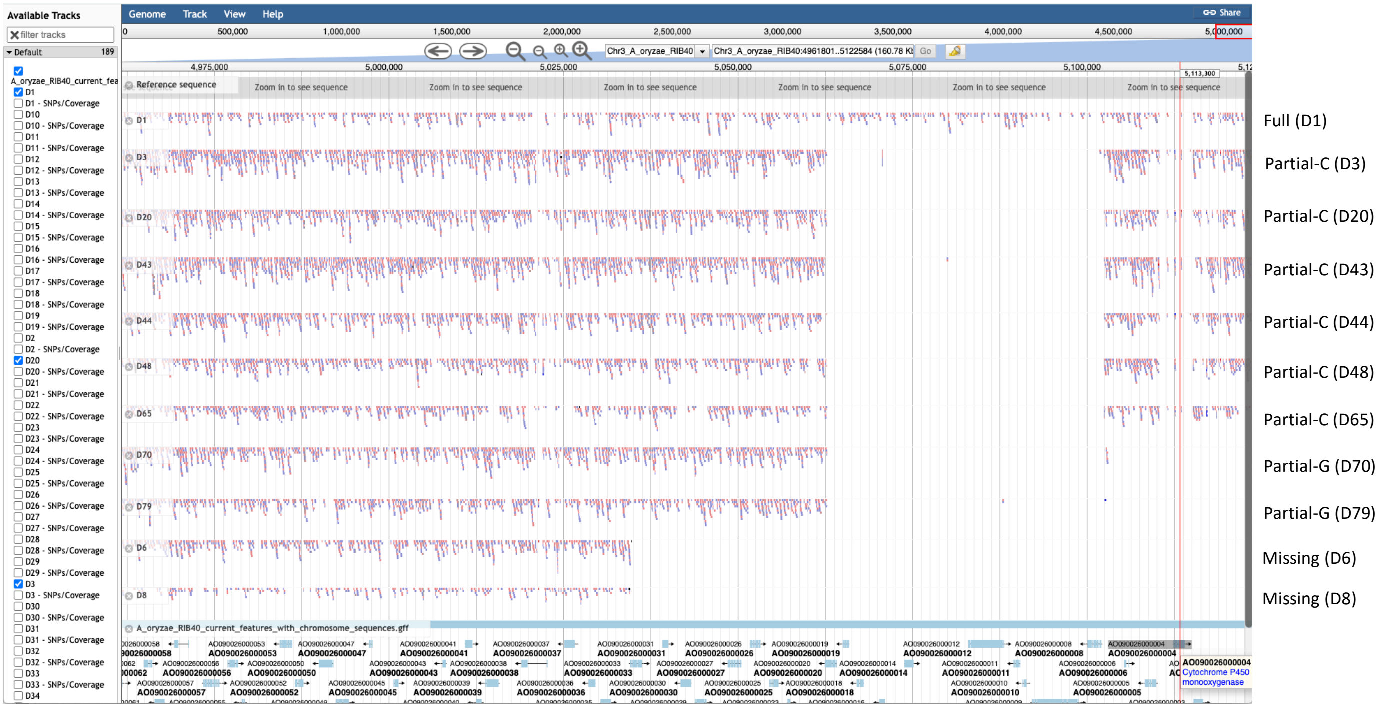

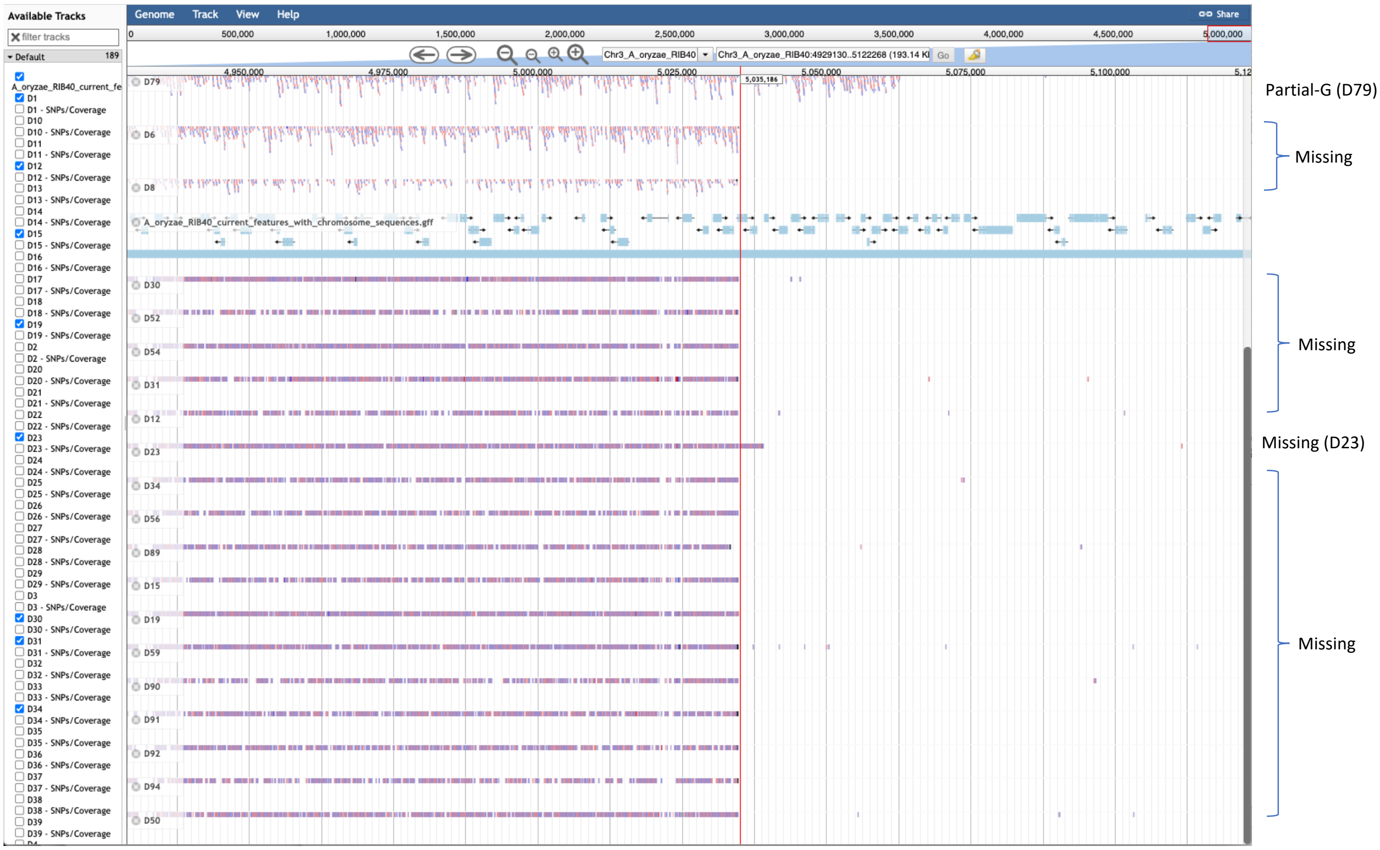

Available Tracks
filter tracks
Default 189
Genome
Track
View
Help
Share
Chr3_A_oryzae_RIB40:4929130..5122268 (193.14 Kb)
Go
5,035,186
D79
D6
D8
A_oryzae_RIB40_current_features_with_chromosome_sequences.gff
D30
D52
D54
D31
D12
D23
D34
D56
D89
D15
D19
D59
D90
D91
D92
D94
D50
Partial-G (D79)
Missing
Missing
Missing (D23)
Missing
