# Supplementary material for: Asymmetrical lineage introgression and recombination in populations of Aspergillus flavus: Implications for biological control
Source: PLoS One. 2022 Oct 27;17(10):e0276556. doi: 10.1371/journal.pone.0276556 (PMC9620740; doi:10.1371/journal.pone.0276556)

A. Network based on 817,774 SNPs using *A. oryzae* RIB40 reference genome

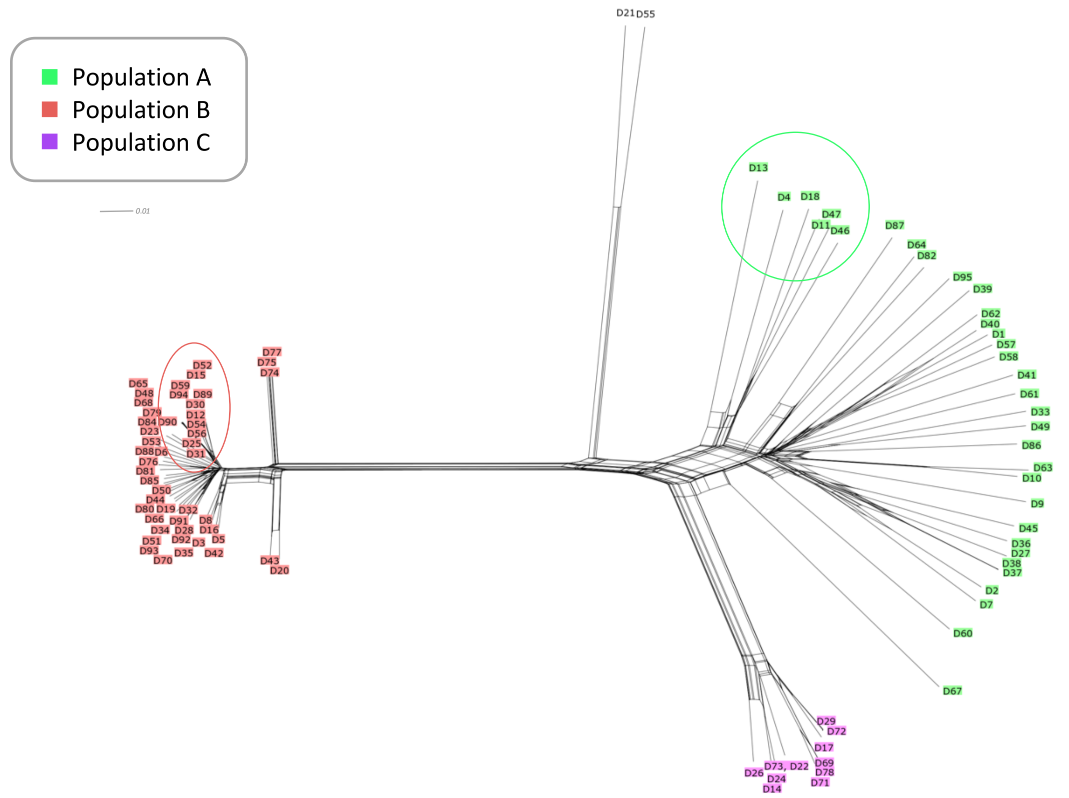

B. Network based on 5,870 SNPs from ddRADseq using *A. oryzae* RIB40 reference

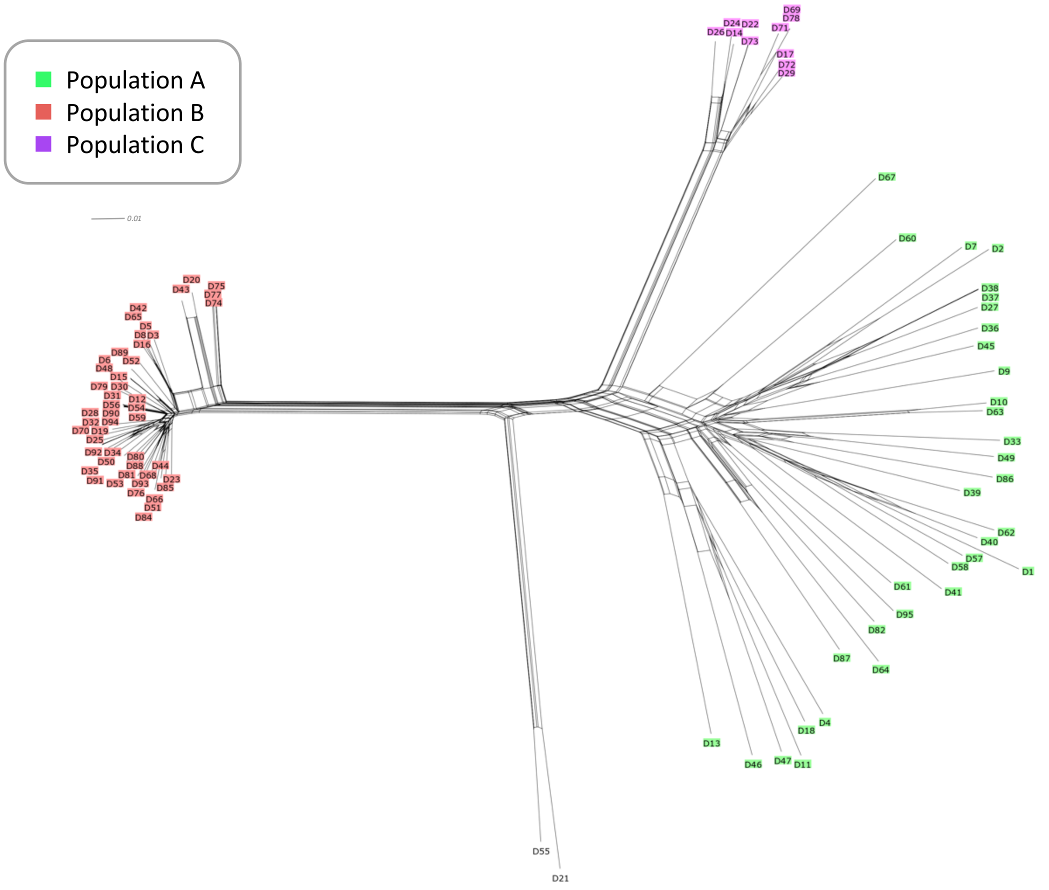

Supplement: S2 Fig — A. Network based on 817,774 SNPs using A. oryzae RIB40 reference genome showing the population structure reported in Drott et al 2020 [37]. The circles denote putative hybrid strains that were inferred from the multilocus analysis of aflM, aflW, mfs, trpC, and amdS in S4 Fig. B. Network based on 5,870 SNPs from in silico ddRADseq using A. oryzae RIB40 reference genome. The branches in the network are drawn to scale and the scale bar represents 0.01 substitutions per site. (PDF) [file pone.0276556.s002.pdf]
