# Supplementary material for: Asymmetrical lineage introgression and recombination in populations of Aspergillus flavus: Implications for biological control
Source: PLoS One. 2022 Oct 27;17(10):e0276556. doi: 10.1371/journal.pone.0276556 (PMC9620740; doi:10.1371/journal.pone.0276556)

A. Network based on 6,833 SNPs across 907 *A. flavus* isolates

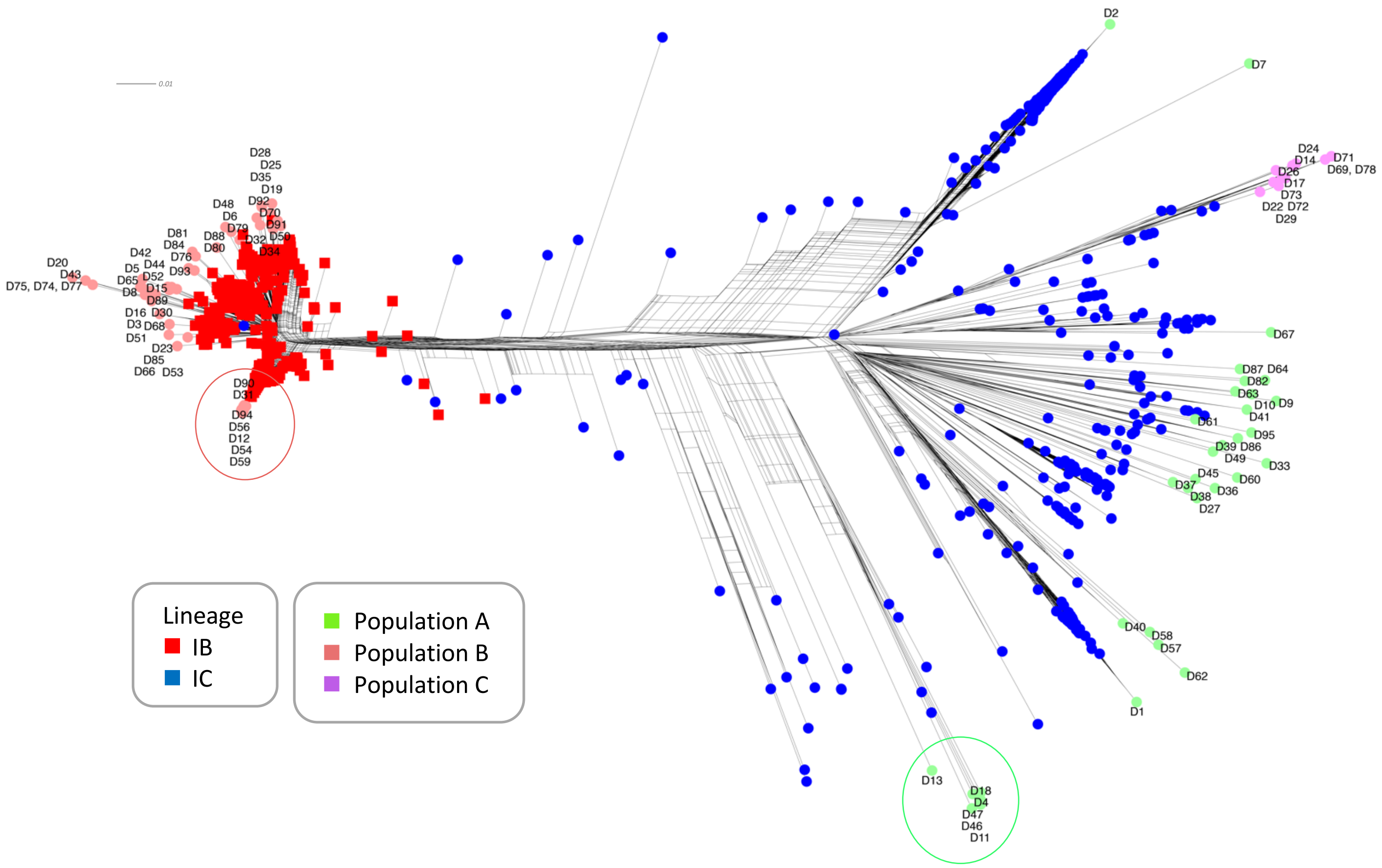

B. PCA and cluster analysis assigns all isolates into one of two distinct evolutionary lineages

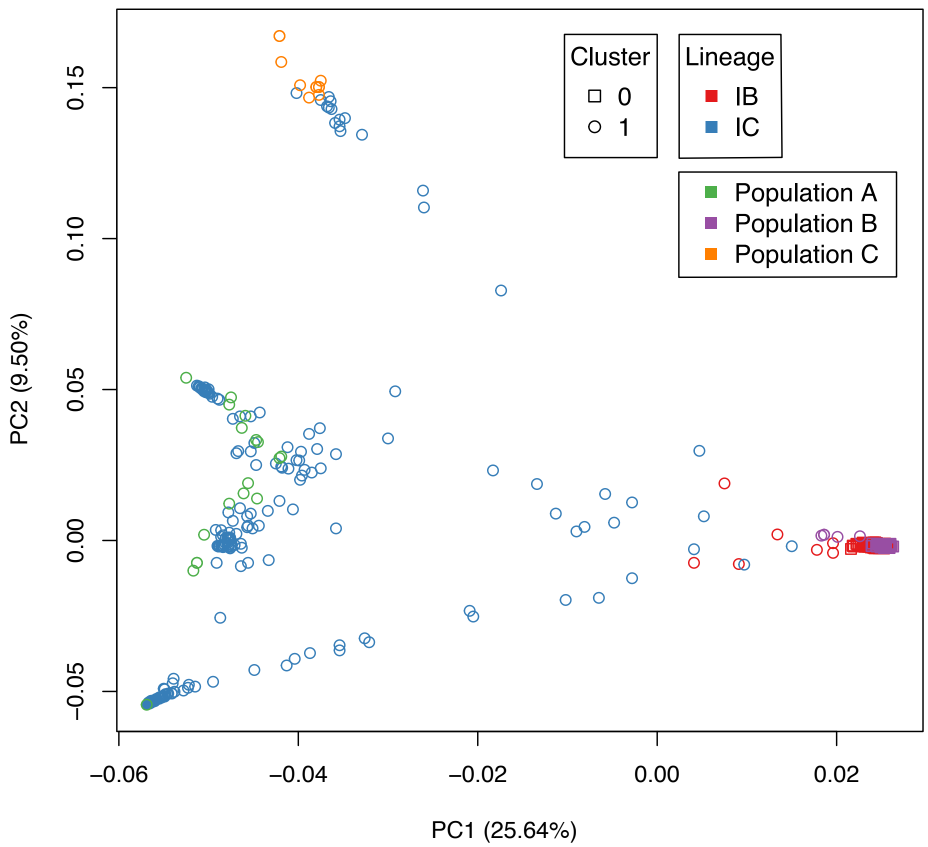

Supplement: S3 Fig — A. Network based on 6,833 SNPs across 907 A. flavus isolates. Strain names are provided only for the isolates from Drott et al 2020 [37] (indicated with a “D” prefix). Population A from Drott et al 2020 [37] is clearly subdivided and population C is nested in lineage IC with other isolates from the present study; population B falls exclusively in lineage IB. The circles denote putative hybrid strains that were inferred from the multilocus analysis of aflM, aflW, mfs, trpC, and amdS in S4 Fig. The branches in the network are drawn to scale and the scale bar represents 0.01 substitutions per site. B. PCA and cluster analysis assigns all isolates into one of two distinct evolutionary lineages: IB and IC. (PDF) [file pone.0276556.s003.pdf]
