# Supplementary material for: Asymmetrical lineage introgression and recombination in populations of Aspergillus flavus: Implications for biological control
Source: PLoS One. 2022 Oct 27;17(10):e0276556. doi: 10.1371/journal.pone.0276556 (PMC9620740; doi:10.1371/journal.pone.0276556)

A. Lineage structure based on multilocus analysis of *aflM, aflW, mfs, trpC,* and *amdS*

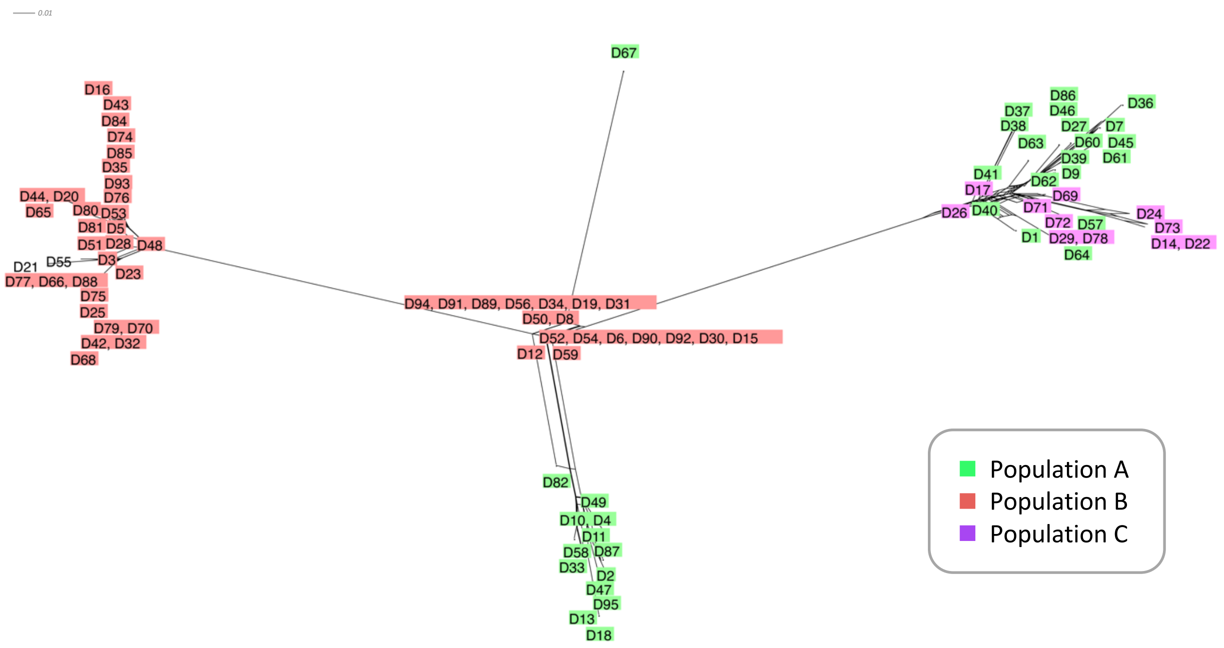

B. Distribution of missing, partial, and full aflatoxin gene clusters

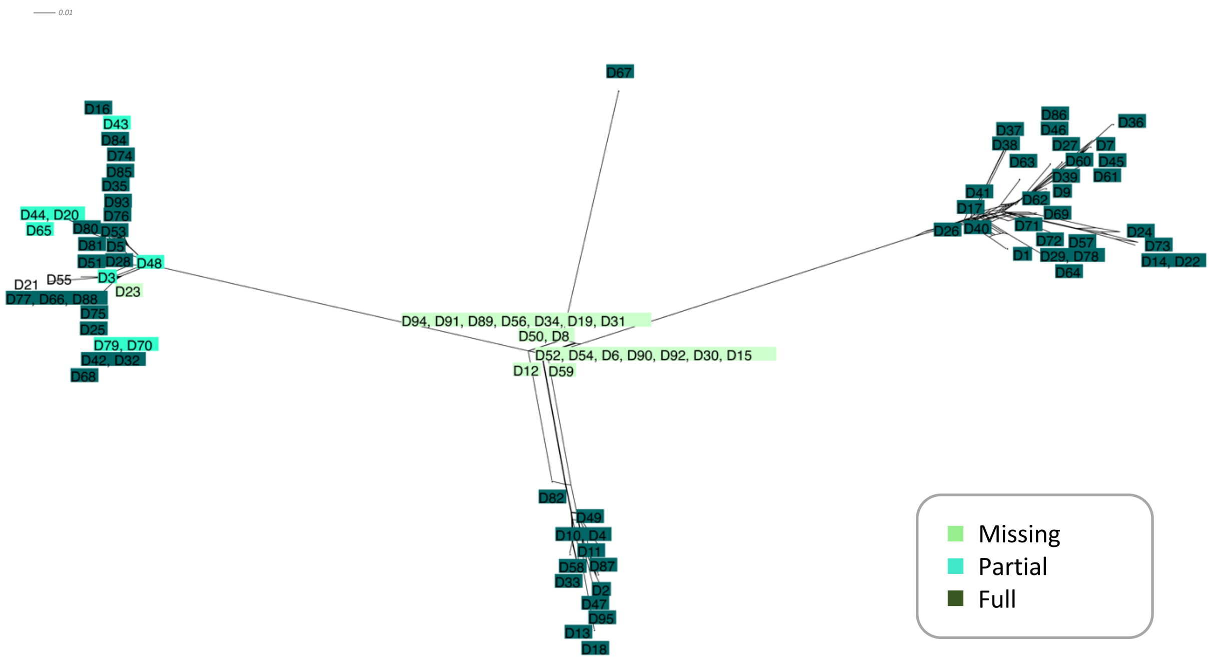

Supplement: S4 Fig — A. Lineage structure based on multilocus analysis of aflM, aflW, mfs, trpC, and amdS. At the extremes of the network are strains in lineages IB (left side) and IC (right side) and in the middle are putative inter-lineage hybrids. The position of these putative hybrid strains is shown in the larger genome-scale networks in S2 and S3 Figs. B. Overlay of cluster configurations on the multilocus network showing distribution of missing, partial, and full aflatoxin gene clusters. The branches in the network are drawn to scale and the scale bar represents 0.01 substitutions per site. (PDF) [file pone.0276556.s004.pdf]
