# Supplementary figures and images for: Asymmetrical lineage introgression and recombination in populations of Aspergillus flavus: Implications for biological control
Source: PLoS One. 2022 Oct 27;17(10):e0276556. doi: 10.1371/journal.pone.0276556 (PMC9620740; doi:10.1371/journal.pone.0276556)

A

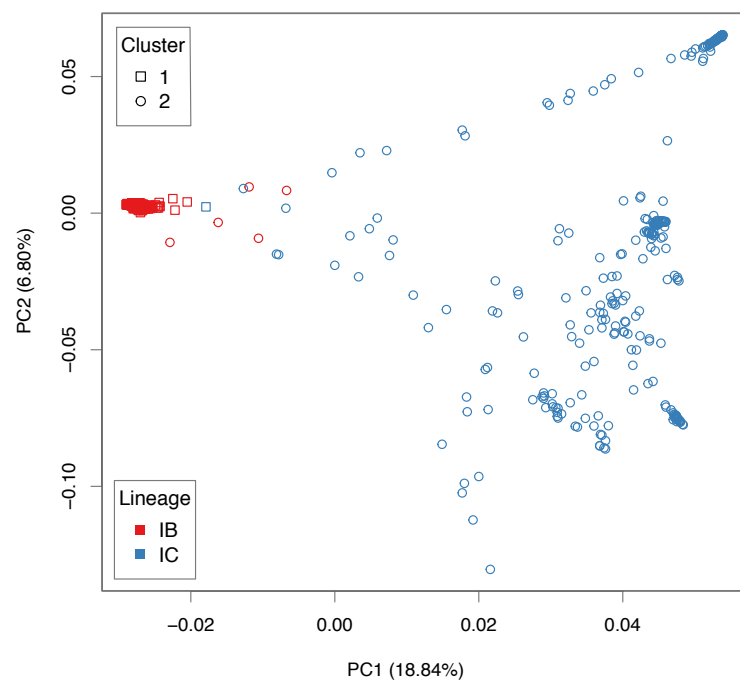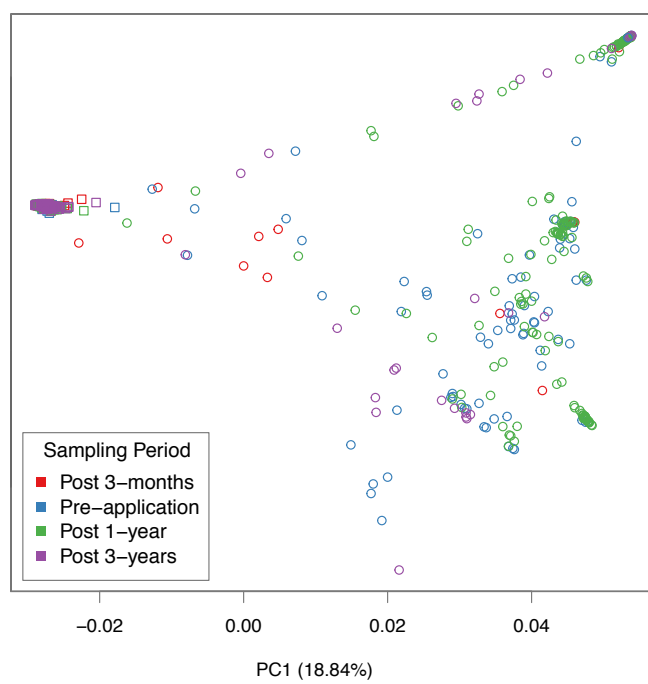

B

Pre-application

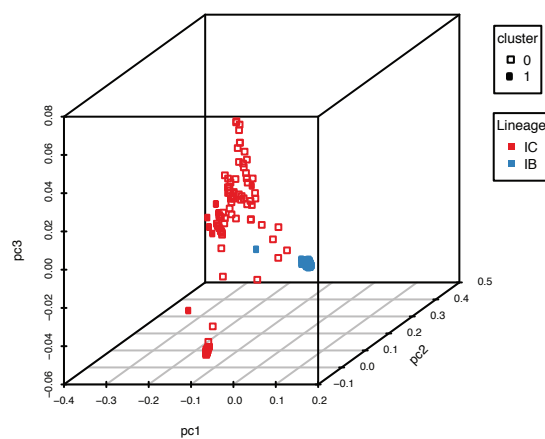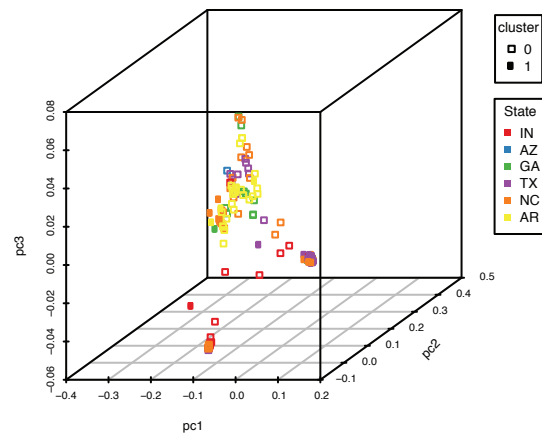

Post 3-months

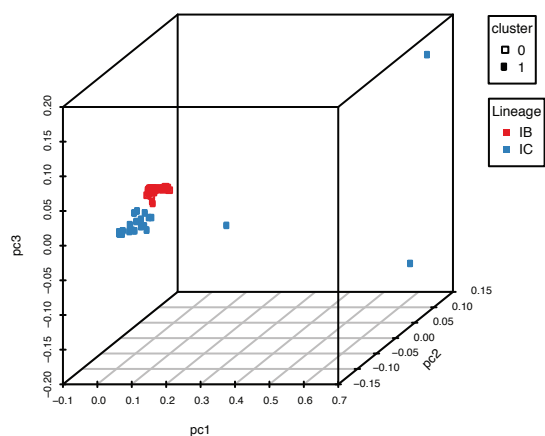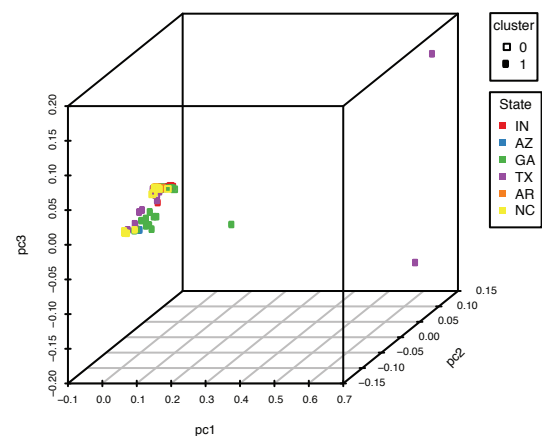

Post 1-year

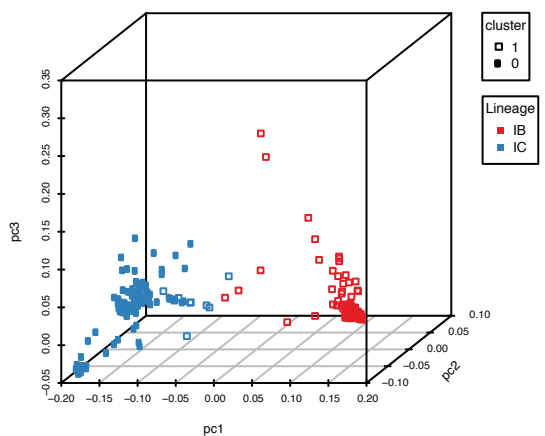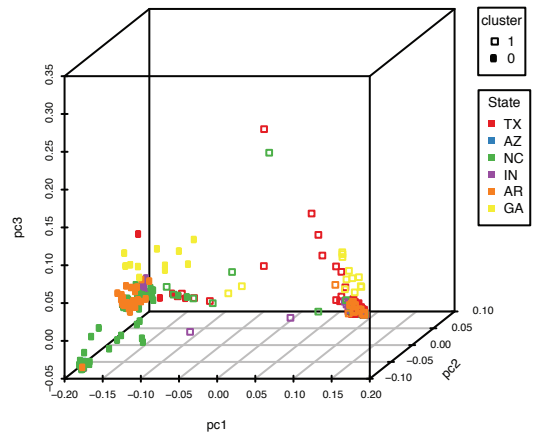

Post 3-years

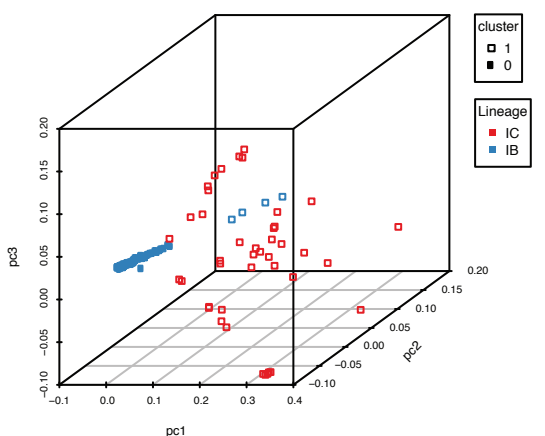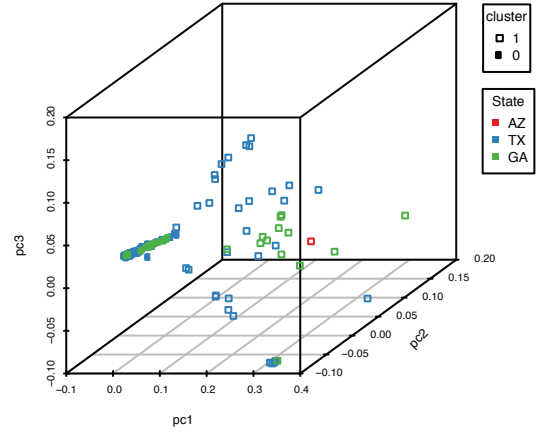

Supplement: S6 Fig — A. PCA scatter plots for 815 A. flavus isolates showing clusters, lineages and sampling period for the networks shown in Fig 1. B. For each treatment time point two PCA scatter plots are shown for genome-wide variation in A. flavus across TX, NC, AR and IN (reference strains are from GA and AZ). The PCA cubes show the distribution of individuals based on their membership in one of two clusters inferred from the Gap statistic and overlaid with lineage (PCA cube on left) or state (PCA cube on right). The color scheme and shapes are unique for each PCA cube. (PDF) [file pone.0276556.s006.pdf]

**A**

**TEXAS Commercial**

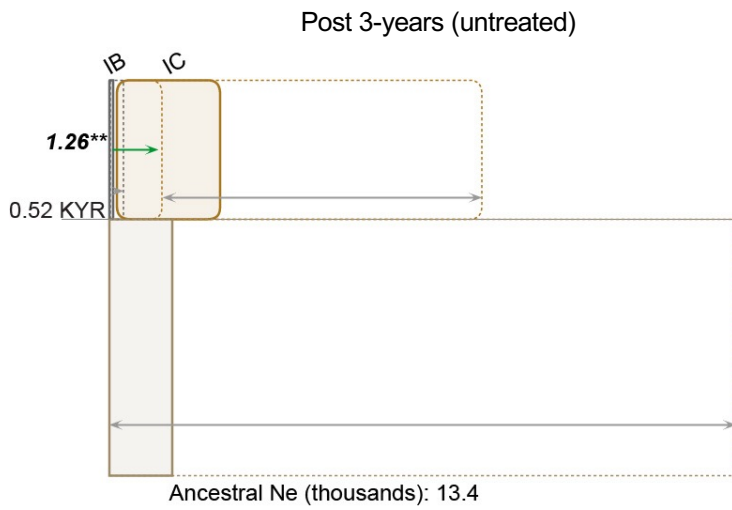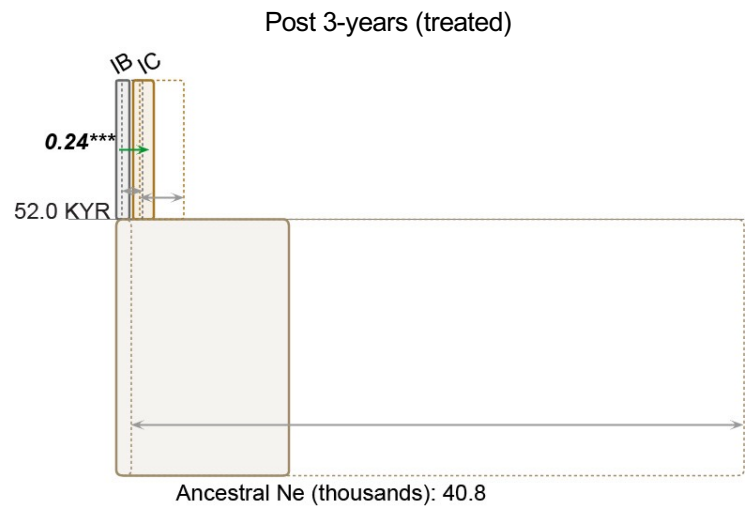

**B**

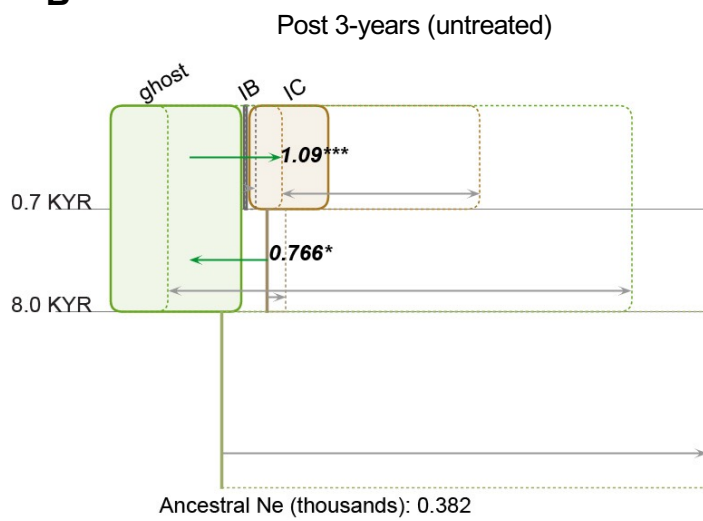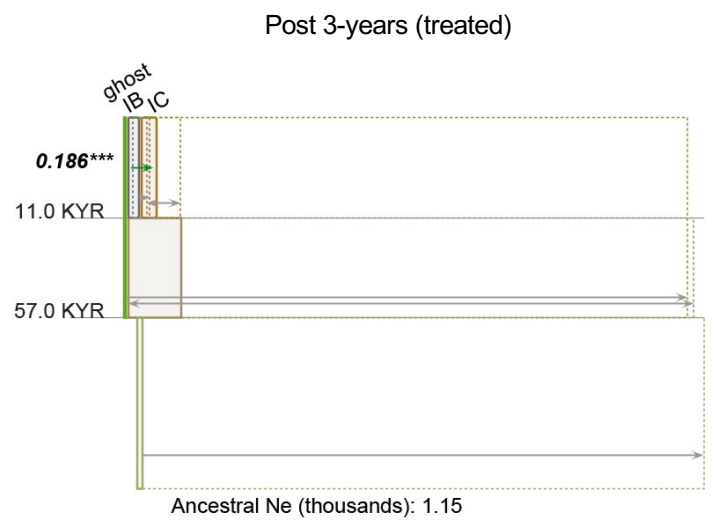

Supplement: S9 Fig — A. Without a ghost population. B. With a ghost population. The phylogeny is depicted as a hierarchical series of boxes, with ancestor boxes connecting descendant populations of lineages IB and IC, and the width of boxes proportional to the estimated Ne. The 95% confidence intervals for each Ne value are shown as dashed lines to the right of the left side of the corresponding population box. Gray arrows to the 95% Ne intervals extend on either side of the right side of each population box. Splitting times, positioned at even intervals, are depicted as solid horizontal lines, with text values on the left in units of thousand years ago (KYA). Migration arrows (in green) indicate the estimated population migration rate (Nem) values from one population into another from when the populations diverged from a common ancestor. Arrows are shown only for migration rates that are statistically significant (* p < 0.05, ** p < 0.01, *** p < 0.001). Estimates assumed a generation time of 0.17 years and a mutation rate of 4.2 × 10^−11^ per base per generation. (PDF) [file pone.0276556.s009.pdf]

# Pre-application

Total

Chr1

Chr2

Chr3

Chr4

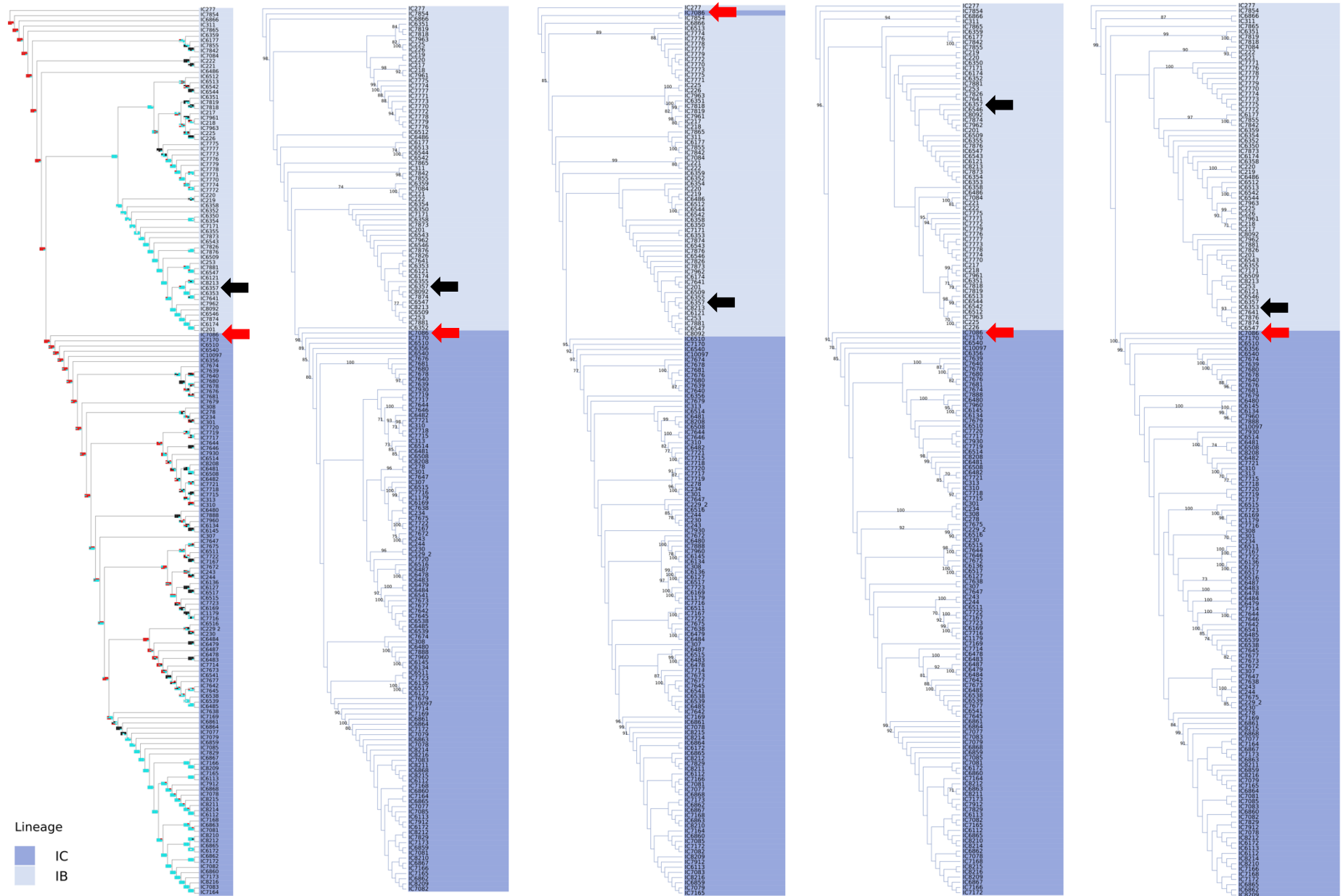

Chr5

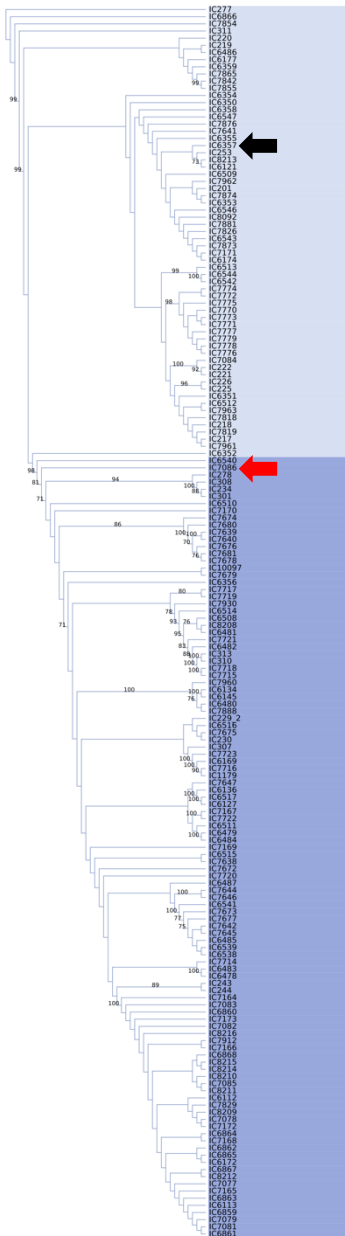

Chr6

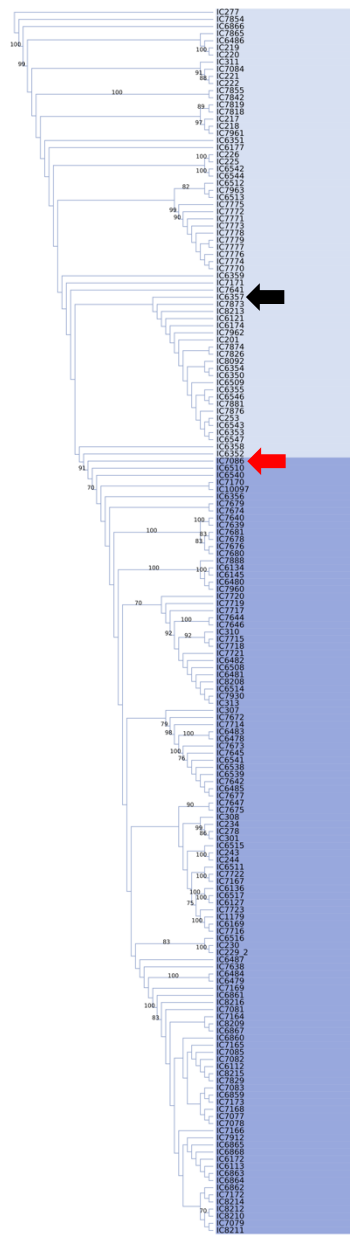

Chr7

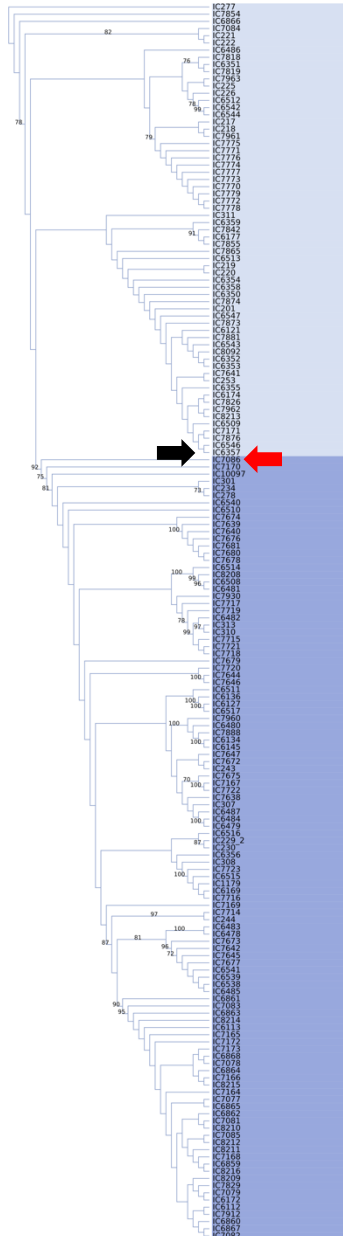

Chr8

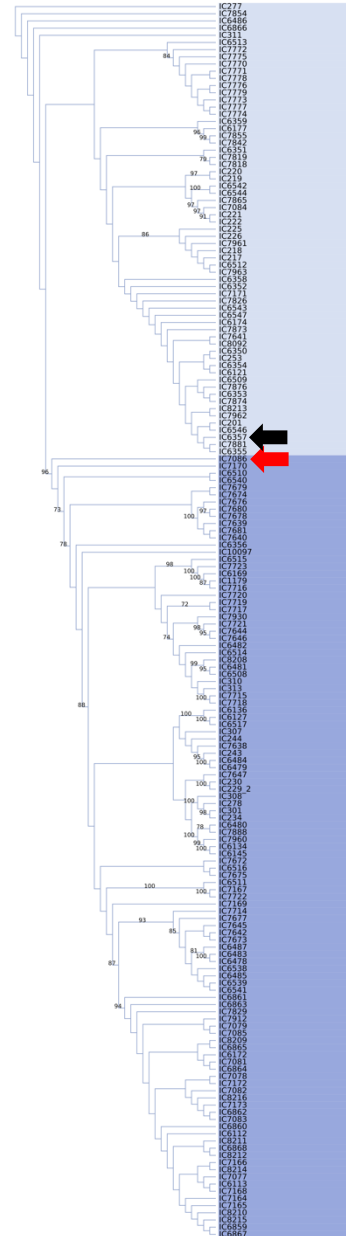

Mito

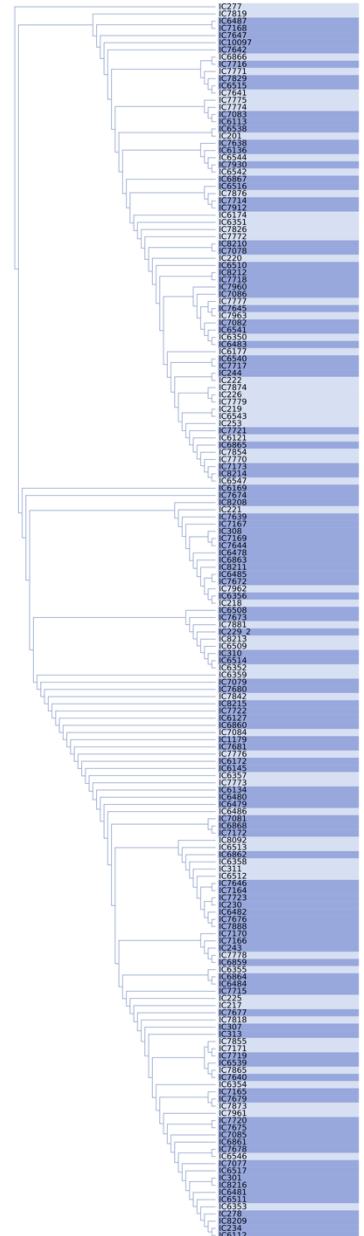

Supplement: S11 Fig — In the total evidence display tree colors in grids represent node bipartitions that were supported at a bootstrap support value ≥70% (black color), <70% (white color), and missing or inapplicable (grey color). Phylogenetic incongruency was represented as high conflict (red color) and low conflict (cyan color). Strain names are highlighted to show their lineage membership in the total evidence tree; the mitochondrial genome has insufficient variation and poor resolution of lineage structure. The red arrows track the position of strain IC7086 sampled from IN which belongs to lineage IC; the black arrows track the position of IC6357 sampled from TX which is in lineage IB. Strain IC7086 is grouping with lineage IB strains in chromosome 2 but is placed in lineage IC on all other chromosomes with strong bootstrap support (≥90%). Strain IC6357 groups only with lineage IB strains on different chromosomes but with weak bootstrap support (<70%). (PDF) [file pone.0276556.s011.pdf]

# Post 3-months

Total

Chr1

Chr2

Chr3

Chr4

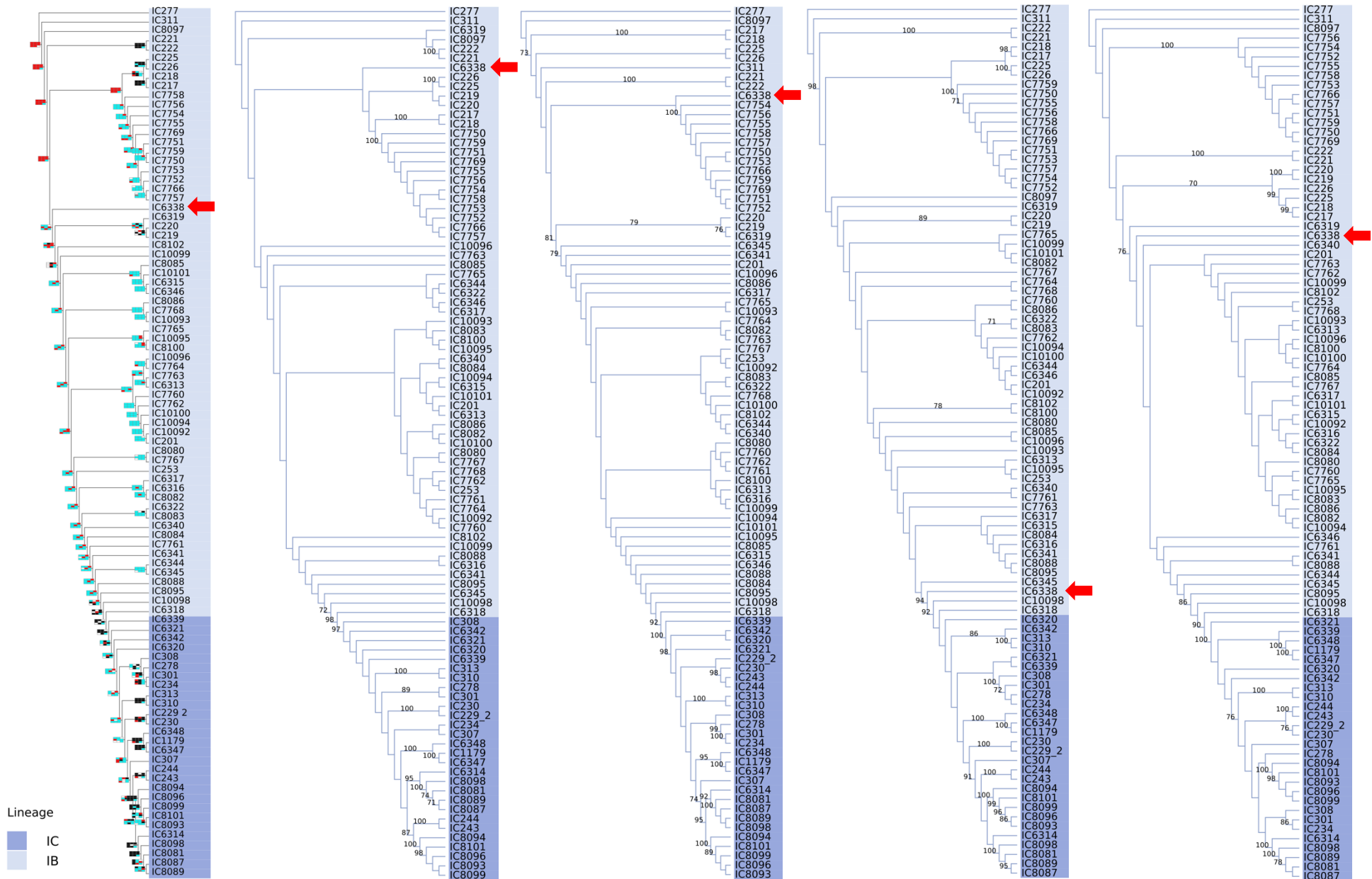

Chr5

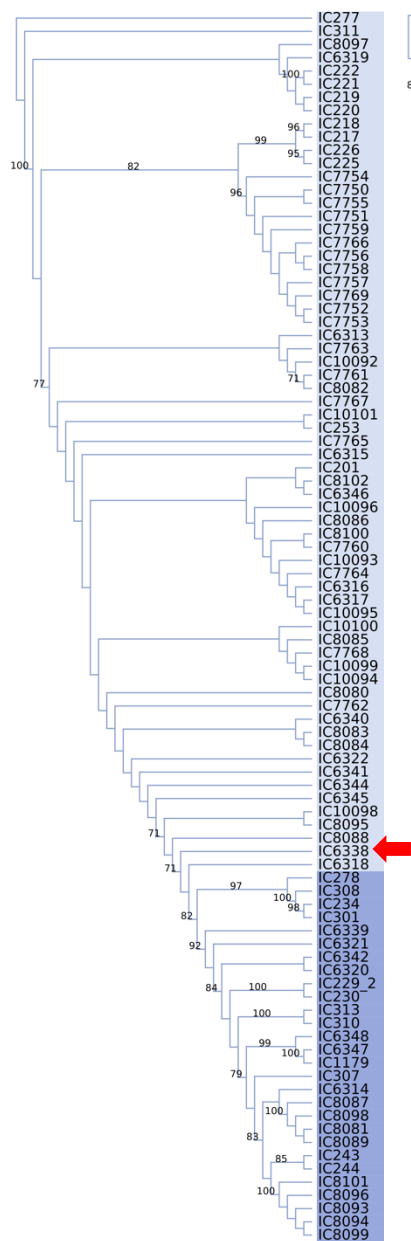

Chr6

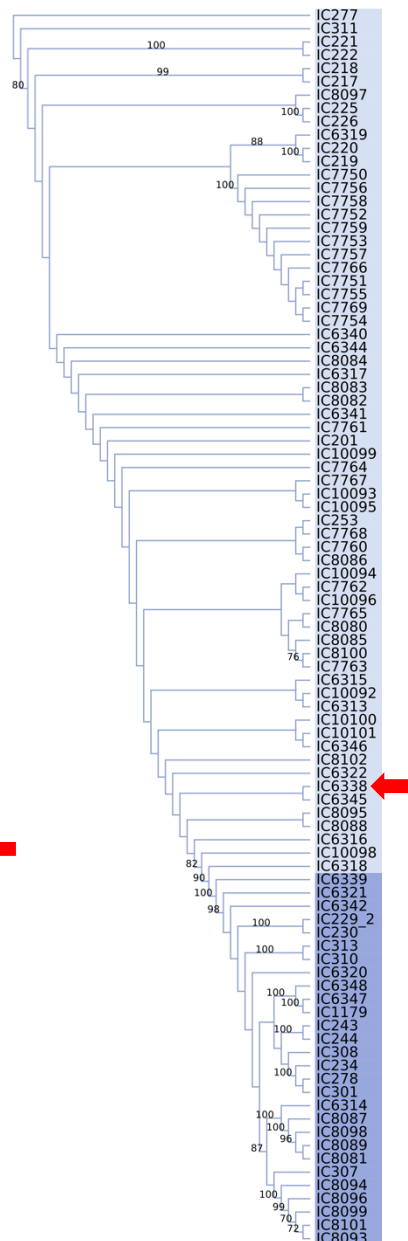

Chr7

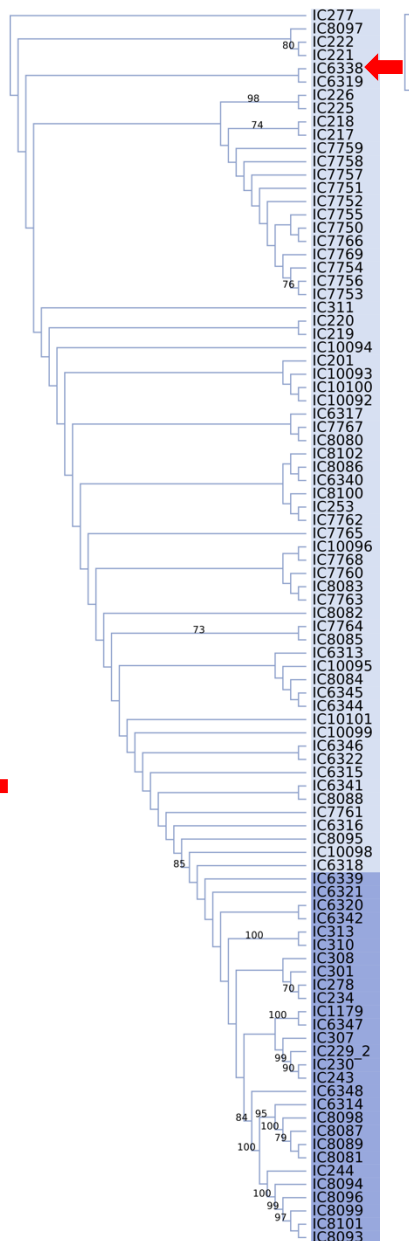

Chr8

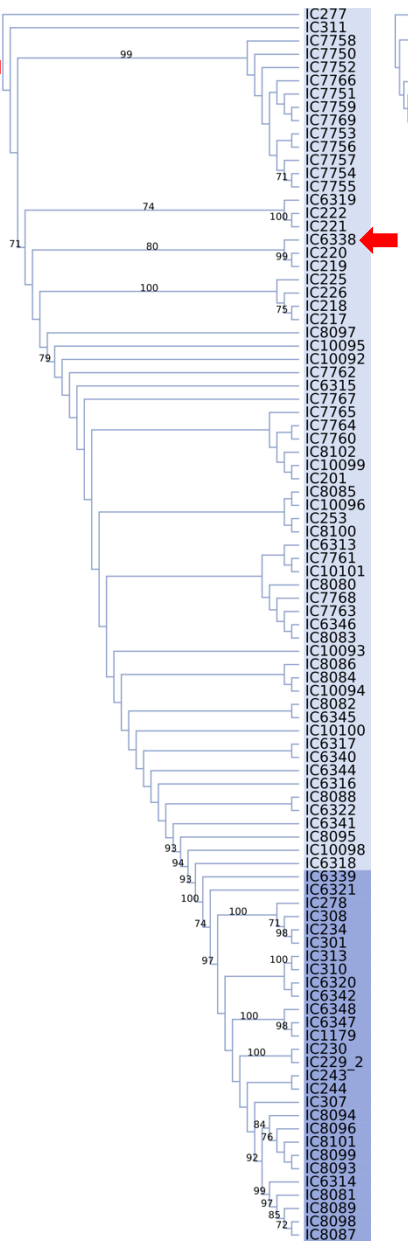

Mito

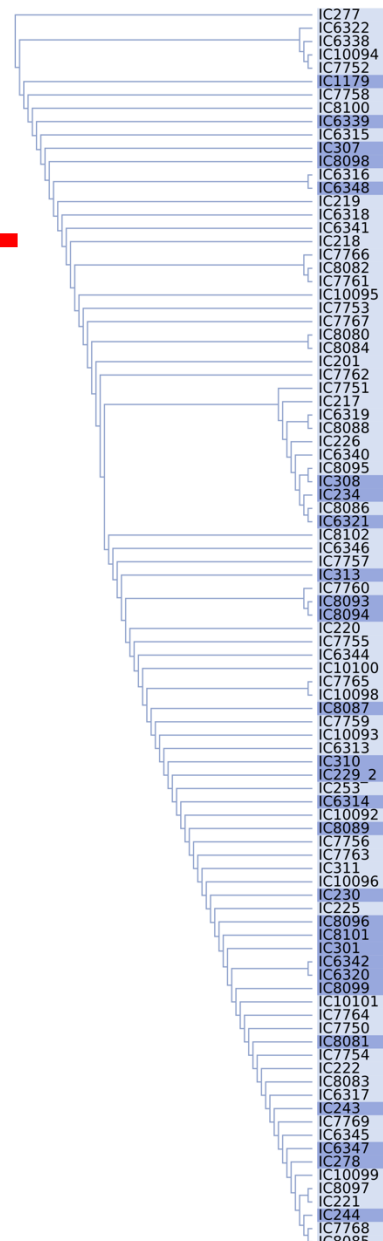

Supplement: S12 Fig — In the total evidence display tree colors in grids represent node bipartitions that were supported at a bootstrap support value ≥70% (black color), <70% (white color), and missing or inapplicable (grey color). Phylogenetic incongruency was represented as high conflict (red color) and low conflict (cyan color). Strain names are highlighted to show their lineage membership in the total evidence tree; the mitochondrial genome has insufficient variation and poor resolution of lineage structure. The red arrows track the position of strain IC6338 sampled from TX which belongs to lineage IB in the total evidence tree. Strain IC6338 groups in lineage IB in chromosomes 1, 2, 4, 6, 7 and 8, but there is strong bootstrap support (>70%) for IC6338 grouping with strains in lineage IC in chromosomes 3 and 5. (PDF) [file pone.0276556.s012.pdf]

Post 1-year

Total

Chr1

Chr2

Chr3

Chr4

Lineage  
IC  
IB

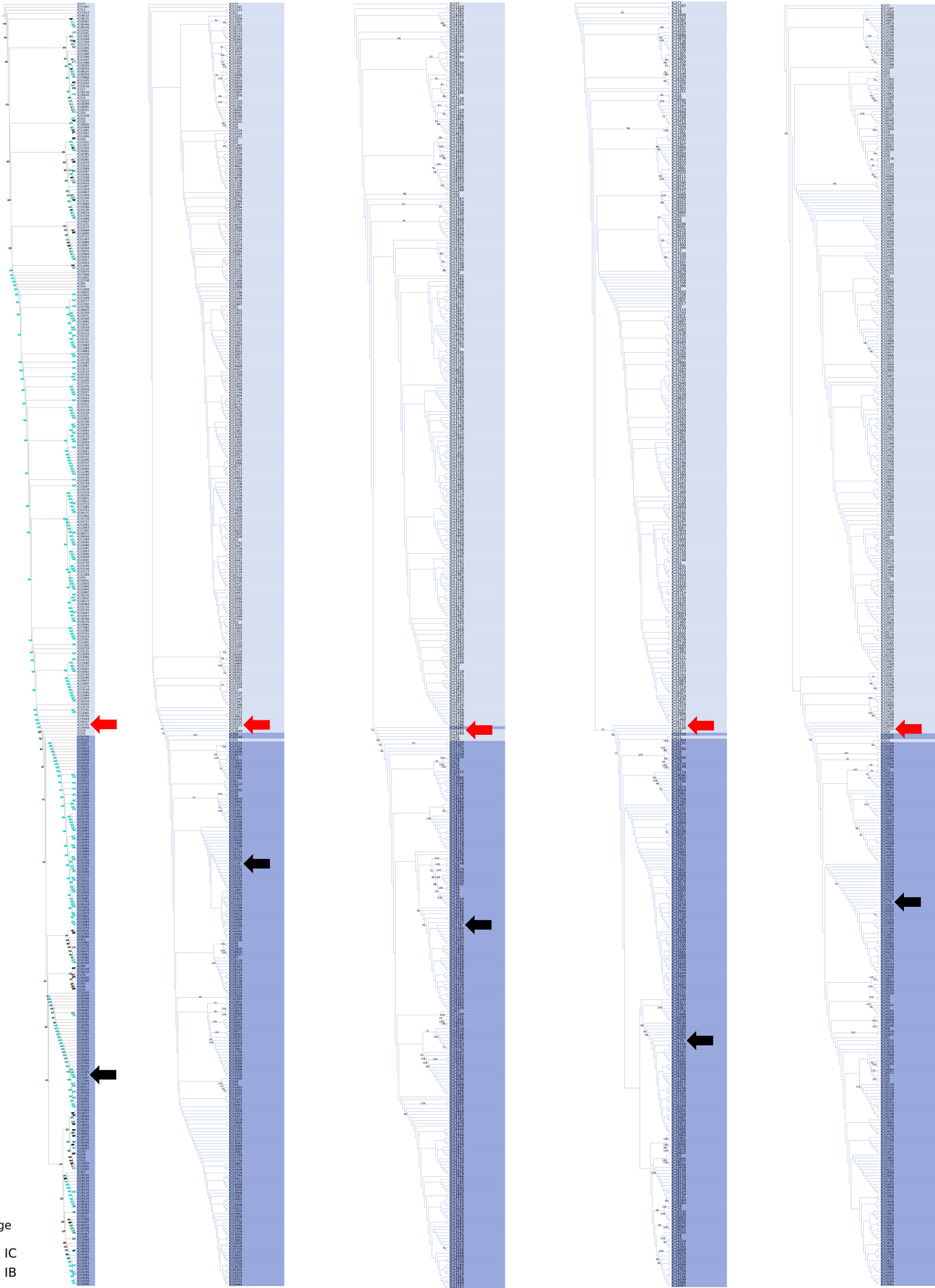

Chr5

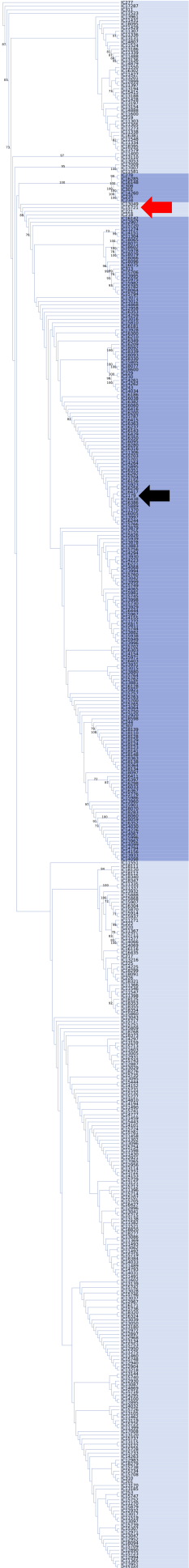

Chr6

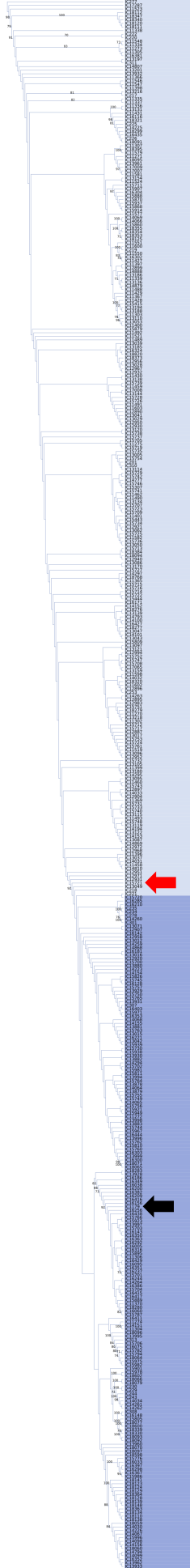

Chr7

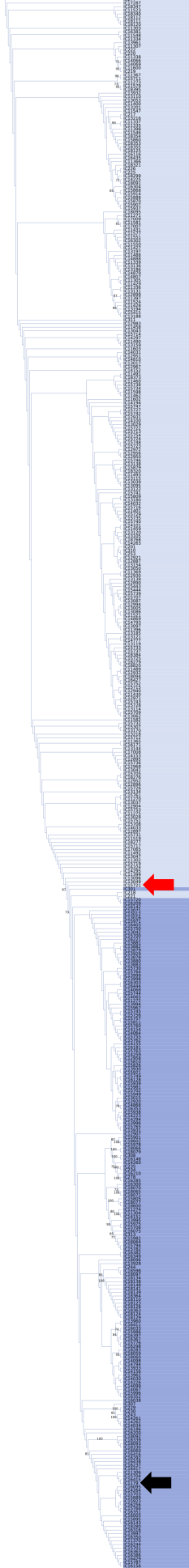

Chr8

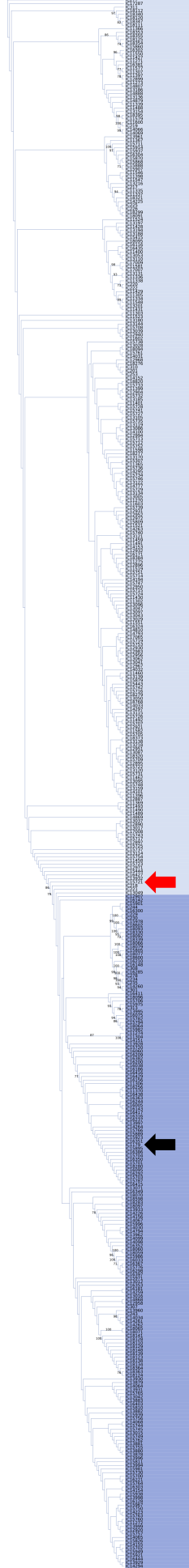

Mito

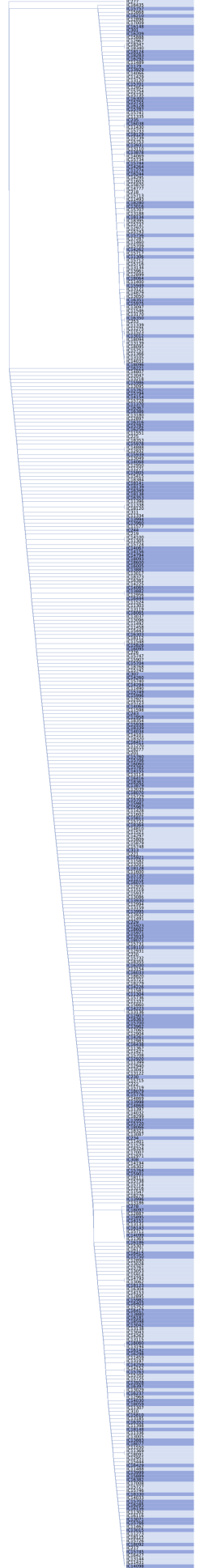

Supplement: S13 Fig — In the total evidence display tree colors in grids represent node bipartitions that were supported at a bootstrap support value ≥70% (black color), <70% (white color), and missing or inapplicable (grey color). Phylogenetic incongruency was represented as high conflict (red color) and low conflict (cyan color). Strain names are highlighted to show their lineage membership in the total evidence tree; the mitochondrial genome has insufficient variation and poor resolution of lineage structure. The red arrows track the position of strain IC15721 sampled from IN which belongs to lineage IB in the total evidence tree but sharing recent common ancestry with strains in both lineages across most chromosomes making lineage assignment difficult. The black arrows track the position of the AF36 biocontrol strain, IC1179, which belongs to lineage IC and has low phylogenetic conflict with other stains in that lineage suggesting a history of recombination. (PDF) [file pone.0276556.s013.pdf]

# Post 3-years

Total

Chr1

Chr2

Chr3

Chr4

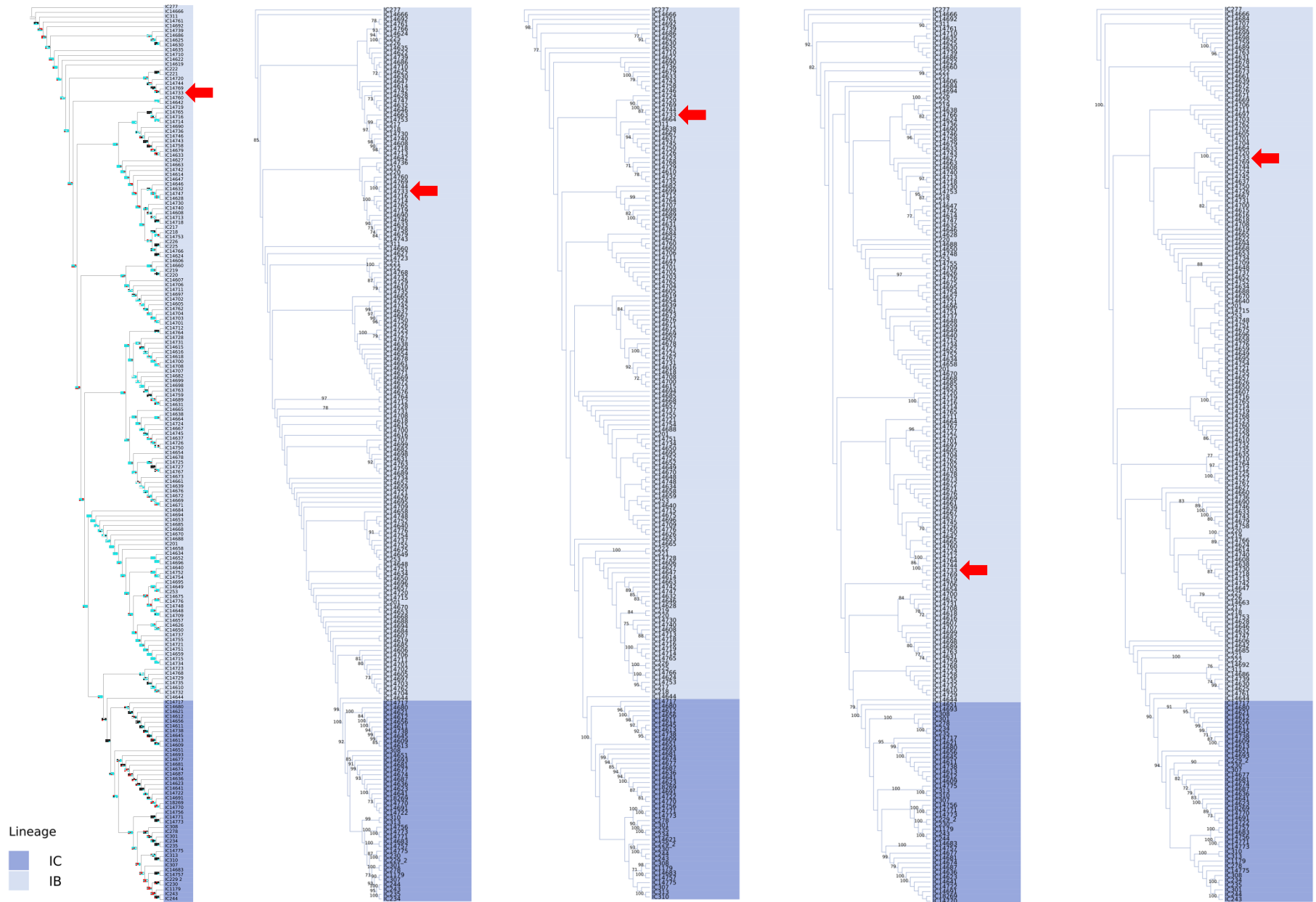

Chr5

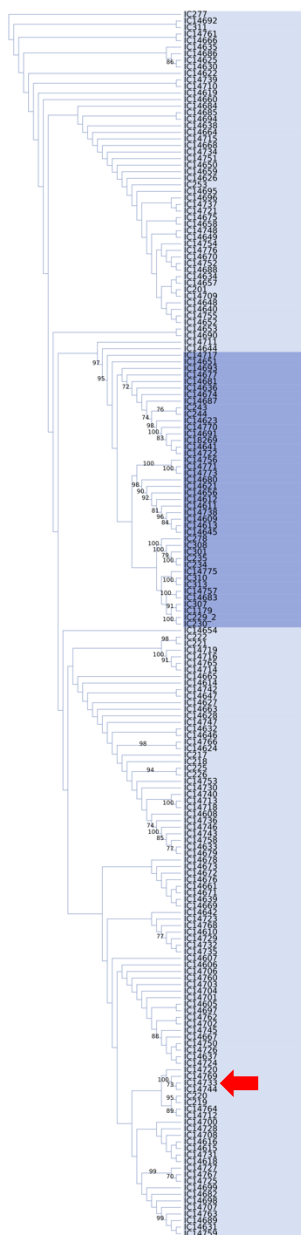

Chr6

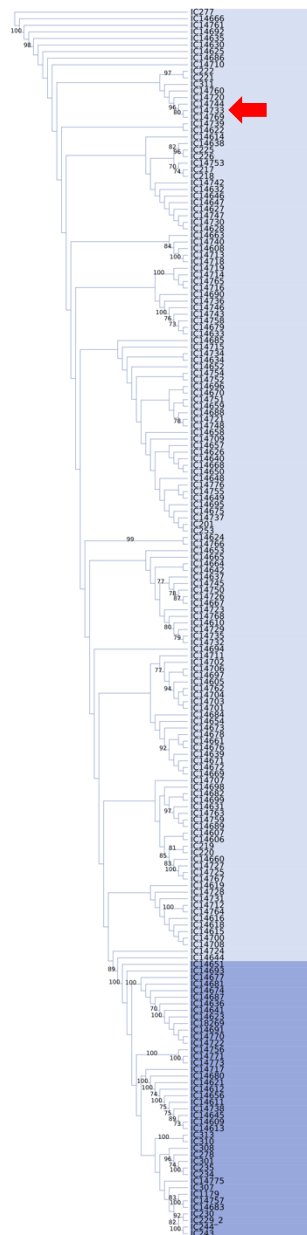

Chr7

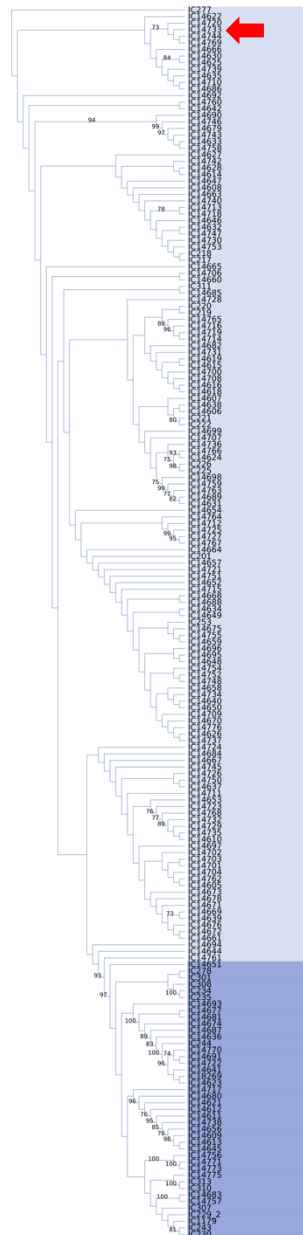

Chr8

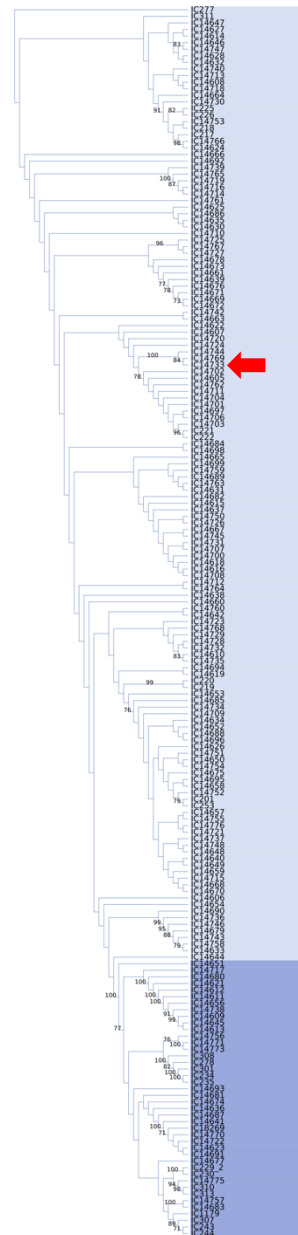

Mito

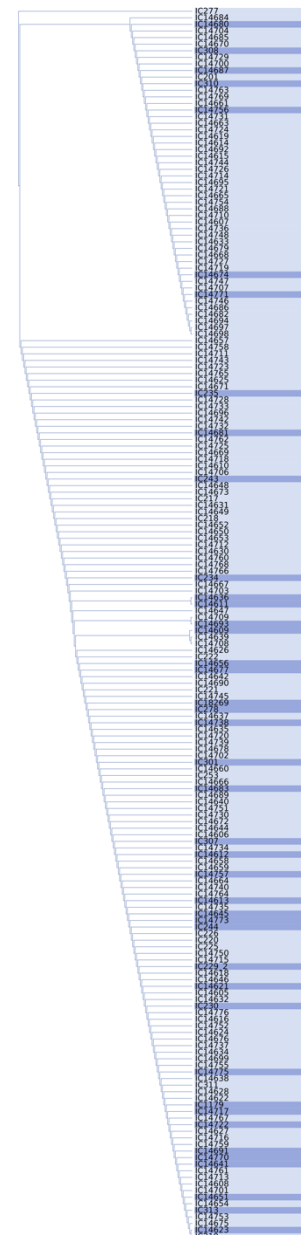

Supplement: S14 Fig — In the total evidence display tree colors in grids represent node bipartitions that were supported at a bootstrap support value ≥70% (black color), <70% (white color), and missing or inapplicable (grey color). Phylogenetic incongruency was represented as high conflict (red color) and low conflict (cyan color). Strain names are highlighted to show their lineage membership in the total evidence tree; the mitochondrial genome has insufficient variation and poor resolution of lineage structure. The red arrows track the position of strain IC14733 which belongs to lineage IB and shares very recent common ancestry with IC14744 and IC14769 with strong bootstrap support in chromosomes 1, 3, 5, 6 and 8; however, IC14733, IC14744 and IC14769 are also grouping with IC14720 with strong bootstrap support in chromosomes 2, 4, and 7. (PDF) [file pone.0276556.s014.pdf]
