# Supplementary material for: Asymmetrical lineage introgression and recombination in populations of Aspergillus flavus: Implications for biological control
Source: PLoS One. 2022 Oct 27;17(10):e0276556. doi: 10.1371/journal.pone.0276556 (PMC9620740; doi:10.1371/journal.pone.0276556)

TEXAS

Pre-application (untreated)

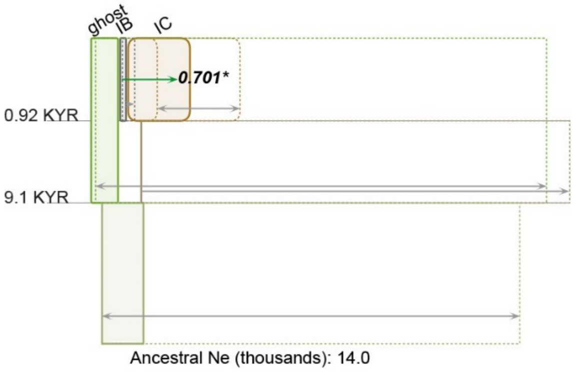

Post 1-year (untreated)

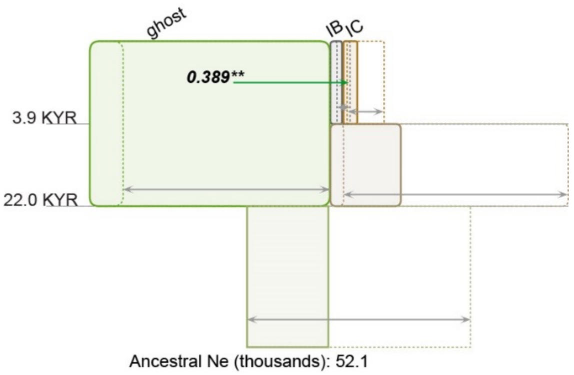

Post 3-months (treated)

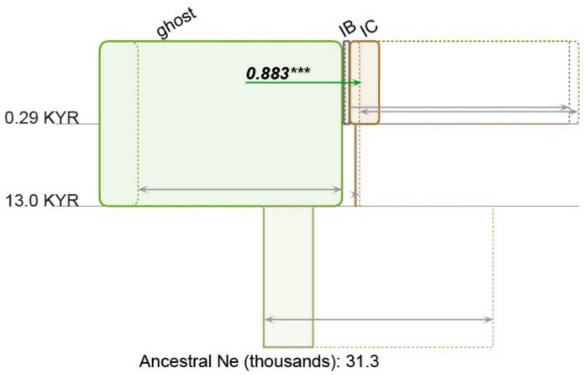

Post 1-year (treated)

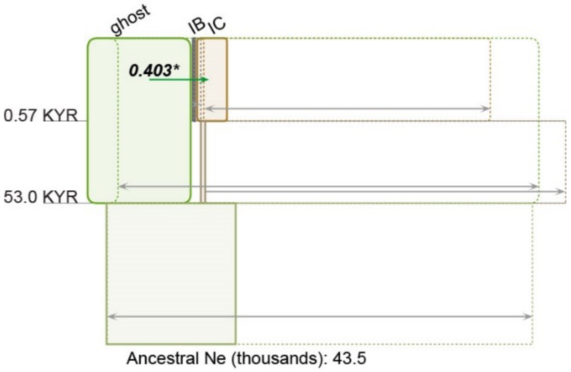

NORTH CAROLINA

Pre-application (untreated)

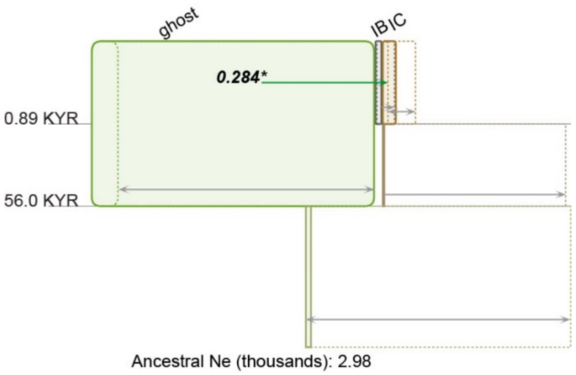

Post 1-year (untreated)

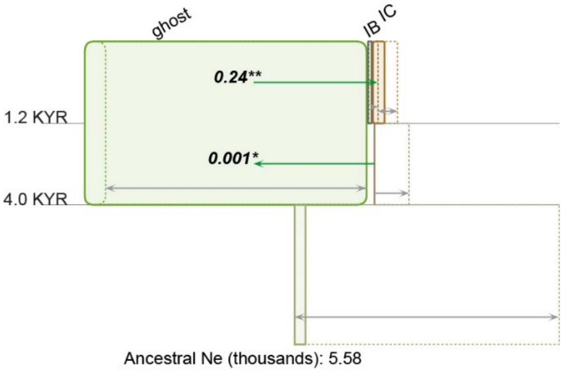

Post 3-months (treated)

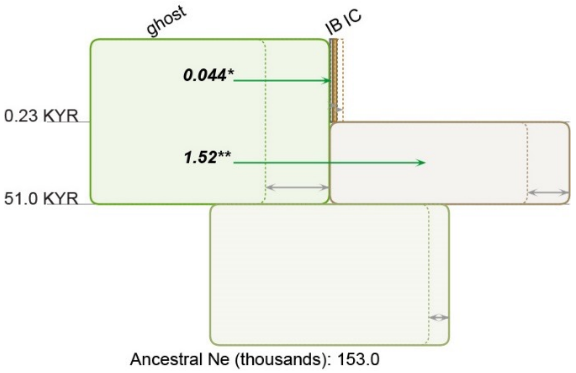

Post 1-year (treated)

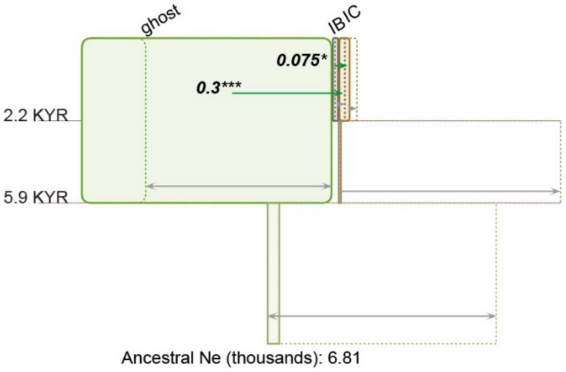

Supplement: S7 Fig — The phylogeny is depicted as a hierarchical series of boxes, with ancestor boxes connecting descendant populations of lineages IB and IC, and the width of boxes proportional to the estimated Ne. The 95% confidence intervals for each Ne value are shown as dashed lines to the right of the left side of the corresponding population box. Gray arrows to the 95% Ne intervals extend on either side of the right side of each population box. Splitting times, positioned at even intervals, are depicted as solid horizontal lines, with text values on the left in units of thousand years ago (KYA). Migration arrows (in green) indicate the estimated population migration rate (Nem) values from one population into another from when the populations diverged from a common ancestor. Arrows are shown only for migration rates that are statistically significant (* p < 0.05, ** p < 0.01, *** p < 0.001). Estimates assumed a generation time of 0.17 years and a mutation rate of 4.2 × 10^−11^ per base per generation. (PDF) [file pone.0276556.s007.pdf]
