# Supplementary material for: Asymmetrical lineage introgression and recombination in populations of Aspergillus flavus: Implications for biological control
Source: PLoS One. 2022 Oct 27;17(10):e0276556. doi: 10.1371/journal.pone.0276556 (PMC9620740; doi:10.1371/journal.pone.0276556)
Supplement: S8 Fig — A. Without a ghost population. B. With a ghost population. The phylogeny is depicted as a hierarchical series of boxes, with ancestor boxes connecting descendant populations of lineages IB and IC, and the width of boxes proportional to the estimated Ne. The 95% confidence intervals for each Ne value are shown as dashed lines to the right of the left side of the corresponding population box. Gray arrows to the 95% Ne intervals extend on either side of the right side of each population box. Splitting times, positioned at even intervals, are depicted as solid horizontal lines, with text values on the left in units of thousand years ago (KYA). Migration arrows (in green) indicate the estimated population migration rate (Nem) values from one population into another from when the populations diverged from a common ancestor. Arrows are shown only for migration rates that are statistically significant (* p < 0.05, ** p < 0.01, *** p < 0.001). Estimates assumed a generation time of 0.17 years and a mutation rate of 4.2 × 10^−11^ per base per generation. (PDF) [file pone.0276556.s008.pdf]

A

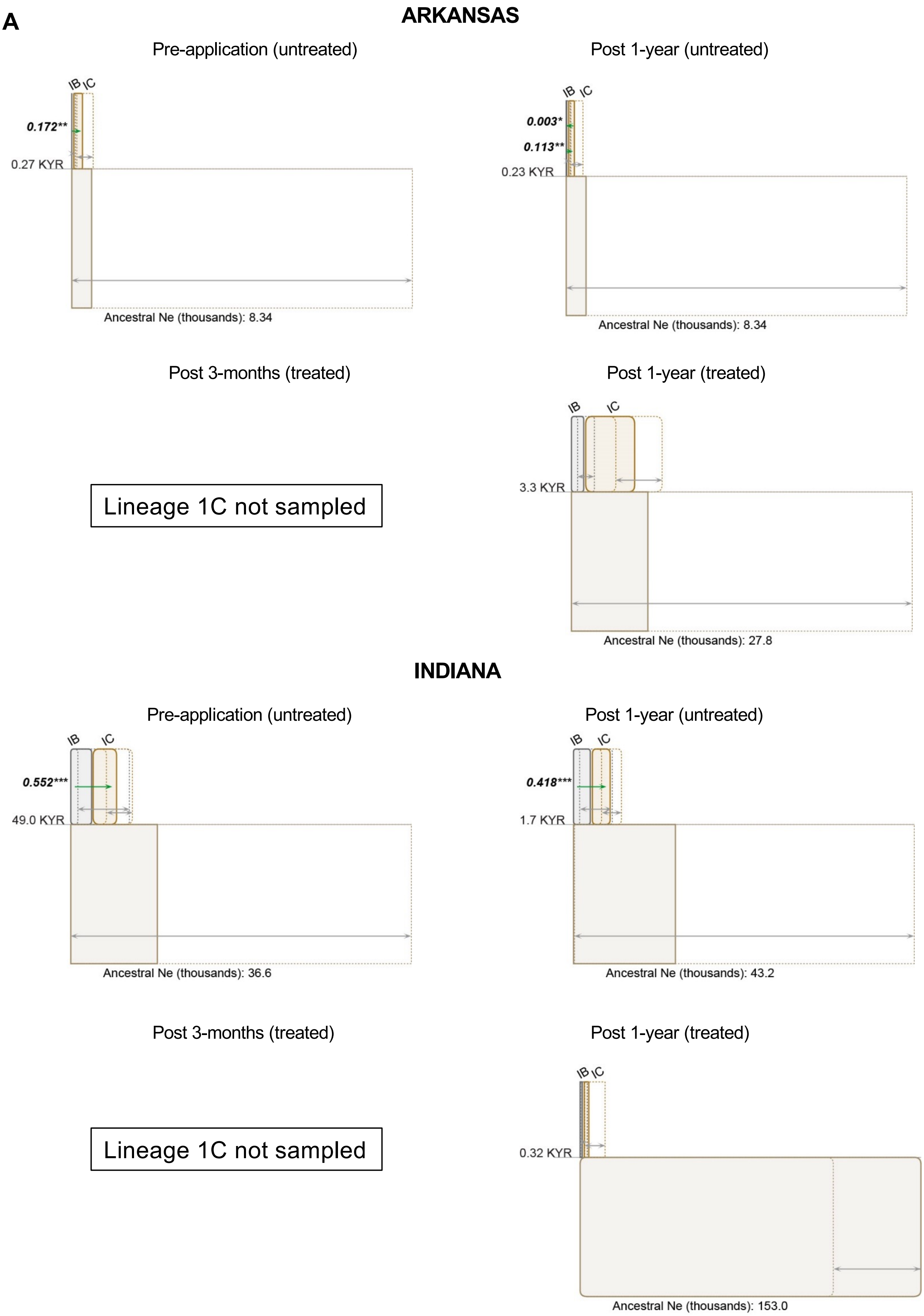
ARKANSAS
Pre-application (untreated)
IB
IC
0.172**
0.27 KYR
Ancestral Ne (thousands): 8.34
Post 1-year (untreated)
IB
IC
0.003*
0.113**
0.23 KYR
Ancestral Ne (thousands): 8.34
Post 3-months (treated)
Lineage 1C not sampled
Post 1-year (treated)
IB
IC
3.3 KYR
Ancestral Ne (thousands): 27.8
INDIANA
Pre-application (untreated)
IB
IC
0.552***
49.0 KYR
Ancestral Ne (thousands): 36.6
Post 1-year (untreated)
IB
IC
0.418***
1.7 KYR
Ancestral Ne (thousands): 43.2
Post 3-months (treated)
Lineage 1C not sampled
Post 1-year (treated)
IB
IC
0.32 KYR
Ancestral Ne (thousands): 153.0

B

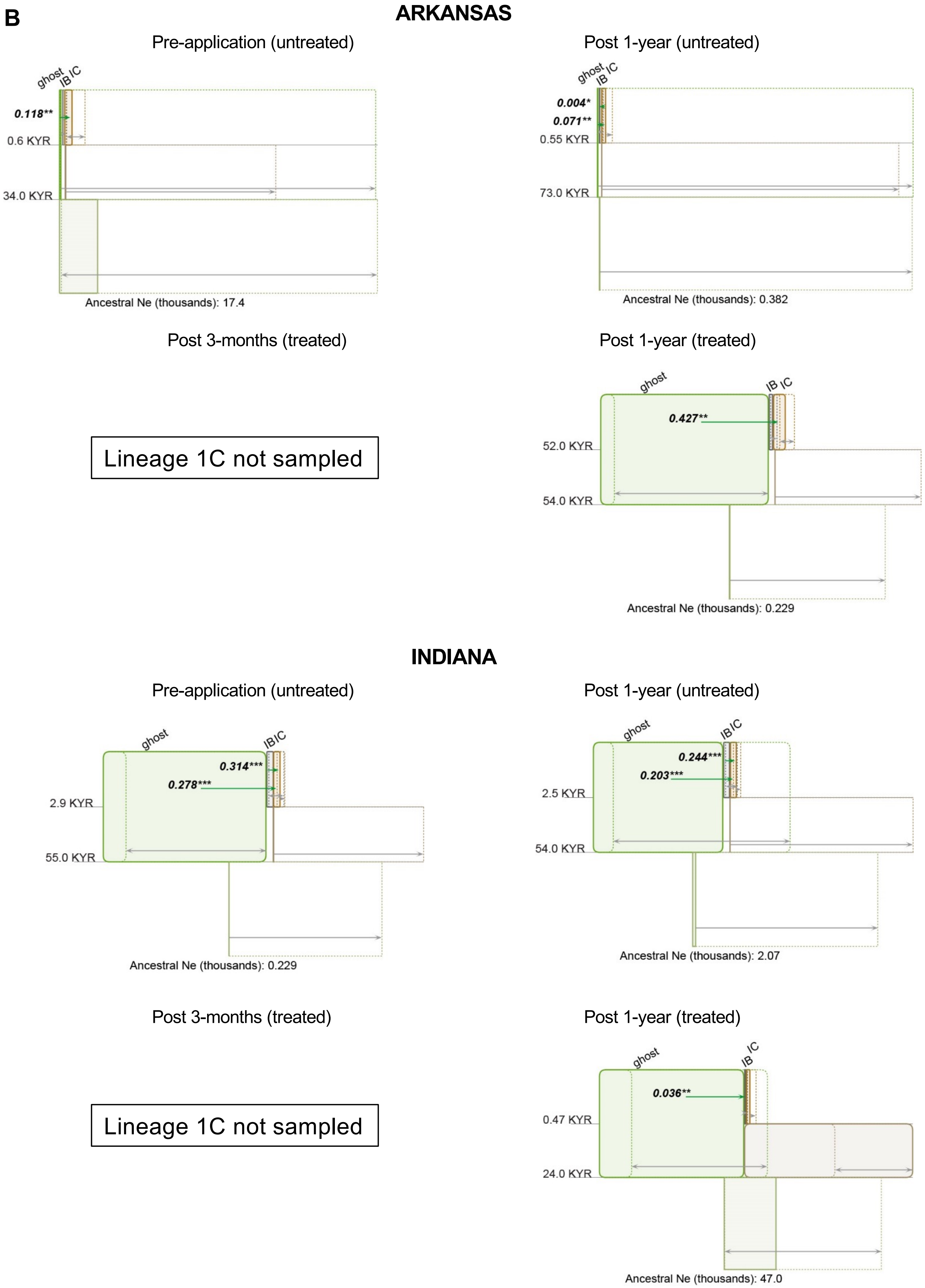
ARKANSAS
Pre-application (untreated)
ghost
IB
IC
0.118**
0.6 KYR
34.0 KYR
Ancestral Ne (thousands): 17.4
Post 1-year (untreated)
ghost
IB
IC
0.004*
0.071**
0.55 KYR
73.0 KYR
Ancestral Ne (thousands): 0.382
Post 3-months (treated)
Lineage 1C not sampled
Post 1-year (treated)
ghost
IB
IC
0.427**
52.0 KYR
54.0 KYR
Ancestral Ne (thousands): 0.229
INDIANA
Pre-application (untreated)
ghost
IB
IC
0.314***
0.278***
2.9 KYR
55.0 KYR
Ancestral Ne (thousands): 0.229
Post 1-year (untreated)
ghost
IB
IC
0.244***
0.203***
2.5 KYR
54.0 KYR
Ancestral Ne (thousands): 2.07
Post 3-months (treated)
Lineage 1C not sampled
Post 1-year (treated)
ghost
IB
IC
0.036**
0.47 KYR
24.0 KYR
Ancestral Ne (thousands): 47.0
