# Supplementary material for: Asymmetrical lineage introgression and recombination in populations of Aspergillus flavus: Implications for biological control
Source: PLoS One. 2022 Oct 27;17(10):e0276556. doi: 10.1371/journal.pone.0276556 (PMC9620740; doi:10.1371/journal.pone.0276556)

(A) Pre-application

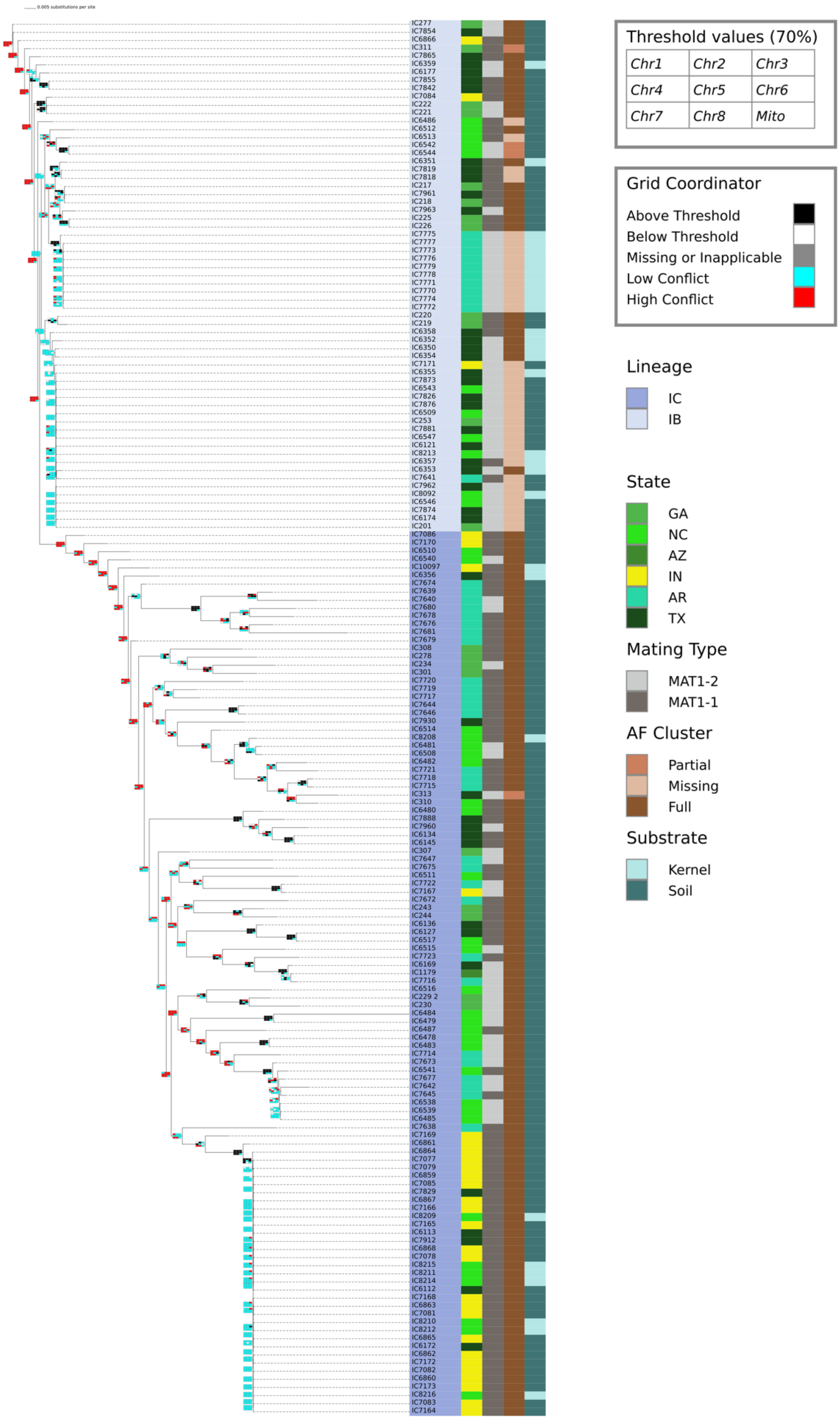

(B) Post 3-months

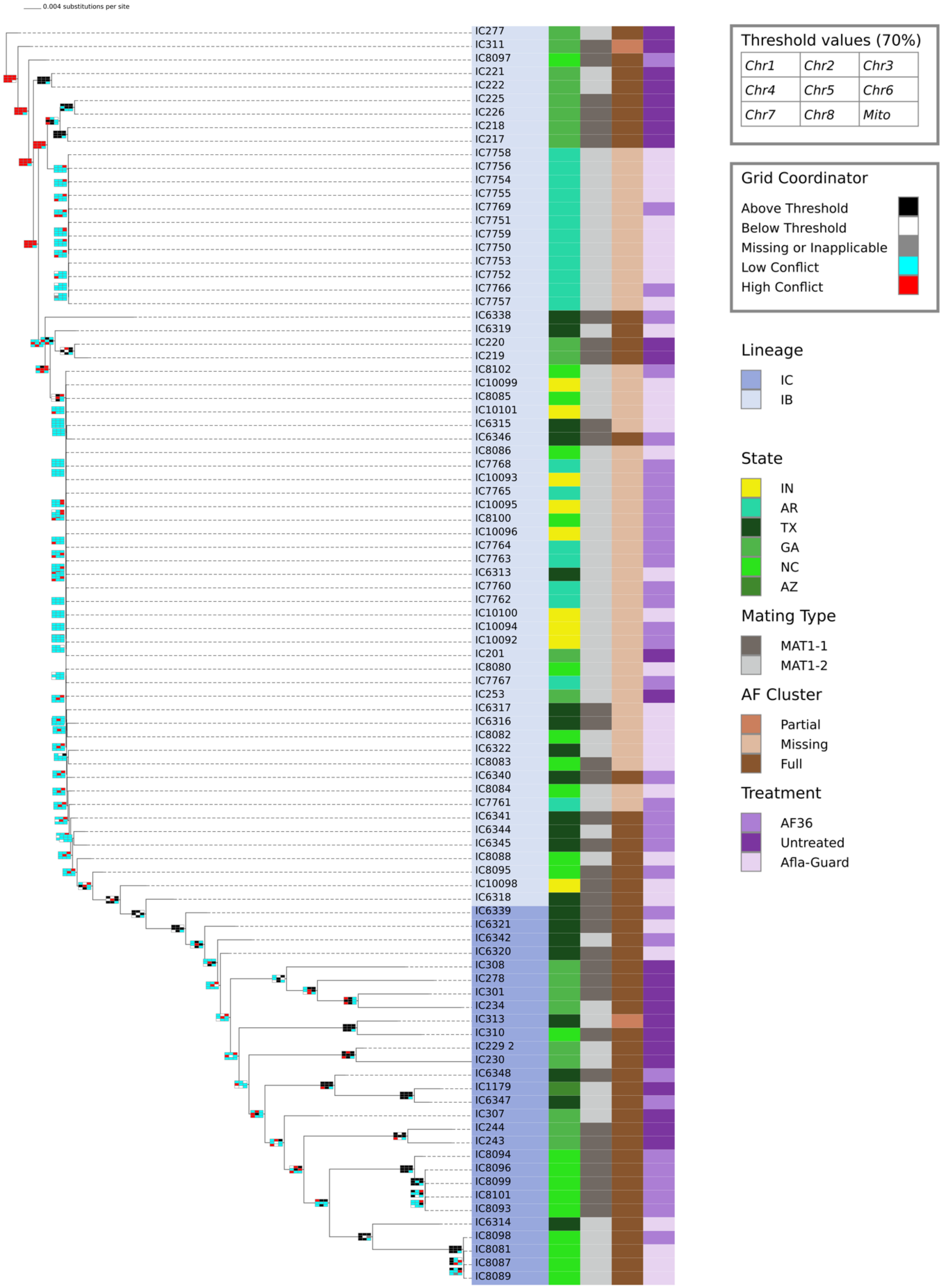

(C) Post 1-year

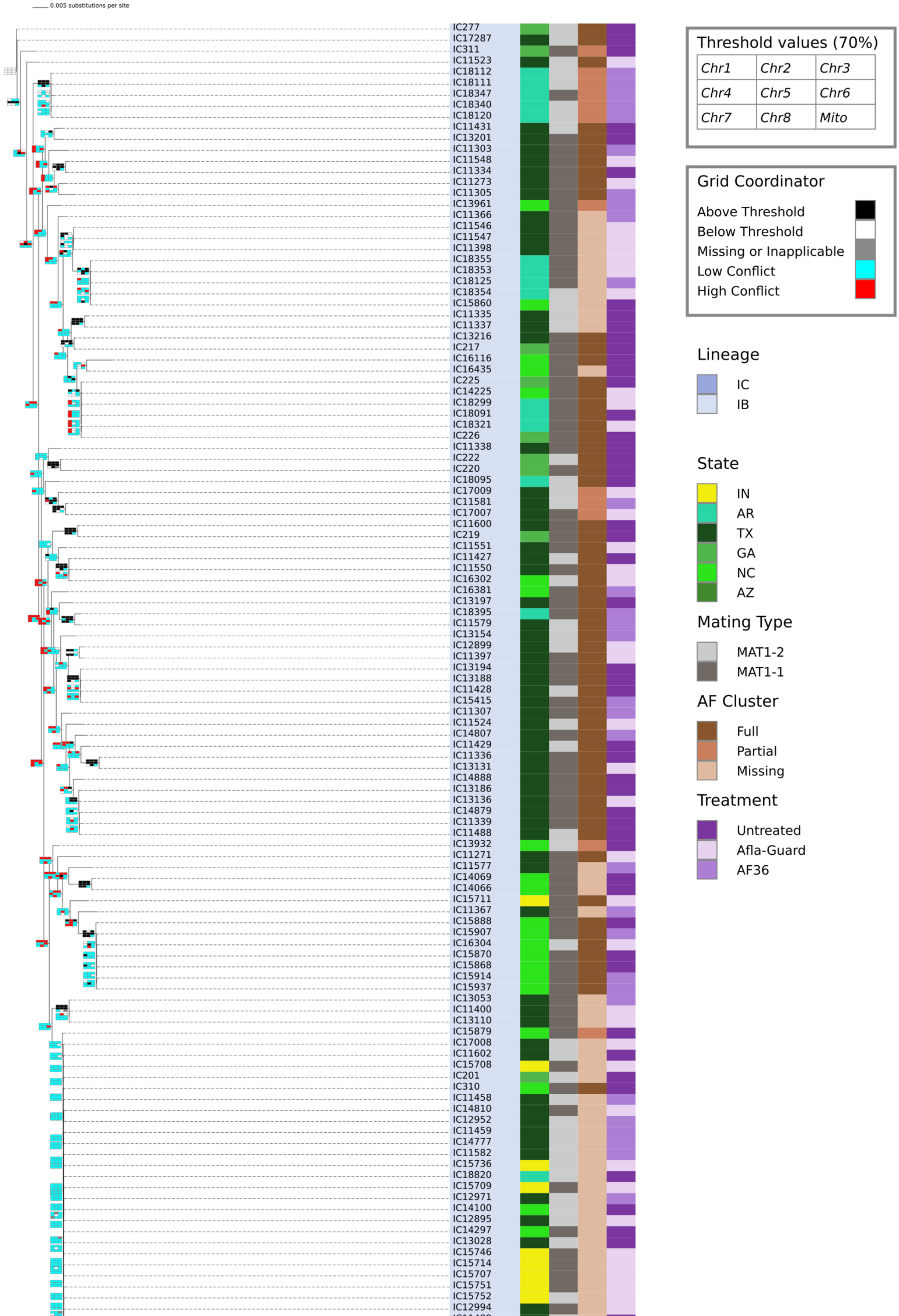
0.005 substitutions per site
IC277
IC17287
IC311
IC11523
IC18112
IC18111
IC18347
IC18340
IC18120
IC11431
IC13201
IC11303
IC11548
IC11334
IC11273
IC11305
IC13961
IC11366
IC11546
IC11547
IC11398
IC18355
IC18353
IC18125
IC18354
IC15860
IC11335
IC11337
IC13216
IC217
IC16116
IC16435
IC225
IC14225
IC18299
IC18091
IC18321
IC226
IC11338
IC222
IC220
IC18095
IC17009
IC11581
IC17007
IC11600
IC219
IC11551
IC11427
IC11550
IC16302
IC16381
IC13197
IC18395
IC11579
IC13154
IC12899
IC11397
IC13194
IC13188
IC11428
IC15415
IC11307
IC11524
IC14807
IC11429
IC11336
IC13131
IC14888
IC13186
IC13136
IC14879
IC11339
IC11488
IC13932
IC11271
IC11577
IC14069
IC14066
IC15711
IC11367
IC15888
IC15907
IC16304
IC15870
IC15868
IC15914
IC15937
IC13053
IC11400
IC13110
IC15879
IC17008
IC11602
IC15708
IC201
IC310
IC11458
IC14810
IC12952
IC11459
IC14777
IC11582
IC15736
IC18820
IC15709
IC12971
IC14100
IC12895
IC14297
IC13028
IC15746
IC15714
IC15707
IC15751
IC15752
IC12994
Threshold values (70%)
Chr1 Chr2 Chr3
Chr4 Chr5 Chr6
Chr7 Chr8 Mito
Grid Coordinator
Above Threshold
Below Threshold
Missing or Inapplicable
Low Conflict
High Conflict
Lineage
IC
IB
State
IN
AR
TX
GA
NC
AZ
Mating Type
MAT1-2
MAT1-1
AF Cluster
Full
Partial
Missing
Treatment
Untreated
Afla-Guard
AF36

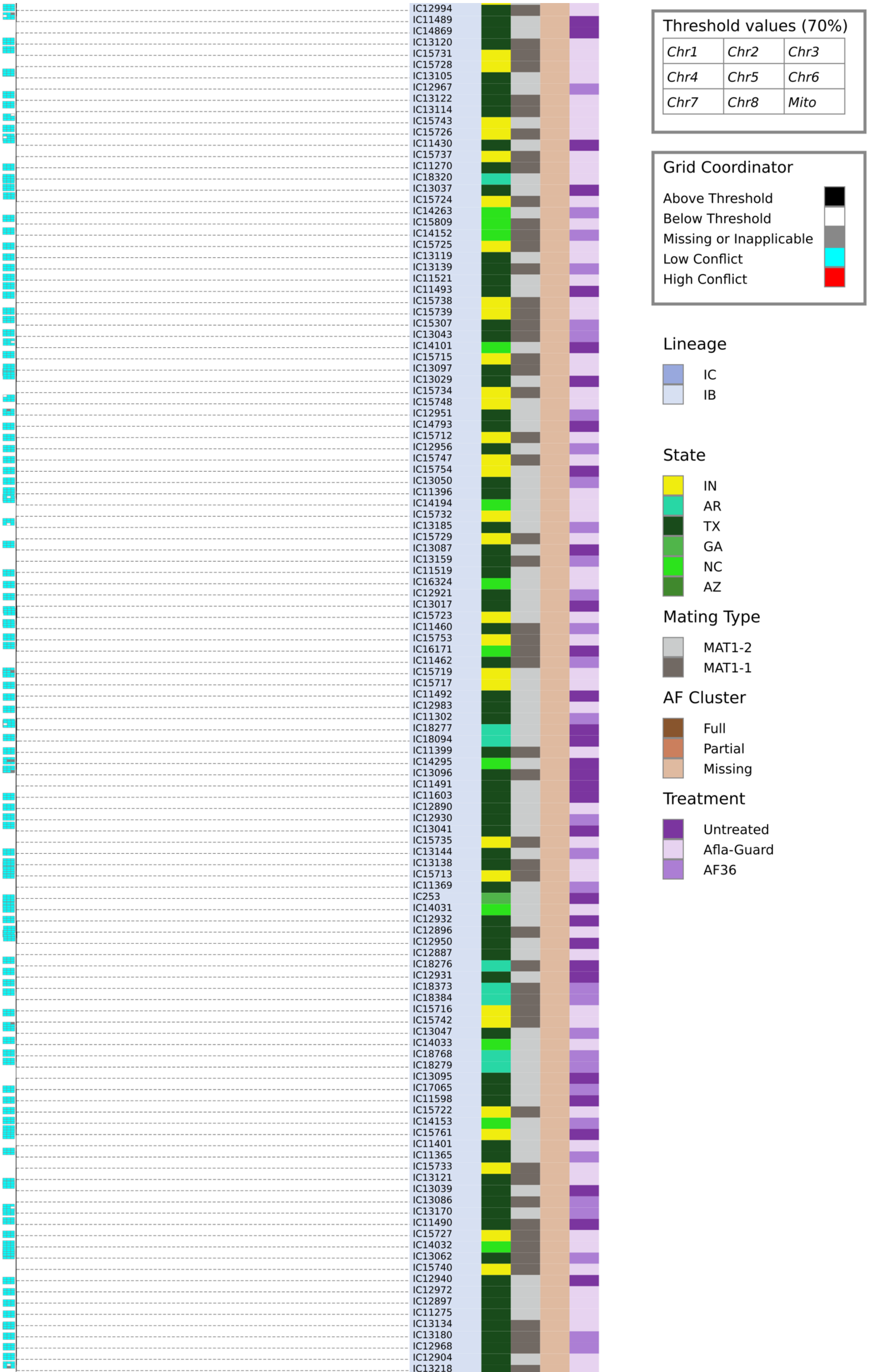
IC12994
IC11489
IC14869
IC13120
IC15731
IC15728
IC13105
IC12967
IC13122
IC13114
IC15743
IC15726
IC11430
IC15737
IC11270
IC18320
IC13037
IC15724
IC14263
IC15809
IC14152
IC15725
IC13119
IC13139
IC11521
IC11493
IC15738
IC15739
IC15307
IC13043
IC14101
IC15715
IC13097
IC13029
IC15734
IC15748
IC12951
IC14793
IC15712
IC12956
IC15747
IC15754
IC13050
IC11396
IC14194
IC15732
IC13185
IC15729
IC13087
IC13159
IC11519
IC16324
IC12921
IC13017
IC15723
IC11460
IC15753
IC16171
IC11462
IC15719
IC15717
IC11492
IC12983
IC11302
IC18277
IC18094
IC11399
IC14295
IC13096
IC11491
IC11603
IC12890
IC12930
IC13041
IC15735
IC13144
IC13138
IC15713
IC11369
IC253
IC14031
IC12932
IC12896
IC12950
IC12887
IC18276
IC12931
IC18373
IC18384
IC15716
IC15742
IC13047
IC14033
IC18768
IC18279
IC13095
IC17065
IC11598
IC15722
IC14153
IC15761
IC11401
IC11365
IC15733
IC13121
IC13039
IC13086
IC13170
IC11490
IC15727
IC14032
IC13062
IC15740
IC12940
IC12972
IC12897
IC11275
IC13134
IC13180
IC12968
IC12904
IC13218
Threshold values (70%)
Chr1 Chr2 Chr3
Chr4 Chr5 Chr6
Chr7 Chr8 Mito
Grid Coordinator
Above Threshold
Below Threshold
Missing or Inapplicable
Low Conflict
High Conflict
Lineage
IC
IB
State
IN
AR
TX
GA
NC
AZ
Mating Type
MAT1-2
MAT1-1
AF Cluster
Full
Partial
Missing
Treatment
Untreated
Afla-Guard
AF36

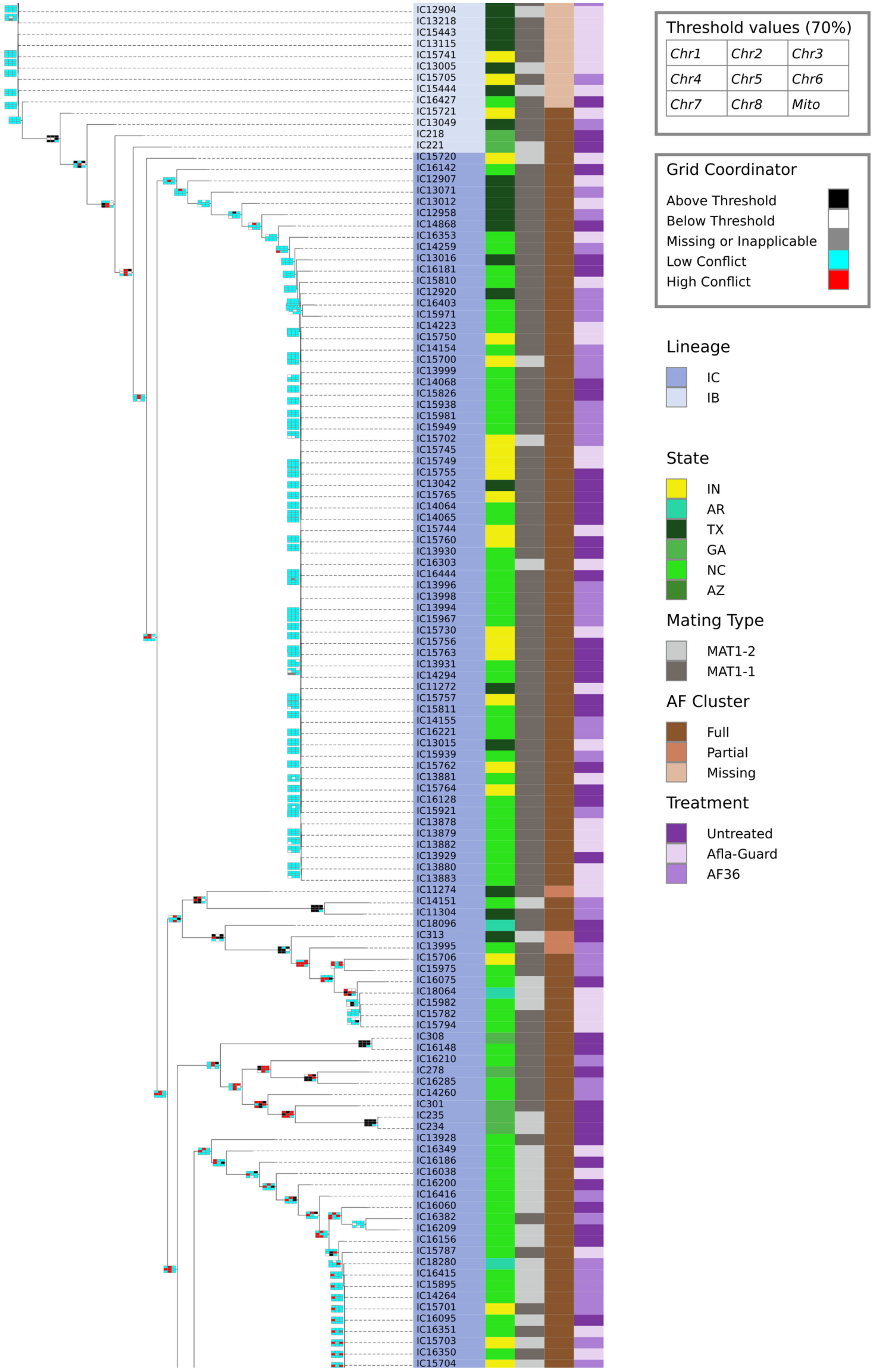
IC12904
IC13218
IC15443
IC13115
IC15741
IC13005
IC15705
IC15444
IC16427
IC15721
IC13049
IC218
IC221
IC15720
IC16142
IC12907
IC13071
IC13012
IC12958
IC14868
IC16353
IC14259
IC13016
IC16181
IC15810
IC12920
IC16403
IC15971
IC14223
IC15750
IC14154
IC15700
IC13999
IC14068
IC15826
IC15938
IC15981
IC15949
IC15702
IC15745
IC15749
IC15755
IC13042
IC15765
IC14064
IC14065
IC15744
IC15760
IC13930
IC16303
IC16444
IC13996
IC13998
IC13994
IC15967
IC15730
IC15756
IC15763
IC13931
IC14294
IC11272
IC15757
IC15811
IC14155
IC16221
IC13015
IC15939
IC15762
IC13881
IC15764
IC16128
IC15921
IC13878
IC13879
IC13882
IC13929
IC13880
IC13883
IC11274
IC14151
IC11304
IC18096
IC313
IC13995
IC15706
IC15975
IC16075
IC18064
IC15982
IC15782
IC15794
IC308
IC16148
IC16210
IC278
IC16285
IC14260
IC301
IC235
IC234
IC13928
IC16349
IC16186
IC16038
IC16200
IC16416
IC16060
IC16382
IC16209
IC16156
IC15787
IC18280
IC16415
IC15895
IC14264
IC15701
IC16095
IC16351
IC15703
IC16350
IC15704
Threshold values (70%)
Chr1
Chr2
Chr3
Chr4
Chr5
Chr6
Chr7
Chr8
Mito
Grid Coordinator
Above Threshold
Below Threshold
Missing or Inapplicable
Low Conflict
High Conflict
Lineage
IC
IB
State
IN
AR
TX
GA
NC
AZ
Mating Type
MAT1-2
MAT1-1
AF Cluster
Full
Partial
Missing
Treatment
Untreated
Afla-Guard
AF36

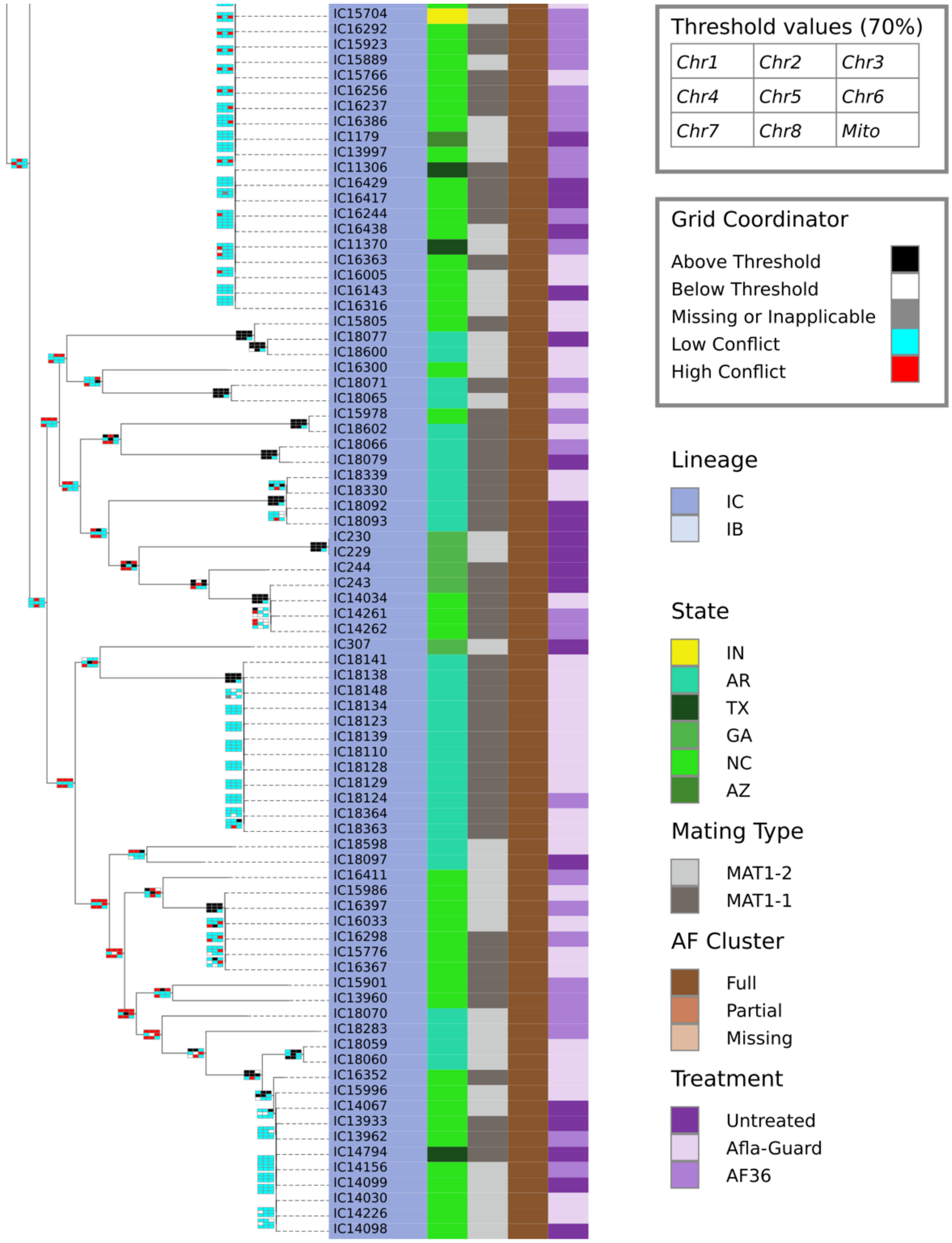
IC15704
IC16292
IC15923
IC15889
IC15766
IC16256
IC16237
IC16386
IC1179
IC13997
IC11306
IC16429
IC16417
IC16244
IC16438
IC11370
IC16363
IC16005
IC16143
IC16316
IC15805
IC18077
IC18600
IC16300
IC18071
IC18065
IC15978
IC18602
IC18066
IC18079
IC18339
IC18330
IC18092
IC18093
IC230
IC229
IC244
IC243
IC14034
IC14261
IC14262
IC307
IC18141
IC18138
IC18148
IC18134
IC18123
IC18139
IC18110
IC18128
IC18129
IC18124
IC18364
IC18363
IC18598
IC18097
IC16411
IC15986
IC16397
IC16033
IC16298
IC15776
IC16367
IC15901
IC13960
IC18070
IC18283
IC18059
IC18060
IC16352
IC15996
IC14067
IC13933
IC13962
IC14794
IC14156
IC14099
IC14030
IC14226
IC14098
Threshold values (70%)
Chr1 Chr2 Chr3
Chr4 Chr5 Chr6
Chr7 Chr8 Mito
Grid Coordinator
Above Threshold
Below Threshold
Missing or Inapplicable
Low Conflict
High Conflict
Lineage
IC
IB
State
IN
AR
TX
GA
NC
AZ
Mating Type
MAT1-2
MAT1-1
AF Cluster
Full
Partial
Missing
Treatment
Untreated
Afla-Guard
AF36

(D) Post 3-years

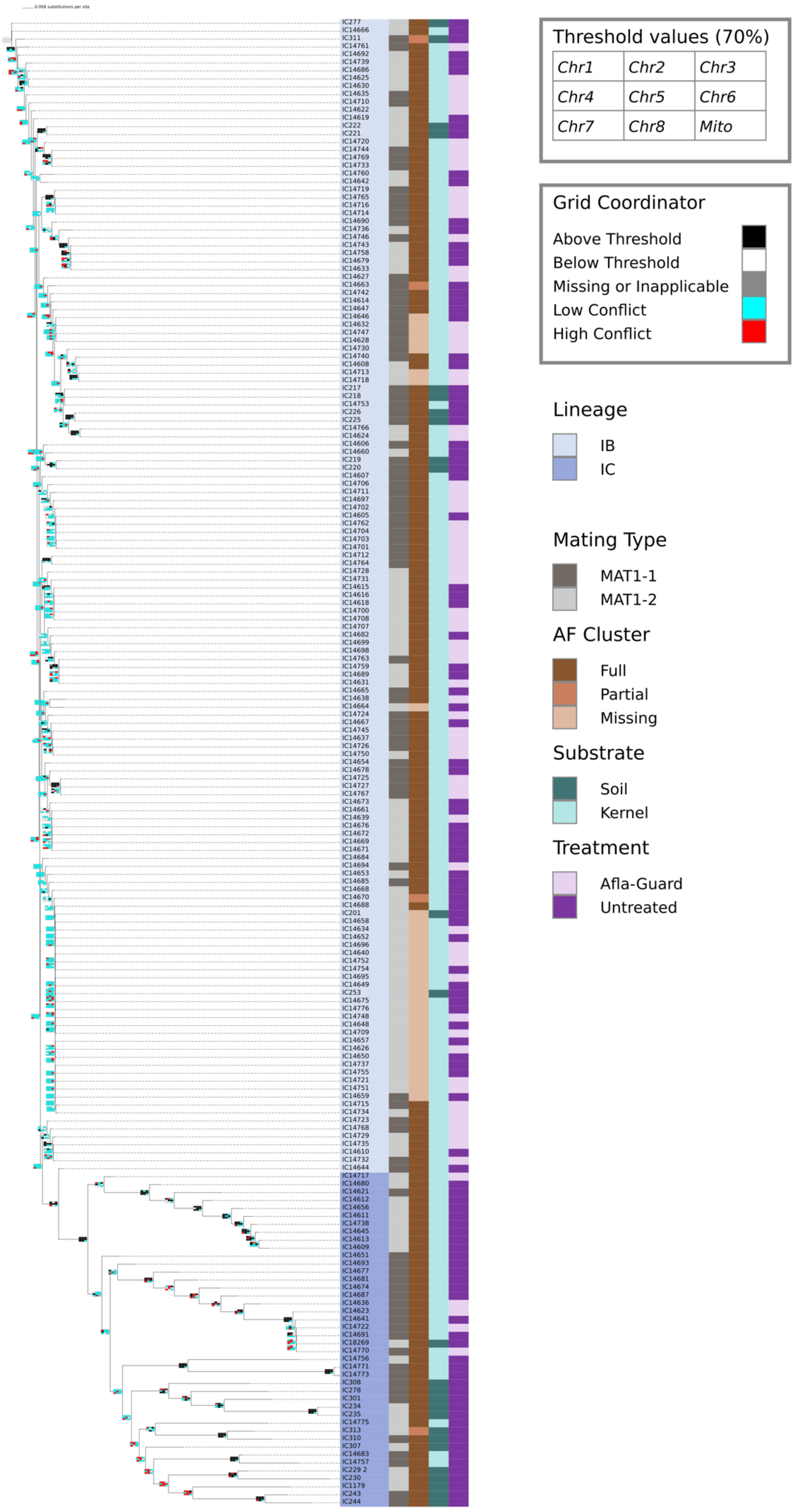

Supplement: S10 Fig — Phylogenetic incongruence between each chromosome and mitochondrial genome phylogeny relative to the total evidence display tree for the four different sampling time points (A-D) is shown using grids on node partitions. Branch lengths on the total evidence tree are drawn to scale and the scale bar is shown at the top. In each grid, bootstrap support values are displayed with each box from left to right representing one of eight chromosomes; the box on the bottom right is for the mitochondrial genome. Colors in grids represent node bipartitions that were supported at a bootstrap support value ≥70% (black color), <70% (white color), and missing or inapplicable (grey color). Phylogenetic incongruency was represented as high conflict (red color) and low conflict (cyan color). Additional attributes (lineage, state, mating type, AF cluster configuration, and substrate/treatment) are shown in columns adjacent to the strain names. (PDF) [file pone.0276556.s010.pdf]
