# Supplementary material for: Asymmetrical lineage introgression and recombination in populations of Aspergillus flavus: Implications for biological control
Source: PLoS One. 2022 Oct 27;17(10):e0276556. doi: 10.1371/journal.pone.0276556 (PMC9620740; doi:10.1371/journal.pone.0276556)

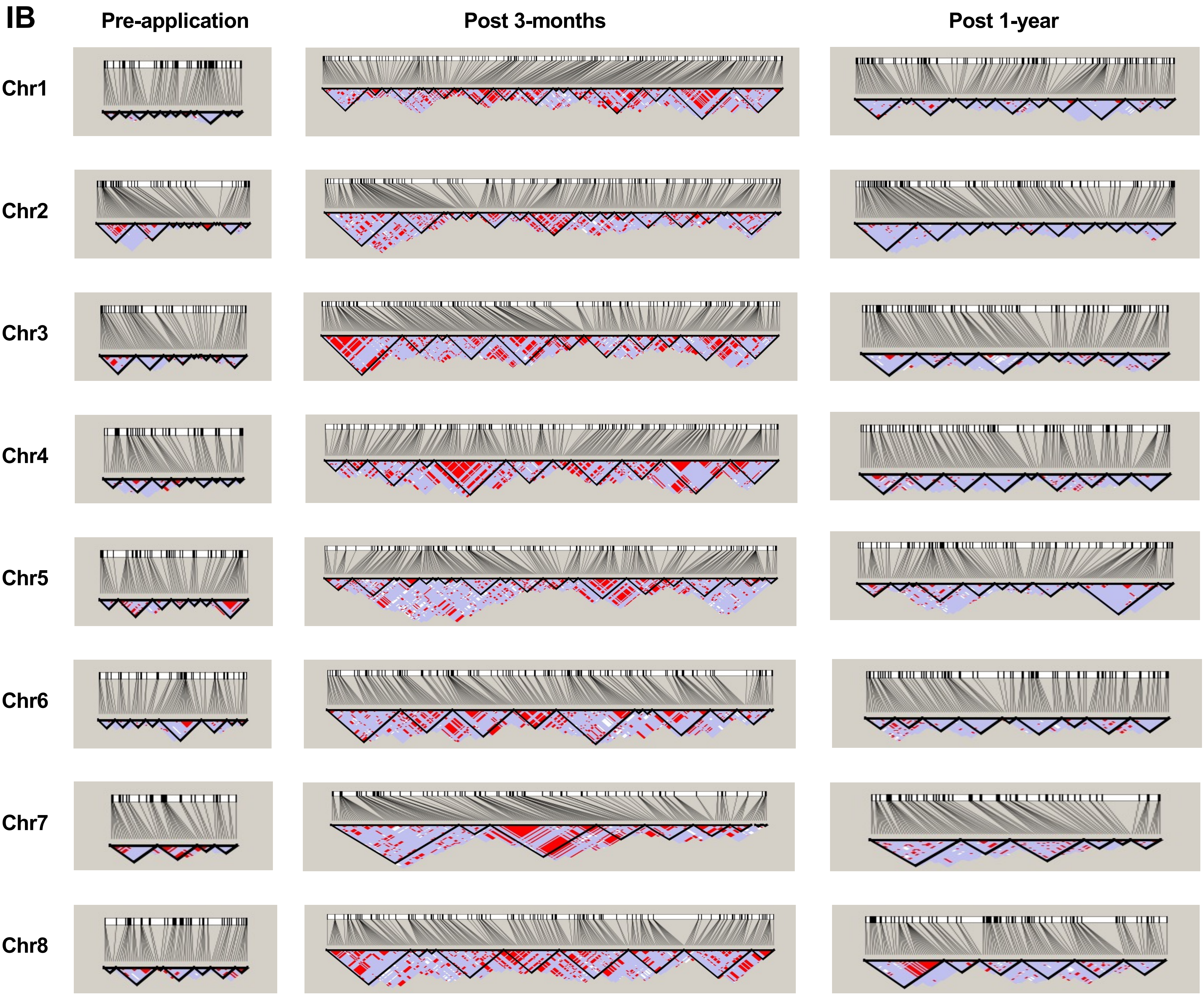
IB
Pre-application
Post 3-months
Post 1-year
Chr1
Chr2
Chr3
Chr4
Chr5
Chr6
Chr7
Chr8

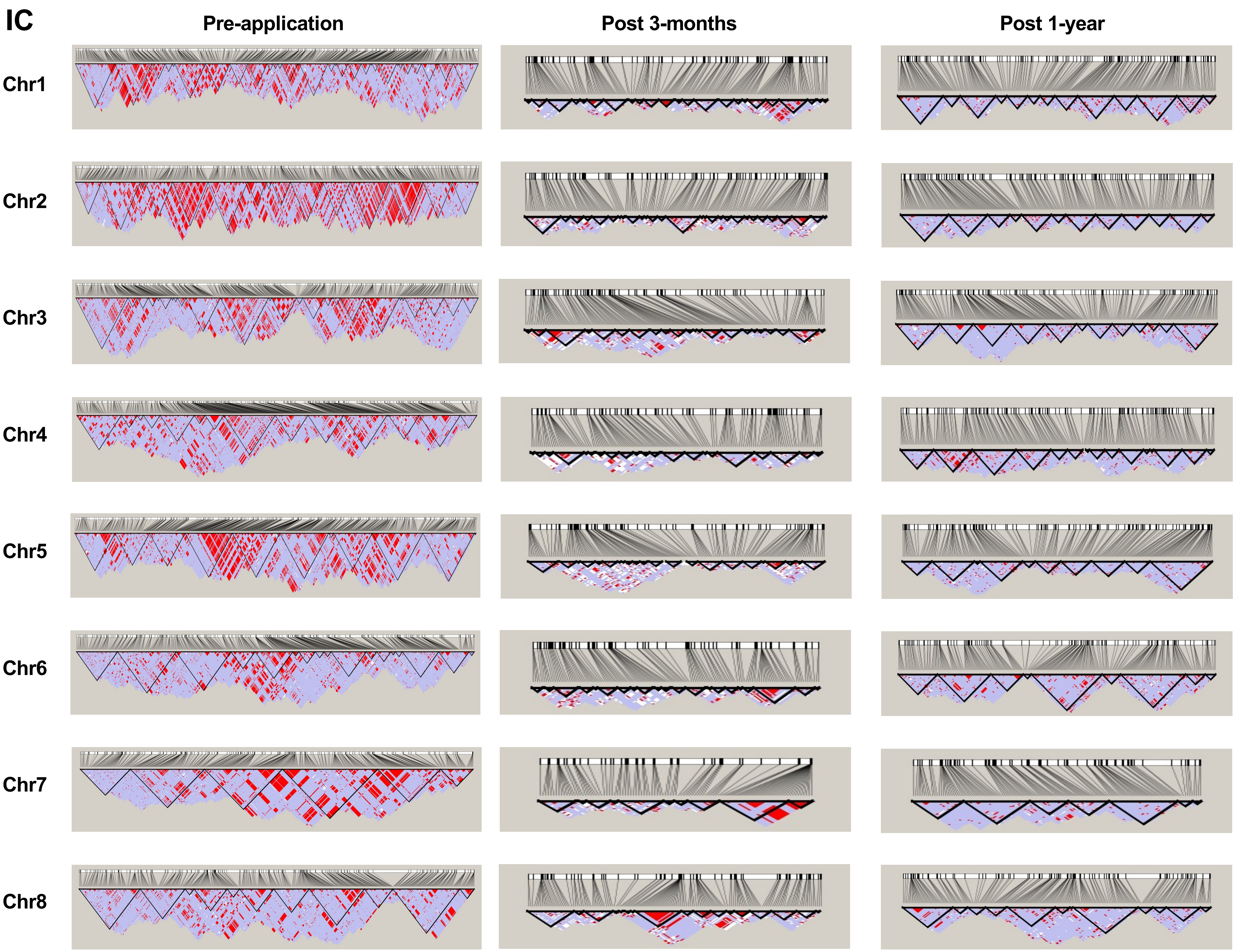
IC
Pre-application
Post 3-months
Post 1-year
Chr1
Chr2
Chr3
Chr4
Chr5
Chr6
Chr7
Chr8

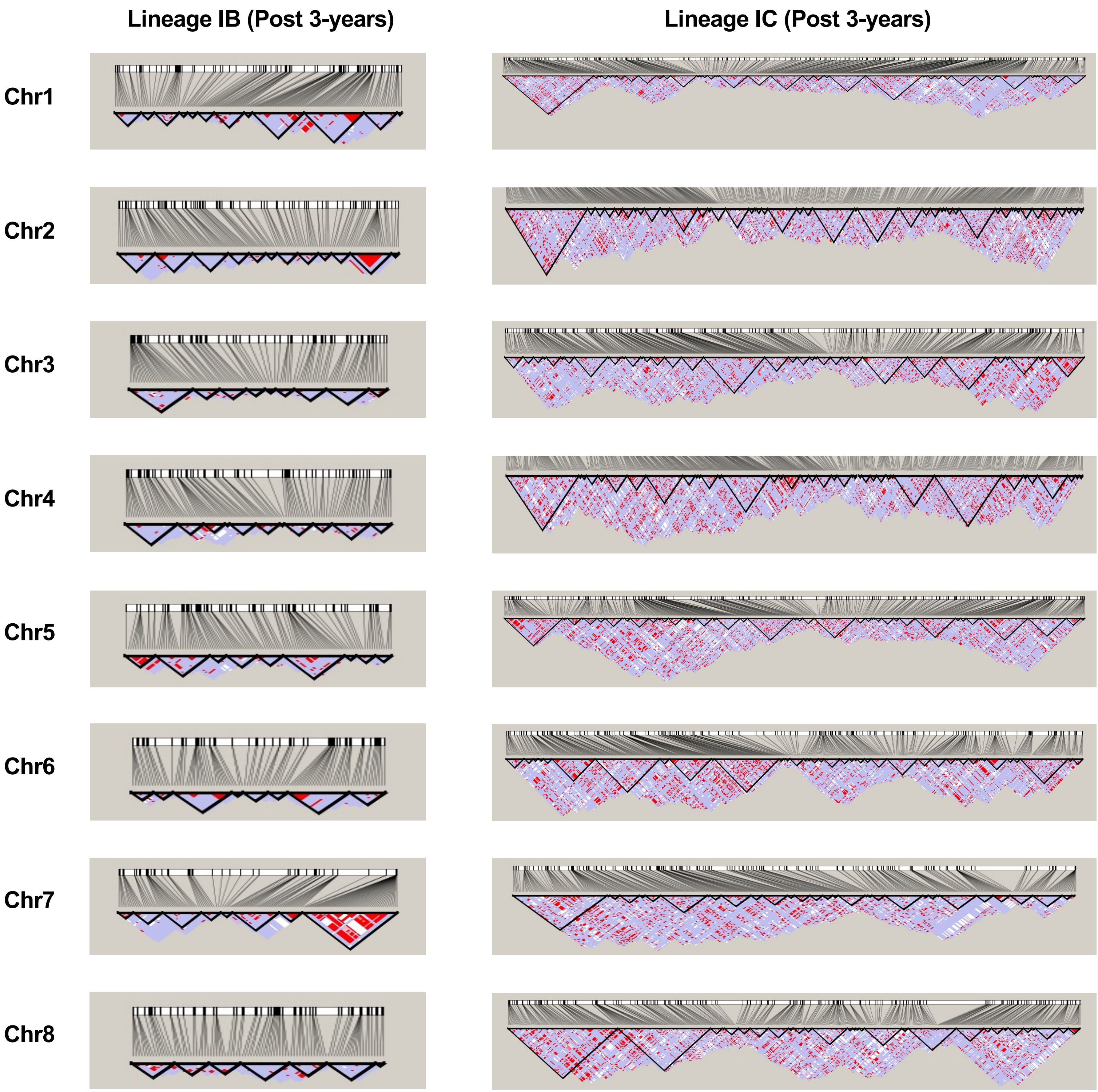

Supplement: S15 Fig — LD plots are displayed using Haploview for each chromosome across four different sampling time points for lineages IB and IC. In each LD plot, black lines outline the edge of the spine of strong LD. In the coloring scheme, red represents strong LD (LOD ≥ 2, D’ = 1), shades of pink/red represent intermediate LD (LOD ≥ 2, D’ < 1), blue represents weak LD (LOD < 2, D’ = 1) and white represents no LD (LOD < 2, D’ < 1). (PDF) [file pone.0276556.s015.pdf]
