# Supplementary material for: Asymmetrical lineage introgression and recombination in populations of Aspergillus flavus: Implications for biological control
Source: PLoS One. 2022 Oct 27;17(10):e0276556. doi: 10.1371/journal.pone.0276556 (PMC9620740; doi:10.1371/journal.pone.0276556)
Supplement: S5 Fig — The eleven sequences highlighted in green share a unique haplotype in the aflM region and are putative hybrids inferred in the multilocus network in S4 Fig and assigned to population A in Drott et al 2020 [37]; the 19 strains highlighted in red for the mfs, trpC and amdS share a distinct multilocus haplotype and are missing the entire aflatoxin gene cluster (except for D82) and assigned to population B in Drott et al 2020 [37]. Strain D82 is a full cluster strain that is a putative inter-lineage recombinant between populations A (= lineage IC) and B (= lineage IB). (PDF) [file pone.0276556.s005.pdf]

Patterns of recombination across *aflM, aflW, mfs, trpC,* and *amdS* for Drott *et al* 2020

	aflM	*aflW*	*mfs*	*trpC*	*amdS*
D1	GAGAGCTTGAGGGAGGTAAAGGCCACTGTAGCGATTTATCGTGCAGA	G--GTTGATTCCGGGCTCCCCTACTTACTATGTCCTGTAG	GGAGGGGTATCGC	TCAACC	GGGCAGCACT
D10	ACAGGGCAACCTGCAGTGCAAACAGTGTAAGTTGCCCGCA--AATTT	-ATGTTGATTCCGGGCTCCCCTACTTACTATGTCCTATAG	GGAGGGGTTTCGC	TCAAAC	GGACAGCACT
D11	ACAGGGCAACCTGCAGTGCAAACAGTGTAAGTTGCCCGCAGCAATTT	-ATGTTGATTCCGGGCTCCCCTACTTACTATGTCCTATAG	GGAGGGGTTTCGC	GCAACC	GGACAGCACT
D12	---	-------------------------------------T-G	GGAGGGGTATCGC	TCAACC	GGACAGCACT
D13	ACAGGGCAACCTGCAGTGCAAACAGTGTAAGTTGCCCGCAGCAATTT	-ATGTTGATTCCGGGCTCCCCTACTTACTATGTCCTATAG	GGAGGGGTTTCGC	TCATCC	GGACAGCACT
D14	GAGGGCTAGAGGGAGGTAAAGGCCACTGTAGCGATTTATCGTGCAGA	GATGTTGATTCCGGGCTCCTCTACTTACTATGGCCTATAG	GGAGGGGTAGCGC	GCAACC	GGATGGTACT
D15	---	--	GGAGGGGTATCGC	TCAACC	GGACAGCACT
D16	ACAGGGCAACCTGCATTGCAAACAGTGTAAGTTGCCCGCAGCAATTT	GGCTGCAAGCCTATCTTTTCACGACTGTCGGGTTTCATGA	GGAGGGGAATCGC	TCAACC	GGACAGCACT
D17	GAGGGCTAGAGGGAGGTAAAGGCCACTGTAGCGATTTATCGTGCAGA	G------A--C-----T--CCTACTTACTATATCCTATAG	GGAGGGATATCGC	GCAACC	GGACAGCACT
D18	ACAGGGCAACCTGCAGTGCAAACAGTGTAAGTTGCCCGCAGCAATTT	-ATGTTGATTCCGGGCTCCCCTACTTACTATGTCCTATAG	GGAGGGGTTTCGC	TTGACC	GGACATCACT
D19	---	--	GGAGGGGTATCGC	TCAACC	GGACAGCACT
D2	ACAGGGCAACCTGCAGTGCAAACAGTGTAAGTTGCCCGCAGCAATTT	GATGTTGATTCCGGGCTCCCCTACTTACTATGTCCTGTAG	GGAGGGGTATCGC	GCAACC	GGGCAGCACT
D20	---	GGCTGCAAGCCTATCTTTTCACGACTGTCGGGTTTCATGA	GGAAGGGAATCTC	TCAACC	GGACAGCACT
D21	ACAGGGCAACCTGCAGTGCAAACAGTGTAAGTTGCCCGCATCAATTT	GGCTGCAAGCCTATCTTTTCACGACTGTTGGGTTTCATGA	GGTGGGGAATCGC	TCAACC	GGACAGCAGT
D22	GAGGGCTAGAGGGAGGTAAAGGCCACTGTAGCGATTTATCGTGCAGA	GATGTTGATTCCGGGCTCCTCTACTTACTATGGCCTATAG	GGAGGGGTAGCGC	GCAACC	GGATGGTACT
D23	---	--	GGAGGGGAATCTC	TCAACC	GGACAGCACT
D24	GAGGGCTAGAGGGAGGTAAAGGCCACTGTAGCGATTTATCGTGCAGA	GATGTTGATTCCGGGCTCCCCTACTTACTATATCCTATAG	GGAGGGATATCGC	GCAACC	GGATGGTACT
D25	ACAGGGCAACCTGCATTGCAAACAGTGTAAGTTGCCCGCAGCAATTT	GGCTGCAAGCCTATCTTTTCACGACTGTCGGGTTTCATGA	GGAGGGGAATCGC	TCAACC	GGACAGCACT
D26	GAGGGCTAGAGGGAGGTAAAGGCCACTGTAGCGATTTATCGTGCAGA	-------A--C-----T--------------GTCCTATAG	GGAGGGGTAGCGC	GCAACC	GGACAGCACT
D27	GAGGGCTAGAGGGAGGTAATGGCCACTGTAGCGATTTATCGTGCAGA	-ATGTTGATTTCGGGCTCCCCTACTTACTATGTCCTATAG	GAAGGGGTATCGC	TCATCC	GGGCAGCACT
D28	ACAGGGCAACCTGCATTGCAAACAGTGTAAGTTGCCCGCAGCAATTT	GGCTGCAAGCCTATCTTTTCACGACTGTCGGGTTTCATGA	GGAGGGGAATCGC	TCAACC	GGACAGCACT
D29	GAGGGCTAGAGGGAGGTAAAGGCCACTGTAGCGATTTATCGTGCAGA	G--GTTGATTCCGGGCTCCCCTACTTACTATATCCTATAG	GGAGGGATATCGC	GCAACC	GGACAGCACT
D3	---	GGCTGCAAGCCTATCTTTTCACGACTGTCGGGTTTCATGA	GGAAGGGAATCTC	TCAACC	GGACAGCACT
D30	---	--	GGAGGGGTATCGC	TCAACC	GGACAGCACT
D31	---	--	GGAGGGGTATCGC	TCAACC	GGACAGCACT
D32	ACAGGGCAACCTGCATTGCAAACAGTGTAAGTTGCCCGCAGCAATTT	GGCTGCAAGCCTATCTTTTCACGACTGTCGGGTTTCATGA	GGAGGGGAATCGC	TCAACC	GGACAGCACT
D33	ACAGGGCAACCTGCAGTGCAAACAGTGTAAGTTGCCCGCAGCAATTT	---GTTGATTCCGGGCGCCCCTACTTACTATGTCCTATAG	GGAGGGGTTTCGC	TCAACC	GGACAGCACT
D34	---	--	GGAGGGGTATCGC	TCAACC	GGACAGCACT
D35	ACAGGGCAACCTGCATTGCAAACAGTGTAAGTTGCCCGCAGCAATTT	GGCTGCAAGCCTATCTTTTCACGACTGTCGGGTTTCATGA	GGAGGGGAATCGC	TCAACC	GGACAGCACT
D36	GAGGGCTAGAGGGAGGTAATGGCCACTGTAGCGATTTATCGTGCAGA	AATGTTGATTCCGGGCTCCCCTACTTACTATATCCTATAG	AAAGAGGTATCGC	TCATCC	GGGCAGCACT
D37	GAGGGCTAGAGGGAGGTAAAGGCCACTGTAGCGATTTATCGTGCAGA	---GTTACGTCCGGGCTCCCCTACTTACTATGTCCTATAG	GGAGGGGTTTCGC	TCATCC	GGGCAGCACT
D38	GAGGGCTAGAGGGAGGTAAAGGCCACTGTAGCGATTTATCGTGCAGA	---GTTACGTCCGGGCTCCCCTACTTACTATGTCCTATAG	GGAGGGGTTTCGC	TCATCC	GGGCAGCACT
D39	GAGGGCTAGAGGGAGGTAATGGCCACTGTAGCGATTTATCGTGCAGA	-ATGTTGATTTCGGGCTCCCCTACTTACTATGTCCTATAG	GAAGGGGTATCGC	TCAACA	GGACAGCACT
D4	ACAGGGCAACCTGCAGTGCAAACA--G----------G--G-A----	-ATGTTGATTCCGGGCTCCCCTACTTACTATGTCCTATAG	GGAGGGGTTTCGC	TTGACC	GGACAGCACT
D40	GAGGGCTAGAGGGAGGTAATGGCCACTGTAGCGATTTATCGTGCAGA	-------A--------T--CCTACTTACTATGTCCTATAG	GAAGGGGTATCGC	TCAACC	GGACAGCACT
D41	GAGGGCTAGAGGGAGGTAATGGCCACTGTAGCGATTTATCGTGCAGA	---GTTGATTTCGGGCTCCCCTACTTACTATGTCCTATAG	GGAGGGGTATCGC	TCAACC	GGACAGCACT
D42	ACAGGGCAACCTGCATTGCAAACAGTGTAAGTTGCCCGCAGCAATTT	GGCTGCAAGCCTATCTTTTCACGACTGTCGGGTTTCATGA	GGAGGGGAATCGC	TCAACC	GGACAGCACT
D43	---	GGCTGCAAGCCTATCTTTTCACGACTGTCGGGTTTCATGA	GGAAGGGAATCTC	TCAACC	GGACAGCACT
D44	---	GGCTGCAAGCCTATCTTTTCACGACTGTCGGGTTTCATGA	GGAAGGGAATCTC	TCAACC	GGACAGCACT
D45	GAGGGCTAGAGGGAGGTAATGGCCACTGTAGCGATTTATCGTGCAGA	AATGTTGATTCCGGGCTCCCCTACTTACTATATCCTATAG	AAAGAGGTATCGC	TCATCC	GGACAGCACT
D46	GAGGGCTAGAGGGAGGTAATGGCCACTGTAGCGATTTATCGTGCAGA	GATGTTGATTCCGGGCTCCTCTACTTACTATGTCCTATAG	GAAGGGGTATCGC	GCAACC	GGGCAGCACT
D47	ACAGGGCAACCTGCAGTGCAAACAGTGTAAGTTGCCCGCAGCAATTT	GATGTTGATTCCGGGCTCCCCTACTTACTATGTCCTGTAG	GGAGGGGTATCGC	TTGACC	GGACAGCACT
D48	---	GGCTGCAAGCCTATCTTTTCACGACTGTCGGGTTTCATGA	GGAGGGGAATCTC	TCAACC	GGACAGCACT
D49	ACAGGGCAACCTGCAGTGCAAACAGTGTAAGTTGCCCGCAGCAATTT	---GTTGATTCCGGGCTCCCCTACTTACTATGTCCTATAG	GGAGGGGTTTCGC	TCAACC	GGACAGCACT
D5	ACAGGGCAACCTGCATTGCAAACAGTGTAAGTTGCCCGCAGCAATTT	GGCTGCAAGCCTATCTTTTCACGACTGTCGGGTTTCATGA	GGAGGGGAATCGC	TCAACC	GGACAGCACT
D50	--------------------------G----------G--G-A----	--	GGAGGGGTATCGC	TCAACC	GGACAGCACT
D51	ACAGGGCAACCTGCATTGCAAACAGTGTAAGTTGCCCGCAGCAATTT	GGCTGCAAGCCTATCTTTTCACGACTGTCGGGTTTCATGA	GGAGGGGAATCGC	TCAACC	GGACAGCACT
D52	---	--	GGAGGGGTATCGC	TCAACC	GGACAGCACT
D53	ACAGGGCAACCTGCATTGCAAACAGTGTAAGTTGCCCGCAGCAATTT	GGCTGCAAGCCTATCTTTTCACGACTGTCGGGTTTCATGA	GGAGGGGAATCGC	TCAACC	GGACAGCACT
D54	---	--	GGAGGGGTATCGC	TCAACC	GGACAGCACT
D55	ACAGGGCAACCTGCAGTGCAAACAGTGTAAGTTGCCCGCATCAATTT	GGCTGCAAGCCTATCTTTTCACGACTGTTGGGTTTCATGA	GGTGGGGAATCGC	TCAACC	GGACAGCACT
D56	---	--	GGAGGGGTATCGC	TCAACC	GGACAGCACT
D57	GAGGGCTAGAGGGAGGTAAAGGCCACTGTAGCGATTTATCGTGCAGA	GATGTTGATTCCGGGCTCCTCTACTTACTATGTCCTATAG	GAAGGGGTATTGT	TCAACC	GGACAGCACT
D58	ACAGGGCAACCTGCAGTGCAAACAGTGTAAGTTGCCCGCAGCAATTT	-ATGTTGATTCCGGGCTCCCCTACTTACTATGTCCTATAG	GGAGGGGTTTCGC	TCAACC	GGACAGCACT
D59	---	-------------------------------GTCCTAT-G	GGAGGGGTATCGC	TCAACC	GGACAGCACT
D6	---	--	GGAGGGGTATCGC	TCAACC	GGACAGCACT
D60	GAGGGCTAGAGGGAGGTAATGGCCACTGTAGCGATTTATCGTGCAGA	---GTTGATTTCGGGCTCCCCTACTTACTATGTCCTATAG	GAAGGGGTATCGC	GCAACC	GGACATCACT
D61	GAGGGCTAGAGGGAGGTAATGGCCACTGTAGCGATTTATCGTGCAGA	---GTTGATTTCGGGCTCCCCTACTTACTATGTCCTATAG	GAAGGGGTATCGC	GCAACC	AGACAGCACC
D62	GAGGGCTAGAGGGAGGTAATGGCCACTGTAGCGATTTATCGTGCAGA	---GTTGATTTCGGGCTCCCCTACTTACTATGTCCTATAG	GAAGGGGTATCGC	TCAACC	GGACAGCACT
D63	GAGGGCTAGAGGGAGGTAATGGCCACTGTAGCGATTTATCGTGCAGA	---GTTGATTTCGGGCTCCCCTACTTACTATGTCCTATAG	GGAGGGGTTTCGC	TCAAAC	GGACAGCACT
D64	GAGGGCTAGAGGGAGGTAAAGGCCACTGTAGCGATTTATCGTGCAGA	GATGTTGATTCCGGGCTCCCCTACTTACTATGTCCTGTAG	GGAGGGGTATCGC	TTGACC	GGACATCACT
D65	---	GGCTGCAAGCCTATCTTTTCACGACTGTCGGGTTTCATGA	GGAAGGGAATCTC	TCAACC	GGACAGCGCT
D66	ACAGGGCAACCTGCATTGCAAACAGTGTAAGTTGCCCGCAGCAATTT	GGCTGCAAGCCTATCTTTTCACGACTGTCGGGTTTCATGA	GGAGGGGAATCGC	TCAACC	GGACAGCACT
D67	GAAGGCTAGAGGTCGGCAAAGGTCACGGTCACGATTTGTCGTACAGA	GGCTGCAAGCCTATCTTTTCACGACCGTCGGGTTTCATGA	GGAGGAGTATCGC	TCAACC	GGACAGCACT
D68	ACAGGGCAACCTGCATTGCAAACAGTGTAAGTTGCCCGCAGCAATTT	GGCTGCAAGCCTATCTTTTCACGACTGTCGGGTTTCACGA	GGAGGGGAATCGC	TCAACC	GGACAGCACT
D69	GAGGGCTAGAGGGAGGTAAAGGCCACTGTAGCGATTTATCGTGCAGA	-ATGTTGATTCCGGGCTCCCCTACTTACTATATCCTATAG	GGAGGGATATCGC	GCAACC	GGACAGCACT
D7	GAGGGCTAGAGGGAGGTAATGGCCACTGTAGCGATTTATCGTGCAGA	AATGTTGATTCCGGGCTCCCCTACTTACTATATCCTATAG	AAAGAGGTATCGC	GCAACC	GGACAGCACT
D70	---	GGCTGCAAGCCTATCTTTTCACGACTGTCGGGTTTCATGA	GGAGGGGAATCTC	TCAACC	GGACAGCACT
D71	GAGGGCTAGAGGGAGGTAAAGGCCACTGTAGCGATTTATCGTGCAGA	----------------TCCTCTACTTACTATGTCCTATAG	GGAGGGGTAGCGC	GCAACC	GGACAGCACT
D72	GAGGGCTAGAGGGAGGTAAAGGCCACTGTAGCGATTTATCGTGCAGA	G--GTTGATTCCGGGCTCCCCTACTTACTATATCCTATAG	GGAGGGATATCGC	GCAACC	GGACAGCACT
D73	GAGGGCTAGAGGGAGGTAAAGGCCACTGTAGCGATTTATCGTGCAGA	GATGTTGATTCCGGGCTCCTCTACTTACTATGGCCTATAG	GGAGGGGTAGCGC	GCAACC	GGATGGTACT
D74	ACAGGGCAACCTGCATTGCAAACAGTGTAAGTTGCCCGCAGCAATTT	GGCTGCAAGCCTATCTTTTCACGACTGTCGGGTTTCATGA	GGAGGGGAATCGC	TCAACC	GGACAGCACT
D75	ACAGGGCAACCTGCATTGCAAACAGTGTAAGTTGCCCGCAGCAATTT	GGCTGCAAGCCTATCTTTTCACGACTGTCGGGTTTCATGA	GGAGGGGAATCGC	TCAACC	GGACAGCACT
D76	ACAGGGCAACCTGCATTGCAAACAGTGTAAGTTGCCCGCAGCAATTT	GGCTGCAAGCCTATCTTTTCACGACTGTCGGGTTTCATGA	GGAGGGGAATCGC	TCAACC	GGACAGCACT
D77	ACAGGGCAACCTGCATTGCAAACAGTGTAAGTTGCCCGCAGCAATTT	GGCTGCAAGCCTATCTTTTCACGACTGTCGGGTTTCATGA	GGAGGGGAATCGC	TCAACC	GGACAGCACT
D78	GAGGGCTAGAGGGAGGTAAAGGCCACTGTAGCGATTTATCGTGCAGA	GATGTTGATTCCGGGCTCCCCTACTTACTATATCCTATAG	GGAGGGATATCGC	GCAACC	GGACAGCACT
D79	---	GGCTGCAAGCCTATCTTTTCACGACTGTCGGGTTTCATGA	GGAGGGGAATCTC	TCAACC	GGACAGCACT
D8	---	--	GGAGGGGTATCGC	TCAACC	GGACAGCACT
D80	ACAGAGCAACCTGCATTGCAAACAGTGTAAGTTGCCCGCAGCAATTT	GGCTGCAAGCCTATCTTTTCACGACTGTCGGGTTTCATGA	GGAGGGGAATCGC	TCAACC	GGACAGCACT
D81	ACAGGGCAACCTGCATTGCAAACAGTGTAAGTTGCCCGCAGCAATTT	GGCTGCAAGCCTATCTTTTCACGACTGTCGGGTTTCATGA	GGAGGGGAATCGC	TCAACC	GGACAGCACT
D82	ACAGGGCAACCTGCAGTGCAAACAGTGTAAGTTGCCCGCAGCAATTT	-------A--C-----T--CCTACTTACTATGTCCTATAG	GGAGGGGTATCGC	TCAACC	GGACAGCACT
D84	ACAGGGCAACCTGCATTGCAAACAGTGTAAGTTGCCCGCAGCAATTT	GGCTGCAAGCCTATCTTTTCACGACTGTCGGGTTTCATGA	GGAGGGGAATCGC	TCAACC	GGACAGCACT
D85	ACAGGGCAACCTGCATTGCAAACAGTGTAAGTTGCCCGCAGCAATTT	GGCTGCAAGCCTATCTTTTCACGACTGTCGGGTTTCATGA	GGAGGGGAATCGC	TCAACC	GGACAGCACT
D86	GAGGGCTAGAGGGAGGTAATGGCCACTGTAGCGATTTATCGTGCAGA	---GTTGATTTCGGGCTCCCCTACTTACTATGTCCTATAG	GAAGGGGTATCGC	GCAACC	GGACAGCACT
D87	ACAGGGCAACCTGCAGTGCAAACAGTGTAAGTTGCCCGCAGCAATTT	GATGTTGATTCCGGGCTCCCCTACTTACTATGTCCTGTAG	GGAGGGGTATCGC	TCAACC	GGACAGTACT
D88	ACAGGGCAACCTGCATTGCAAACAGTGTAAGTTGCCCGCAGCAATTT	GGCTGCAAGCCTATCTTTTCACGACTGTCGGGTTTCATGA	GGAGGGGAATCGC	TCAACC	GGACAGCACT
D89	---	--	GGAGGGGTATCGC	TCAACC	GGACAGCACT
D9	GAGGGCTAGAGGGAGGTAATGGCCACTGTAGCGATTTATCGTGCAGA	-ATGTTGATTTCGGGCTCCCCTACTTACTATGTCCTATAG	GAAGGGGTATCGC	TCAACC	GAACAGCACT
D90	---	--	GGAGGGGTATCGC	TCAACC	GGACAGCACT
D91	---	--	GGAGGGGTATCGC	TCAACC	GGACAGCACT
D92	---	--	GGAGGGGTATCGC	TCAACC	GGACAGCACT
D93	ACAGGGCAACCTGCATTGCAAACAGTGTAAGTTGCCCGCAGCAATTT	GGCTGCAAGCCTATCTTTTCACGACTGTCGGGTTTCATGA	GGAGGGGAATCGC	TCAACC	GGACAGCACT
D94	---	--	GGAGGGGTATCGC	TCAACC	GGACAGCACT
D95	ACAGGGCAACCTGCAGTGCAAACAGTGTAAGTTGCCCGCAGCAATTT	---GTTGATTCCGGGCTCCCCTACTTACTATGTCCTATAG	GGAGGGGTTTCGC	GCAACC	GGGCAGCACT
